# Novel Thiourea Ligands—Synthesis, Characterization and Preliminary Study on Their Coordination Abilities

**DOI:** 10.3390/molecules29204906

**Published:** 2024-10-16

**Authors:** Stanislava E. Todorova, Rusi I. Rusew, Zhanina S. Petkova, Boris L. Shivachev, Vanya B. Kurteva

**Affiliations:** 1 Institute of Organic Chemistry with Centre of Phytochemistry, Bulgarian Academy of Sciences, Acad. G. Bonchev Str., bl. 9, 1113 Sofia, Bulgariazhanina.petkova@orgchm.bas.bg (Z.S.P.); 2 Institute of Mineralogy and Crystallography “Acad. Ivan Kostov”, Bulgarian Academy of Sciences, Acad. G. Bonchev Str., bl. 107, 1113 Sofia, Bulgaria; 3 Centre of Competence “Sustainable Utilization of Bio-Resources and Waste of Medicinal and Aromatic Plants for Innovative Bioactive Products” (CoC BioResources), Acad. G. Bonchev Str., bl. 9, 1113 Sofia, Bulgaria

**Keywords:** polydentate ligands, unsymmetrical and symmetrical thioureas, NMR, XRD, ITC

## Abstract

Two series of polydentate *N*,*O*,*S*-ligands containing thiourea fragments attached to a *p*-cresol scaffold, unsymmetrical mono-acylated bis-amines and symmetrical bis-thioureas, are obtained by common experiments. It is observed that the reaction output is strongly dependent on both bis-amine and thiocarbamic chloride substituents. The products are characterized by 1D and 2D NMR spectra in solution and by single crystal XRD. A preliminary study on the coordination abilities of selected products is performed by ITC at around neutral media.

## 1. Introduction

**Figure 1:**
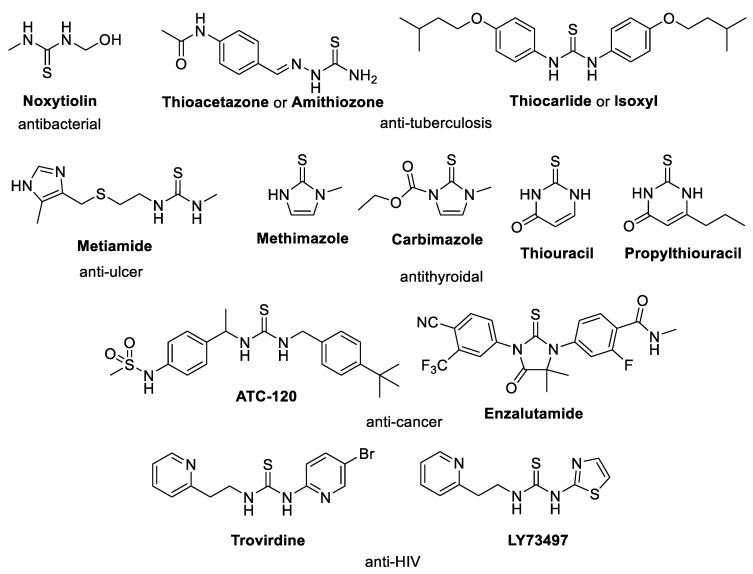
Available on the market medicinal preparations containing thiourea moieties.

Thiourea derivatives represent a broad family of molecules containing the N-(C=S)-N fragments, which have found wide applications in almost all areas of chemistry [1,2,3,4]. Certain representatives have shown efficiency as chemosensors [5,6,7], organogelators [8], corrosion inhibitors [9], nanocrystals [10], insecticidals [11] etc. Compounds possessing thiourea moiety have found essential applications in pharmaceutical industry [12,13,14], as building blocks [15,16,17,18] and catalysts [19,20,21,22,23,24] in various organic transformations, and have exhibited a broad range of bioactivities [13,25,26,27], like antimicrobial [28,29,30], anti-tuberculosis [31], anti-HIV [32], anti-cancer [33,34,35] etc. Among the vast variety of products, some have shown remarkable activities and are clinically prescribed against various diseases (Figure 1), such as local antibacterial agent noxytiolin [36,37]; anti-tuberculosis drugs thioacetazone and thiocarlide [38,39,40]; agent for treatment of acid-reflux disorders and peptic ulcer disease metiamide [41]; medications that treat hyperthyroidism carbimazole, methimazole and propylthiouracil [42,43]; anti-cancer drugs ATC-120 and enzalutamide [44,45,46] and potent nonnucleoside HIV inhibitors trovirdine and LY73497 [47,48,49].

From a coordination chemistry point of view, sulphur compounds have displayed spectacular efficiency [50,51,52,53,54,55]. Thioureas, in particular, are structurally versatile ligands due to their simultaneous σ-donating and π-acidic characteristics. The presence of nucleophilic sulphur and nitrogen atoms allows the formation of inter- and intramolecular hydrogen bonds leading to variable binding modes with metal ions, which explains the remarkable coordination abilities exhibited [56,57,58,59]. These properties account for the observed extensive applications established for particular examples such as coordination polymers [60], ligands for gold recovery from electronic wastes [61,62] and small-scale mining [63], chemosensors [64], solar cells [65], catalysts [66,67,68,69] and various bioactivities [70,71,72,73,74,75,76,77].

**Figure 2:**
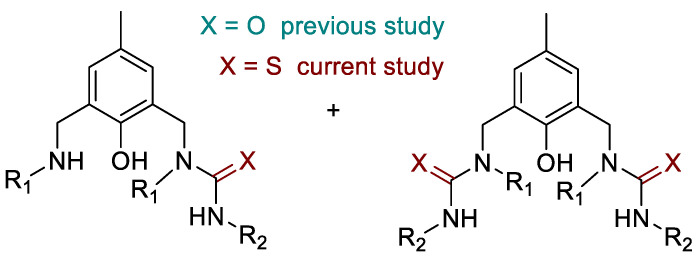
Structures of the ligands, obtained in the previous [78] and in the current studies.

Recently, we reported on the synthesis and coordination ability of a series of polydentate *N*,*O*-ligands possessing unsymmetrical urea fragments attached to a *p*-cresol scaffold [78]. The compounds, obtained via a common experiment, showed negligible interaction with the particular metal ions tested with only a few exceptions. Bearing in mind the specific coordination properties of sulphur-containing molecules, we logically focus our further attention towards the thio-analogues of these products (Figure 2). Herein, we present the synthesis, solution and solid state characterization, and a preliminary study on the coordination properties of a series of novel thiourea-containing ligands.

## 2. Results and Discussion

**Scheme 1:**
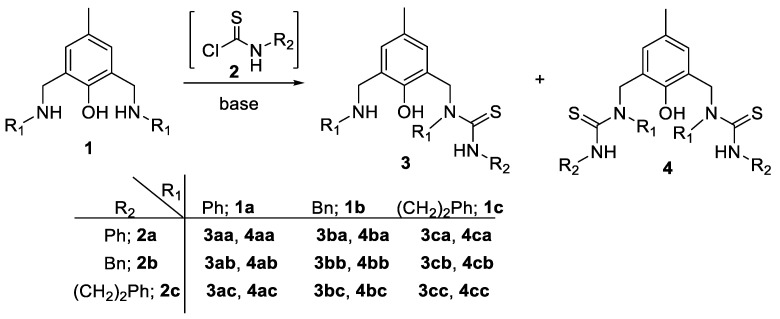
Synthesis of ligands **3** and **4**.

The target ligands containing unsymmetrical thiourea fragments attached to *p*-cresol scaffolds **3** and **4**, shown on Scheme 1, were obtained as separable by chromatography mixtures via a two-step procedure. Initially, thiocarbamic chlorides (**2**) were prepared from primary amines and thiophosgene. The reagent proportions were varied, and the best results were achieved by using equimolar ratios. The freshly prepared thiocarbamic chlorides **2** were subsequently submitted without preliminary purification to reaction with bis-amines **1**, obtained from commercially available 2-hydroxy-5-methyl-1,3-benzenedicarboxaldehyde via reported protocol [78]. The reactions were carried out in benzene at room temperature, the best conditions found for the synthesis of oxygen analogues [78]. The same three types of *N*-substituents were chosen as follows: phenyl, benzyl and phenethyl, that is, aniline, benzyl amine and phenethyl amine, were used to prepare both bis-amines **1** and thiocarbamic chlorides **2**. The proportions of the latter were varied in order to tune the products **3** and **4** ratio. The results are summarized in Table 1.

It is clear from the data listed in Table 1 that the transformation does not proceed smoothly. The target products are isolated in low to moderate overall yields from the reaction mixtures, up to 66%. As expected, monoacylated compounds **3** are prevailing in general when the reagents are used in equimolar ratios and bi-substituted by using excess of the corresponding thiocarbamic chloride **2**.

The results show that the reaction output is strongly dependent on the type of both amine **1** and thiocarbamic chloride **2** substituents, R_1_ and R_2_, respectively, like in the *O*-analogues [78]. Commensurable total yields were achieved from bis-amines **1a** and **1b**, while those from **1c** were generally lower, the yield of **4** being predominant in all cases except **3bb/4bb** couple (entries 13 vs. 15). When performing the transformation with thiocarbamic chloride **2a**, the monoacylated compounds **3aa** (entries 1–3) and **3ca** (entries 19–21) were not detected even when using the equimolar ratio of the reagents, while **3ba** (entries 10,11) was isolated in low yields, up to 32%. At the same time, symmetrical bis-thioureas **4** were obtained in relatively good yields as follows: 66% **4aa** (entry 3), 54% **4ba** (entry 12) and 48% **4ca** (entry 21). The reactions with thiocarbamic chlorides **2b** and **2c** do not follow a particular pattern. The best yields of ligands **3** and **4** were obtained from **1b** with **2b** and **2c**, respectively, 59% **3bb** (entry 13) and 62% **4bc** (entry 17), while the lower conversions were achieved with **2b** from **1c**, 25% **3cb** and 19% **4cb** (entries 22–24), and with **2c** from **1a**, 24% **3ac** and 23% **4ac** (entries 7–9).

**Figure 3:**
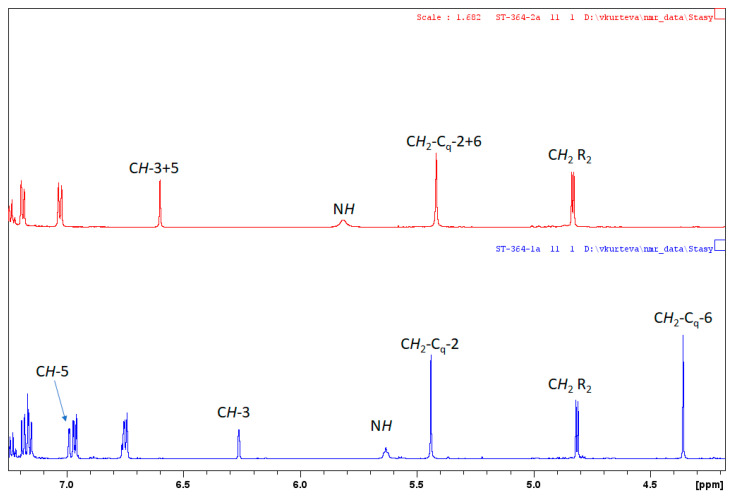
^1^H NMR spectra of products **3ab** (blue) and **4ab** (red).

The structures of the ligands are assigned by 1D and 2D NMR spectra and confirmed by single crystal XRD of selected samples. The NMR spectra show the expected pattern, that is, separate signals for methylene groups and *p*-cresol CH in the monoacylated compounds **3** and common signals in the symmetrical molecules **4**. The differences are the most noticeable for CH protons of *p*-cresol scaffold CH-3 and CH-5 and those for methylene groups directly attached to it CH_2_-C_q_-2 and CH_2_-C_q_-6, which is illustrated in Figure 3 for ligands **3ab** and **4ab**. It has to be noted that the proton spectra of all ligands obtained from **1b** and **1c** show slow exchange between two sites, and the signals for the methylene group neighbour to C_q_-2, in some cases that to C_q_-6 as well, appear as broad singlets. This broadening is very significant in the most examples and leads to the absence of the corresponding signals in the carbon spectra and cross-peaks in 2D correlations (Table 2).

As seen, the chemical shifts of these signals in the proton spectra are also dependent on the substitution pattern, while those in the carbon spectra possess similar values in general. The spectra of mono-substituted products **3** show that the signals for CH protons at the acylated side (C*H*-3) of the *p*-cresol scaffold are shifted significantly upfield and downfield towards C*H*-5 in **3ab** and **3ac** (Figure S1a), respectively; those in compounds obtained from **1b** possess very similar values, while in **3cb** and **3cc** (Figure S1b), the signals for C*H*-3 appear in a slightly stronger field than that for C*H*-5. The signals for the bridged methylene groups at the acylated side (C*H_2_*-C_q_-2) are shifted downfield in respect to that for C*H*_2_-C_q_-6 in the spectra of **3ab**, **3ac** (Figure S1c), **3ba** and **3bb** and upfield in those of the rest of the products (Figure S1d; for **3cb** and **3cc**). At the same time, the signals for both CH-3+5 and CH_2_-C_q_-2+6 in the spectra of ligands **4** are shifted up-field and down-field, respectively, ordered from phenyl to phenethyl independently on the substituent in **2**. The latter is illustrated on the example of ligands **4ab**, **4bb** and **4cb** spectra in Figure S2. Based on these observations, it can be stated that the substituents on both bis-amines **1** and thiocarbamic chlorides **2** influence the chemical shifts of the products.

**Figure 4:**
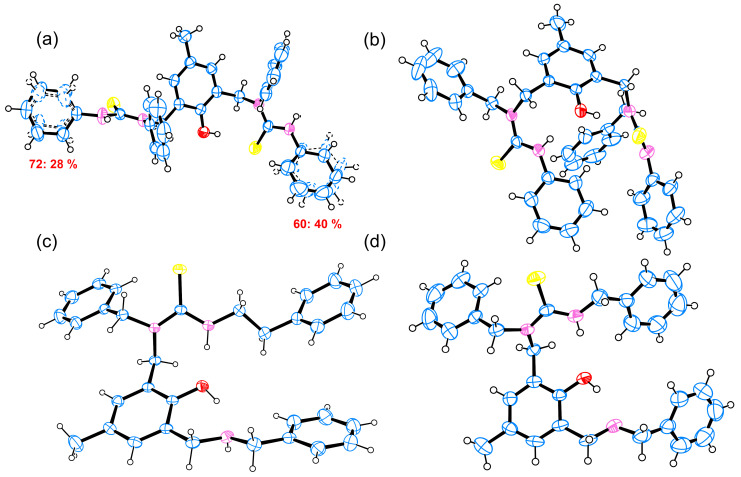
ORTEP view of the products: (**a**) **4aa**, (**b**) **4ba**, (**c**) **3bc** and (**d**) **3bb**.

**Figure 5:**
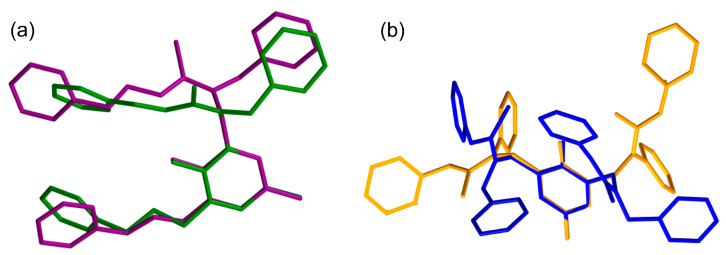
Overlay of the molecules of (**a**) **3bb** (green) and **3bc** (magenta) disclosing the similar orientation of the side chains and (**b**) **4aa** (orange) and **4ba** (blue) disclosing the dissimilar orientation of the side chains.

Appropriate for single crystal XRD phases are grown from ligands **3bb**, **3bc**, **4aa** and **4ba**. The ORTEP views are shown in Figure 4. The mono-acylated bis-amines **3bb** and **3bc** crystallize in monoclinic and triclinic crystal systems in space groups *Pc* and *P*–1, respectively. In the crystal structure of **3bc**, two molecules are present in the asymmetric unit. The overlay of the two molecules provides a rmsd of 0.1961 Å (Figure S3) and discloses that molecular geometry is highly conserved. The bond lengths and bond angles in **3bb** (Tables S2 and S6) and **3bc** (Tables S3 and S7) are comparable and similar to those observed in related compounds [78]. In both compounds, the aromatic moieties are nearly planar with rmsd of the order of 0.025 Å. The unsymmetrical urea moieties in **3bb** and **3bc** exhibit a conserved molecular geometry. This conservation likely arises due to the specific electronic and steric properties of the unsymmetrical urea group, which imposes structural constraints, maintaining a consistent shape across **3bb** and **3bc** molecules (Figure 5). These conserved geometrical features can result in predictable interactions and coordination patterns, which are valuable in the design and application of such ligands. The crystal structures of the symmetrical bis-thioureas **4aa** and **4ba** (Figure 4a,b) crystallize in the triclinic system, space group *P*–1. The bond lengths and dihedral angles of the molecules present in the asymmetric units of **4aa** (Tables S4 and S8) and **4ba** (Tables S5 and S9) are comparable. In contrast to **3bb** and **3bc** molecules, the side chains in **4aa** and **4ba** are more flexible. This flexibility results in variations in the overall molecular geometry of the ligands and differences in the distances between the supposed coordination centres. Such variations are indicative of less rigid structural elements, possibly due to different substituents or chain lengths that allow for conformational adjustments. The observed positional/rotational disorder of the phenyl moieties (Figure 4a) observed in **4aa** also supports the reduced rigidity of the molecules.

The *p*-cresol scaffold in **3bb, 3bc** and **4ba** participates in two intramolecular hydrogen bonding interactions (Tables S10–S12), stabilizing the molecular geometry. In **3bc** and **3bb**, the two intramolecular hydrogen bonds are of O-H…N and N-H…O type, while for **4ba**, the two intramolecular interactions are O-H…S and N-H…O. In **4aa**, only one intramolecular interaction of O-H…S type is observed (Table S13).

**Figure 6:**
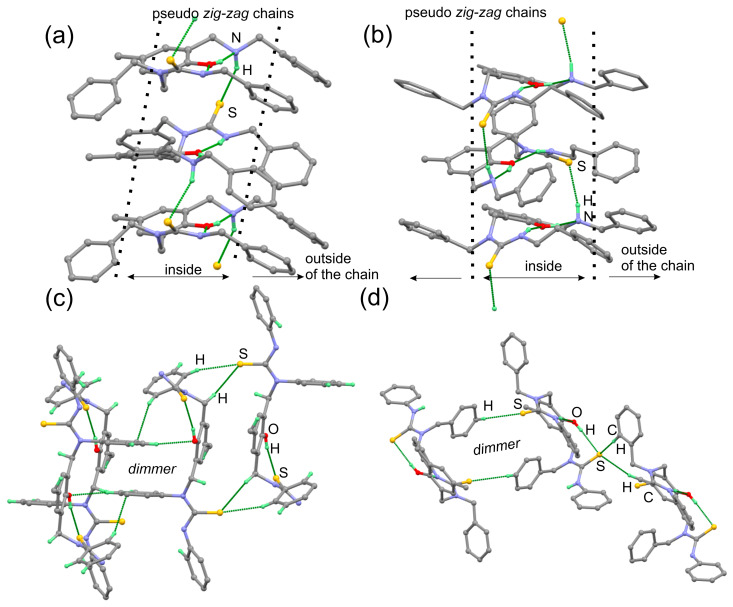
Representation of the N-H…S interactions (as green doted lines) observed in compounds **3bb** (**a**), **3bc** (**b**), **4aa** (**c**) and **4ba** (**d**).

In **3bb** and **3bc**, the combination of the intramolecular interaction and the intermolecular N-H…S interaction forms the core of infinite zigzag chains (Figure 6a,b). The outside of the zigzag chains is decorated mostly by the aromatic rings. In **4aa** and **4ba**, although two distinct S and N-H centres are present, no N-H…S interaction is detected (Figure 6c,d).

**Figure 7:**
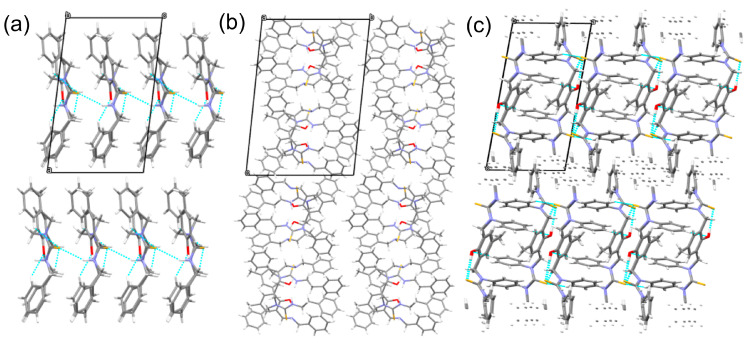
Visualization of the three-dimensional arrangement of the molecules producing pseudo-layers (**a**) compound **3bb**, (**b**) compound **3bc** and (**c**) compound **4aa**.

The obvious difference when comparing **4aa** and **4ba** interactions with those of compounds **3bb** and **3bc** is that the intramolecular O-H…N interaction is replaced by an O-H…S interaction or vice versa. The “second S” in **4aa** and **4ba** centre is involved in C-H…S interactions between adjacent molecules producing dimmers. The three-dimensional packing of the molecules is governed by the presence of several aromatic moieties, decorating the outside of the chains. Those aromatic moieties tend to stabilize the packing of the molecules in the crystal structure through π…π interactions (Table 3). In **3bb**, **3bc** and **4ba** (Figures S4–S6), the π…π interactions can be related to the *T-shape* type, while in **4aa** the π…π interaction is closer to the parallel displaced stacking type (Figure S7). This arrangement produces pseudo-layers with the aromatic moieties decorating the outside of the layers (Figure 7).

The N-H…O and O-H…S intramolecular interactions contribute to the stabilization of the molecular geometry by forming a compact structure, but they also increase the NH group’s acidity because the proton is more easily donated. This is the case in **3bb** and **3bc**, where the one NH group participates in an intramolecular N-H…O hydrogen bond and the second NH group participates in an intermolecular N-H…S hydrogen bond. These hydrogen bonding interactions weaken the N-H bond by pulling electron density toward the oxygen/sulphur atom, making the NH groups more prone to losing their proton. Thus, the NH groups will become more acidic (due to the stabilization provided by the hydrogen bond with the electronegative atom). In **4ba**, the NH group is also relatively acidic due to the presence of an intramolecular N-H…O hydrogen bond, although it lacks the additional stabilization provided by an intermolecular N-H…S interaction. In **4aa**, the NH group is less acidic compared to **3bb**, **3bc** and **4ba**, as it only participates in one intramolecular hydrogen bond, O-H…S, and lacks both N-H…O and N-H…S interactions. Summarizing the effect of the hydrogen bonding interaction on the NH groups, the suggestion is that in **3bb** and **3bc**, the NH groups are more acidic, slightly less acidic in **4ba**, and the NH group is the least acidic in **4aa**, as it lacks both the N-H…O and N-H…S interactions.

**Figure 8:**
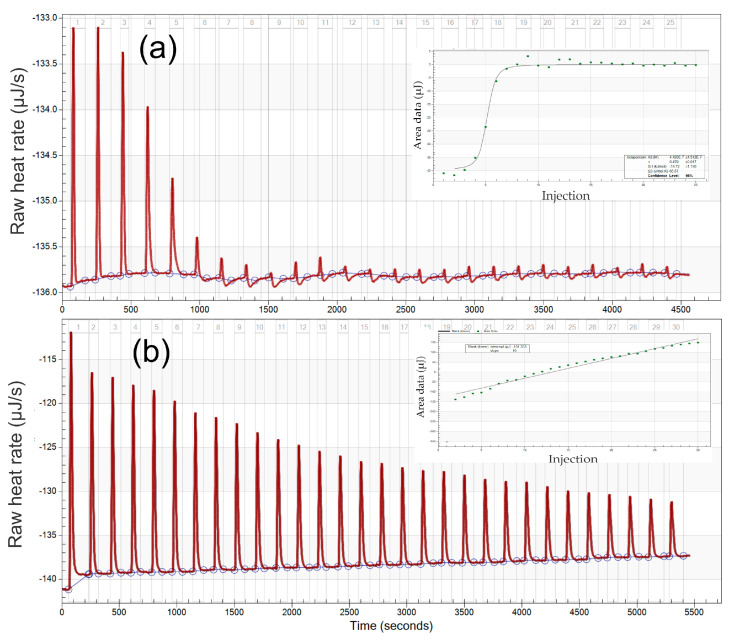
Typical calorimetric titration isotherms of: (**a**) the binding interaction between **3ba** and K^+^ (the inset shows the fitted association constant) and (**b**) isotherm showing no interaction between **3ba** and Ca^2+^ (the inset shows the blank model (linear) attempted to reproduce the interaction).

Finally, a preliminary investigation into the coordination abilities of the synthesized in reasonable yields products was conducted using isothermal titration calorimetry (ITC). This method is highly sensitive and effective for providing insights into complexation reactions, thus allowing the evaluation of the strength of metal–ligand interaction. In this study, ITC was utilized to examine the interactions of caesium (I), potassium (I), calcium (II) and zinc (II) ions with the synthesized ligands within a near-neutral pH range of 6.5 to 7.5. This pH range was selected to maintain environmentally friendly conditions. A typical representation of ITC data illustrating both an interaction and a lack of interaction is shown in Figure 8.

Our initial intent was to employ phosphate buffer and to adjust the pH to acidic, neutral and basic. However, during the initial test ligand vs. buffer, an interaction with the buffer was recognized. Consequently, the interaction was investigated using ligand and ultrapure water (no pH adjustment) and sodium and potassium ions. While no significant interaction was registered between the ligands and Na, for some of the ligands, *K*a with potassium ions was substantial. Subsequently, we performed additional experiments in the same conditions with Cs^+^, Ca^2+^ and Zn^2+^. The ITC data revealed that probably the selected compounds do not interact with the M (II) ions. Interestingly, while compounds interact with K^+^ (Table 4), only **4aa**, **3ab** and **3ac** interact with Cs^+^ for the selected conditions, the latter being weaker than those with K^+^. Having in mind the intramolecular hydrogen bond detected in all crystal structures, one may suppose that the metal–ligand interaction needs to be both specific and selective in order to be energetically favourable. This suggestion outlines the direction of the further studies towards inactivating the intramolecular interactions of the compounds and opening the NH centres to a larger panel of metal ions.

The ITC data disclose that all ligands interact substantially with potassium ions; only a part display weaker interactions with caesium, while no compound interacts with the rest of the metal ions tested in the particular conditions.

## 3. Materials and Methods

### 3.1. General

All reagents are purchased from Aldrich, Merck and Fluka and are used without any further purification. The deuterated chloroform is purchased from Deutero GmbH. Fluka silica gel (TLC-cards 60,778 with fluorescent indicator 254 nm) is used for TLC chromatography and R_f_-value determination. Merck Silica gel 60 (0.040–0.063 mm) is used for flash chromatography purification of the products. The melting points are determined in capillary tubes on the SRS MPA100 OptiMelt (Sunnyvale, CA, USA) automated melting point system with a heating rate of 1 °C per min. The NMR spectra are recorded on the Bruker Avance NEO 400 spectrometer (Rheinstetten, Germany) in CDCl_3_; the chemical shifts are quoted in ppm in δ-values against tetramethylsilane (TMS) as an internal standard, and the coupling constants are calculated in Hz. The assignment of the signals is confirmed by applying two-dimensional NOESY, HSQC and HMBC techniques. The spectra are processed with the Topspin 3.6.3 program. The mass spectra were recorded in positive mode on Q Exactive Plus Hybrid Quadrupole-Orbitrap Mass Spectrometer Thermo Scientific (HESI HRMS). The spectra were processed with Xcalibur Free Style program version 4.5 (Thermo Fisher Scientific Inc., Waltham, MA, USA).

### 3.2. General Procedure for the Preparation of Ligands **3** and **4**

*Step 1:* To a solution of thiophosgene (15 mmol) in benzene (35 mL), a primary amine (15 mmol) was added, and the mixture was stirred at room temperature for 1 h in an argon atmosphere. The products were partitioned between benzene and water. The organic layer was dried over MgSO_4_ and evaporated to dryness. The crude reagents **2** were isolated in 30–37% yield and were further used without purification in order to avoid decomposition.

**Figure 9:**
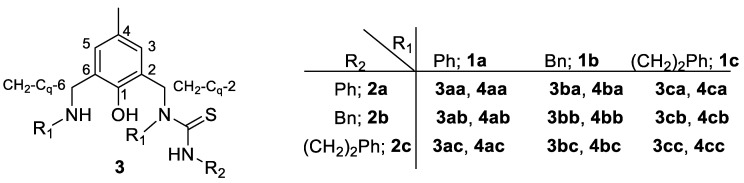
Structure and numeration scheme of ligands **3**; ligands **4** are symmetrical.

*Step 2:* To a solution of bis-amine **1** (1 mmol) and pyridine (1–3 mmol) in benzene (10 mL), freshly prepared thiocarbamic chloride **2** (1–3 mmol) was added portion-wise, and the mixture was stirred at room temperature for 24 h in an argon atmosphere. The solution was extracted by brine, washed with 10% aq. HCl and then with brine, dried over MgSO_4_, and evaporated to dryness. The products were separated by flash chromatography on silica gel by using a mobile phase with gradient of polarity from DCM to 2% MeOH/DCM. The reagents’ ratios and the reaction yields are summarized in Table 1. The numeration scheme of the ligands **3** and **4** is given on Figure 9.

*Ligand **4aa**:* R_f_ 0.30 (DCM); colourless solid; m. p. 138.3–139.3 °C; ^1^H NMR 2.033 (s, 3H, C*H*_3_), 5.472 (s, 4H, C*H*_2_-C_q_-2 and C*H*_2_-C_q_-6), 6.622 (s, 2H, C*H*-3 and C*H*-5), 7.134 (dd, 4H, J 7.7, 1.2, C*H*-Ph-o of R_1_), 7.192 (tt, 2H, J 7.1, 1.4, C*H*-Ph-p of R_2_), 7.300–7.350 (m, 8H, C*H*-Ph-o of R_2_ and C*H*-Ph-m of R_2_), 7.389 (tt, 2H, J 7.4, 1.1, C*H*-Ph-p of R_1_), 7.444 (ddt, 4H, J 7.8, 7.3, 1.0, C*H*-Ph-m of R_1_), 8.814 (bs, 1H, O*H*); ^13^C NMR 20.37 (*C*H_3_), 54.36 (C*H*_2_-C_q_-2 and C*H*_2_-C_q_-6), 122.19 (*C*_q_-2 and *C*_q_-6), 125.86 (*C*H Ph-o of R_2_), 126.25 (*C*H Ph-p of R_2_), 128.16 (*C*_q_-4), 128.44 (*C*H Ph-o of R_1_), 128.65 (*C*H Ph-m of R_2_), 129.01 (*C*H Ph-p of R_1_), 130.55 (*C*H Ph-m of R_1_), 131.69 (*C*H-3 and *C*H-5), 139.05 (*C*_q_-1 Ph of R_2_), 141.15 (*C*_q_-1 Ph of R_1_), 151.18 (*C*_q_-1), 181.21 (*C*=S); HRMS (HESI^+^) *m*/*z* calcd. for C_35_H_33_N_4_OS_2_^+^ [M + H]^+^ 589.2090, found 589.2089, ∆ = −0.1 mDa.

*Ligand **3ab**:* R_f_ 0.56 (1% MeOH/DCM); colourless oil; ^1^H NMR 2.041 (s, 3H, C*H*_3_), 4.360 (s, 2H, C*H*_2_-C_q_-6), 4.815 (d, 2H, J 5.4, C*H*_2_ of R_2_), 5.441 (s, 2H, C*H*_2_-C_q_-2), 5.633 (bt, 1H, J 5.1, N*H*-CS), 6.263 (d, 1H, J 1.9, C*H*-3), 6.752 (m, 3H, C*H*-Ph-o and C*H*-Ph-p of NH-R_1_), 6.965 (m. 2H, C*H*-Ph-o of R_2_), 6.991 (d, 1H, J 1.9, C*H*-5), 7.151–7.194 (m, 4H, C*H*-Ph-o of N-R_1_ and C*H*-Ph-m of NH-R_1_), 7.231 (tt, 1H, J 7.3, 1.4, C*H*-Ph-p of R_2_), 7.280 (ddt, 2H, J 7.4, 7.0, 1.4, C*H*-Ph-m of N-R_1_), 7.342–7.382 (m, 3H, C*H*-Ph-m of R_2_ and C*H*-Ph-p of N-R_1_), 8.827 (bs, 1H, O*H*); ^13^C NMR 20.31 (*C*H_3_), 45.29 (C*H*_2_-C_q_-6), 49.99 (*C*H_2_ of R_2_), 55.67 (C*H*_2_-C_q_-2), 114.24 (*C*H Ph-o of NH-R_1_), 118.35 (*C*H Ph-p of NH-R_1_), 121.25 (*C*_q_-2), 125.69 (*C*_q_-6), 127.27 (*C*H Ph-o of N-R_1_), 127.58 (*C*H Ph-p of R_2_), 128.09 (*C*_q_-4), 128.48 (*C*H Ph-o of R_2_), 128.71 (*C*H Ph-m of N-R_1_), 129.17 (*C*H Ph-m of NH-R_1_), 129.26 (*C*H Ph-p of N-R_1_), 130.09 (*C*H-5), 130.53 (*C*H Ph-m of R_2_), 131.38 (*C*H-3), 137.53 (*C*_q_-1 Ph of N-R_1_), 139.63 (*C*_q_-1 Ph of R_2_), 147.78 (*C*_q_-1 Ph of NH-R_1_), 151.82 (*C*_q_-1), 181.17 (*C*=S); HRMS (HESI^+^) *m*/*z* calcd. for C_29_H_30_N_3_OS^+^ [M + H]^+^ 468.2104, found 468.2103, ∆ = −0.1 mDa.

*Ligand **4ab**:* R_f_ 0.34 (DCM); colourless oil; ^1^H NMR 2.027 (s, 3H, C*H*_3_), 4.833 (d, 4H, J 5.3, C*H*_2_ of R_2_), 5.418 (s, 4H, C*H*_2_-C_q_-2 and C*H*_2_-C_q_-6), 5.816 (bt, 2H, N*H*-CS), 6.601 (s, 2H, C*H*-3 and C*H*-5), 7.029 (dd, 4H, J 7.4, 1.3, C*H*-Ph-o of R_1_), 7.190 (dd, 4H, J 7.2, 1.3, C*H*-Ph-o of R_2_), 7.235 (tt, 2H, J 7.3, 1.2, C*H*-Ph-p of R_2_), 7.289 (ddt, 4H, J 7.6, 7.1, 1.3, C*H*-Ph-m of R_2_), 7.315 (tt, 2H, J 7.3, 1.2, C*H*-Ph-p of R_1_), 7.365 (ddt, 4H, J 7.8, 7.2, 1.2, C*H*-Ph-m of R_1_), 8.642 (bs, 1H, O*H*); ^13^C NMR 20.40 (*C*H_3_), 49.99 (*C*H_2_ of R_2_), 54.45 (C*H*_2_-C_q_-2 and C*H*_2_-C_q_-6), 122.40 (*C*_q_-2 and *C*_q_-6), 127.35 (*C*H Ph-o of R_2_), 127.49 (*C*H Ph-p of R_2_), 127.93 (*C*_q_-4), 128.43 (*C*H Ph-o of R_1_), 128.67 (*C*H Ph-m of R_2_), 128.81 (*C*H Ph-p of R_1_), 130.25 (*C*H Ph-m of R_1_), 131.16 (*C*H-3 and *C*H-5), 137.77 (*C*_q_-1 Ph of R_2_), 140.73 (*C*_q_-1 Ph of R_1_), 151.13 (*C*_q_-1), 181.80 (*C*=S); HRMS (HESI^+^) *m*/*z* calcd. for C_37_H_37_N_4_OS_2_^+^ [M + H]^+^ 617.2403, found 617.2400, ∆ = −0.3 mDa.

*Ligand **3ac**:* R_f_ 0.52 (1% MeOH/DCM); colourless solid; m. p. 126.0–127.0 °C; ^1^H NMR 2.019 (s, 3H, C*H*_3_), 2.785 (t, 2H, J 6.7, C*H*_2_-Ph of R_2_), 3.781 (ddd, 2H, J 12.1, 6.7, 5.3, C*H*_2_-NH of R_2_), 4.344 (s, 2H, C*H*_2_-C_q_-6), 5.265 (bt, 1H, J 5.1, N*H*-CS), 5.365 (s, 2H, C*H*_2_-C_q_-2), 6.210 (d, 1H, J 1.9, C*H*-5), 6.732 (m, 3H, C*H*-Ph-o and C*H*-Ph-p of NH-R_1_), 6.772 (m, 2H, C*H*-Ph-o of N-R_1_), 6.948 (dd, 2H, J 7.7, 2.2, Ph-o of R_2_), 6.978 (d, 1H, J 1.7, C*H*-3), 7.142–7.180 (m, 5H, C*H*-Ph-m and C*H*-Ph-p of NH-R_1_ and C*H*-Ph-m of R_2_), 7.291 (tt, 2H, J 7.6, 1.3, C*H*-Ph-m of N-R_1_), 7.332 (tt, 1H, J 7.3, 1.3, C*H*-Ph-p of N-R_1_), 8.786 (bs, 1H, O*H*); ^13^C NMR 20.31 (*C*H_3_), 34.83 (*C*H_2_-Ph of R_2_), 45.06 (C*H*_2_-C_q_-6), 46.97 (*C*H_2_-NH of R_2_), 55.31 (C*H*_2_-C_q_-2), 114.07 (*C*H Ph-o of NH-R_1_), 118.16 (*C*H Ph-o of NH-R_1_), 121.28 (*C*_q_-2), 125.78 (*C*_q_-6), 126.49 (*C*H Ph-p of R_2_), 127.99 (*C*_q_-4), 128.43 (*C*H Ph-o of N-R_1_), 128.59 (*C*H Ph-o of R_2_), 128.70 (*C*H Ph-m), 129.07 (*C*H Ph-p of N-R_1_), 129.17 (*C*H Ph-m), 130.01 (*C*H-3), 130.40 (*C*H Ph-m of N-R_1_), 131.37 (*C*H-5), 138.33 (*C*_q_-1 Ph of R_2_), 139.41 (*C*_q_-1 Ph of N-R_1_), 147.92 (*C*_q_-1 Ph of NH-R_1_), 151.80 (*C*_q_-1), 180.56 (*C*=S); HRMS (HESI^+^) *m*/*z* calcd. for C_30_H_32_N_3_OS^+^ [M + H]^+^ 482.2261, found 482.2259, ∆ = −0.2 mDa.

*Ligand **4ac**:* R_f_ 0.28 (DCM); colourless oil; ^1^H NMR 2.012 (s, 3H, C*H*_3_), 2.804 (t, 4H, J 6.8, C*H*_2_-Ph of R_2_), 3.795 (ddd, 4H, J 12.1, 6.8, 5.5, C*H*_2_-NH of R_2_), 5.349 (s, 4H, C*H*_2_-C_q_-2 and C*H*_2_-C_q_-6), 5.447 (bs, 2H, N*H*-CS), 6.559 (s, 2H, C*H*-3 and C*H*-5), 6.837 (m, 4H, C*H*-Ph), 6.986 (dd, 4H, J 7.4, 1.6, C*H*-Ph-o of R_2_), 7.150–7.193 (m, 6H, C*H*-Ph), 7.267–7.296 (m, 6H, C*H*-Ph), 8.607 (bs, 1H, O*H*); ^13^C NMR 20.42 (*C*H_3_), 34.85 (*C*H_2_-Ph of R_2_), 46.95 (*C*H_2_-NH of R_2_), 54.05 (C*H*_2_-C_q_-2 and C*H*_2_-C_q_-6), 122.44 (*C*_q_-2 and *C*_q_-6), 126.42 (*C*H Ph-p), 127.79 (*C*_q_-4), 128.35 (*C*H Ph), 128.62 (*C*H Ph-p), 128.63 (*C*H Ph), 128.65 (*C*H Ph), 130.13 (*C*H Ph), 130.95 (*C*H-3 and *C*H-5), 138.54 (*C*_q_-1 Ph of R_2_), 140.44 (*C*_q_-1 Ph of R_1_), 151.09 (*C*_q_-1), 181.24 (*C*=S); HRMS (HESI^+^) *m*/*z* calcd. for C_39_H_41_N_4_OS_2_^+^ [M + H]^+^ 645.2716, found 645.2715, ∆ = −0.1 mDa.

*Ligand **3ba**:* R_f_ 0.30 (5% MeOH/DCM); colourless solid; m. p. 144.3–144.9 °C; ^1^H NMR 2.237 (s, 3H, C*H*_3_), 3.819 (s, 2H, C*H*_2_-NH of R_1_), 4.005 (s, 2H, C*H*_2_-C_q_-6), 4.645 (bs, 2H, C*H*_2_-C_q_-2), 5.293 (bs, 2H, C*H*_2_-N of R_1_), 6.830 (s, 1H, C*H*-5), 6.850 (s, 1H, C*H*-3), 7.147 (tt, 2H, J 7.4, 1.1, C*H* Ph), 7.266–7.408 (m, 12H, C*H* Ph), 7.473 (bd, 2H, J 7.5, C*H* Ph-o), 9.475 (bs, 1H, O*H*); ^13^C NMR 20.54 (*C*H_3_), 51.35 (C*H*_2_-C_q_-6), 52.42 (*C*H_2_-NH of R_1_), 53.95 (*C*H_2_-N of R_1_), 121.89 (*C*_q_-2 and *C*_q_-6), 124.88 (*C*H Ph), 124.99 (*C*H Ph), 127.49 (*C*H Ph), 127.91 (*C*H Ph), 128.01 (*C*H Ph), 128.49 (*C*H Ph), 128.66 (*C*_q_-4), 128.75 (*C*H Ph), 128.93 (*C*H Ph), 129.04 (*C*H Ph), 129.68 (*C*H-5), 131.15 (*C*H-3), 136.85 (*C*_q_-1 Ph of N-R_1_), 137.14 (*C*_q_-1 Ph of NH-R_1_), 141.14 (*C*_q_-1 Ph of R_2_), 153.21 (*C*_q_-1), 183.14 (*C*=S); HRMS (HESI^+^) *m*/*z* calcd. for C_30_H_32_N_3_OS^+^ [M + H]^+^ 482.2261, found 482.2260, ∆ = −0.1 mDa.

*Ligand **4ba**:* R_f_ 0.56 (DCM); colourless solid; m. p. 155.9–157.3 °C; ^1^H NMR 2.268 (s, 3H, C*H*_3_), 5.017 (bs, 8H, 2C*H*_2_-Ph of R_1_, C*H*_2_-C_q_-2 and C*H*_2_-C_q_-6), 6.941 (s, 2H, C*H*-3 and C*H*-5), 7.178 (bs, 3H, C*H* Ph), 7.284 (bm, 6H, C*H* Ph), 7.355 (bm, 6H, C*H* Ph), 7.439 (bm, 5H, C*H* Ph), 10.017 (bs, 1H, O*H*); ^13^C NMR 20.39 (*C*H_3_), 123.18 (*C*_q_-2 and *C*_q_-6), 125.54 (*C*H Ph), 125.75 (*C*H Ph), 126.95 (*C*_q_-4), 128.56 (*C*H Ph), 128.64 (*C*H Ph), 129.10 (*C*H Ph), 129.38 (*C*H Ph), 129.55 (*C*H Ph), 133.00 (*C*H-3 and *C*H-5), 139.54 (*C*_q_-1 Ph), 151.52 (*C*_q_-1), 182.49 (*C*=S); HRMS (HESI^+^) *m*/*z* calcd. for C_37_H_37_N_4_OS_2_^+^ [M + H]^+^ 617.2403, found 617.2402, ∆ = −0.1 mDa.

*Ligand **3bb**:* R_f_ 0.32 (5% MeOH/DCM); colourless solid; m. p. 89.2–90.3 °C; ^1^H NMR 2.187 (s, 3H, C*H*_3_), 3.656 (s, 2H, C*H*_2_-NH of R_1_), 3.785 (s, 2H, C*H*_2_-C_q_-6), 4.518 (bs, 2H, C*H*_2_-C_q_-2), 4.908 (d, 2H, J 4.9, C*H*_2_-NH of R_2_), 5.384 (s, 2H, C*H*_2_-N of R_1_), 6.713 (s, 1H, C*H*-5), 6.770 (s, 1H, C*H*-3), 7.124 (m, 2H, C*H* Ph), 7.192 (m, 5H, C*H* Ph), 7.293–7.376 (m, 6H, C*H* Ph), 7.421 (bd, 2H, J 7.0, C*H* Ph-o); ^13^C NMR 20.52 (*C*H_3_), 50.57 (*C*H_2_-of R_2_), 51.24 (C*H*_2_-C_q_-6), 52.36 (*C*H_2_-NH of R_1_), 55.77 (*C*H_2_-N of R_1_), 121.97 (*C*_q_-2 and *C*_q_-6), 127.05 (*C*H Ph), 127.29 (*C*H Ph), 127.47 (*C*H Ph), 127.67 (*C*H Ph), 127.85 (*C*H Ph), 128.39 (*C*H Ph), 128.42 (*C*_q_-4), 128.72 (*C*H Ph),128.80 (*C*H Ph), 129.26 (*C*H-5), 129.88 (*C*H-3), 138.17 (*C*_q_-1 Ph), 138.40 (*C*_q_-1 Ph), 151.80 (*C*_q_-1), 182.63 (*C*=S); HRMS (HESI^+^) *m*/*z* calcd. for C_31_H_34_N_3_OS^+^ [M + H]^+^ 496.2417, found 496.2415, ∆ = −0.2 mDa.

*Ligand **4bb**:* R_f_ 0.48 (DCM); colourless oil; ^1^H NMR 2.185 (s, 3H, C*H*_3_), 4.788 (d, 4H, C*H*_2_ of R_2_), 4.893 (bs, 6H, C*H*_2_), 6.388 (bs, 2H, N*H*-CS), 6.824 (s, 2H, C*H*-3 and C*H*-5), 7.076 (bs, 5H, C*H* Ph), 7.209–7.224 (m, 9H, C*H* Ph), 7.301 (tt, 2H, J 7.3, 1.3, C*H* Ph-p), 7.347 (tt, 4H, J 7.1, 1.5, C*H* Ph-m), 9.385 (bs, 1H, O*H*); ^13^C NMR 20.41 (*C*H_3_), 50.55 (*C*H_2_-of R_2_), 121.88 (*C*_q_-2 and *C*_q_-6), 126.86 (*C*H Ph), 127.50 (*C*H Ph),127.63 (*C*H Ph), 127.96 (*C*H Ph-p), 128.66 (*C*H Ph), 129.12 (*C*_q_-4), 129.17 (*C*H Ph-m), 131.77 (*C*H-3 and *C*H-5), 137.56 (*C*_q_-1 Ph), 151.39 (*C*_q_-1), 182.06 (*C*=S); HRMS (HESI^+^) *m*/*z* calcd. for C_39_H_41_N_4_OS_2_^+^ [M + H]^+^ 645.2716, found 645.2716, ∆ = 0 mDa.

*Ligand **3bc**:* R_f_ 0.24 (5% MeOH/DCM); colourless solid; m. p. 105.2–105.7 °C; ^1^H NMR 2.195 (s, 3H, C*H*_3_), 2.894 (t, 2H, J 7.1, C*H*_2_-Ph of R_2_), 3.794 (bs, 2H, C*H*_2_-NH of R_1_), 3.906 (ddd, 2H, J 12.1, 7.1, 5.2, C*H*_2_-N of R_2_), 3.928 (s, 2H, C*H*_2_-N of R_1_), 4.497 (bs, 2H, C*H*_2_-C_q_-2), 5.213 (bs, 2H, C*H*_2_-C_q_-6), 6.763 (s, 2H, C*H*-3 and C*H*-5), 7.107 (d, 2H, J 7.4 C*H*-Ph-o), 7.150 (tt, 1H, J 7.3, 1.4 C*H*-Ph-p), 7.198 (tt, 2H, J 7.4, 1.4m C*H*-Ph-m), 7.268–7.311 (m, 4H, C*H*-Ph), 7.331–7.360 (m, 6H, C*H*-Ph); ^13^C NMR 20.54 (*C*H_3_), 35.21 (*C*H_2_-Ph of R_2_), 47.62 (*C*H_2_-NH of R_2_), 51.35 (*C*H_2_-N of R_1_), 52.42 (*C*H_2_-NH of R_1_), 121.86 (*C*_q_-2 and *C*_q_-6), 126.18 (*C*H Ph), 127.40 (*C*H Ph), 127.59 (*C*H Ph), 127.96 (*C*H Ph), 128.45 (*C*H Ph), 128.58 (*C*H Ph), 128.70 (*C*H Ph), 128.73 (*C*_q_-4), 128.76 (*C*H Ph), 128.87 (*C*H Ph), 129.82 (*C*H-3 and *C*H-5), 139.16 (*C*_q_-1 Ph), 152.97 (*C*_q_-1), 182.62 (*C*=S); HRMS (HESI^+^) *m*/*z* calcd. for C_32_H_36_N_3_OS^+^ [M + H]^+^ 510.2574, found 510.2574, ∆ = 0 mDa.

*Ligand **4bc**:* R_f_ 0.42 (DCM); colourless solid; m. p. 152.4–153.2 °C; ^1^H NMR 2.166 (s, 3H, C*H*_3_), 2.799 (bs, 4H, C*H*_2_-Ph of R_2_), 3.828 (bm, 4H, C*H*_2_-NH of R_2_), 4.836 (bs, 8H, C*H*_2_ of R_1_, C*H*_2_-C_q_-2 and C*H*_2_-C_q_-6), 5.946 (bs, 2H, N*H*-CS), 6.776 (s, 2H, C*H*-3 and C*H*-5), 7.046 (bs, 4H, C*H*-Ph), 7.140–7.203 (m, 8H, C*H*-Ph), 7.276–7.343 (m, 8H, C*H*-Ph), 9.585 (bs, 1H, O*H*); ^13^C NMR 20.41 (*C*H_3_), 35.00 (*C*H_2_-Ph of R_2_), 47.55 (*C*H_2_-NH of R_2_), 121.80 (*C*_q_-2 and *C*_q_-6), 126.46 (*C*H Ph), 127.86 (*C*_q_-4), 128.62 (*C*H Ph), 128.64 (*C*H Ph), 129.03 (*C*H Ph), 129.09 (*C*H Ph), 131.75 (*C*H-3 and *C*H-5), 138.51 (*C*_q_-1 Ph), 151.32 (*C*_q_-1), 181.83 (*C*=S); HRMS (HESI^+^) *m*/*z* calcd. for C_41_H_45_N_4_OS_2_^+^ [M + H]^+^ 673.3029, found 673.3030, ∆ = 0.1 mDa.

*Ligand **4ca**:* R_f_ 0.50 (DCM); colourless oil; ^1^H NMR 2.337 (s, 3H, C*H*_3_), 3.041 (bt, 4H, J 6.7, C*H*_2_-Ph of R_1_), 3.951 (bs, 4H, C*H*_2_-N of R_1_), 4.956 (bs, 4H, C*H*_2_-C_q_-2 and C*H*_2_-C_q_-6), 7.027 (s, 2H, C*H*-3 and C*H*-5), 7.131 (bt, 4H, J 7.4, C*H* Ph), 7.229–7.279 (m, 10H, C*H* Ph), 7.367 (btd, 4H, J 7.2, 1.0, C*H* Ph), 9.969 (bs, 1H, O*H*); ^13^C NMR 20.49 (*C*H_3_), 33.10 (*C*H_2_-Ph of R_1_), 122.63 (*C*_q_-2 and *C*_q_-6), 125.30 (*C*H Ph), 125.76 (*C*H Ph), 128.53 (*C*H Ph), 128.84 (*C*_q_-4), 128.93 (*C*H Ph), 129.24 (*C*H Ph), 129.32 (*C*H Ph), 132.78 (*C*H-3 and *C*H-5), 138.74 (*C*_q_-1 Ph), 139.75 (*C*_q_-1 Ph), 151.43 (*C*_q_-1), 182.00 (*C*=S); HRMS (HESI^+^) *m*/*z* calcd. for C_39_H_41_N_4_OS_2_^+^ [M + H]^+^ 645.2716, found 645.2719, ∆ = 0.3 mDa.

*Ligand **3cb**:* R_f_ 0.28 (5% MeOH/DCM); colourless oil; ^1^H NMR 2.197 (s, 3H, C*H*_3_), 2.732 (bt, 2H, J 6.4, C*H*_2_-Ph of NR_1_), 2.820 (bt, 2H, J 6.4, C*H*_2_-N of NR_1_), 3.090 (bt, 2H, J 7.5, C*H*_2_-Ph of NHR_1_), 3.805 (s, 2H, C*H*_2_-C_q_-2), 4.187 (bs, 2H, C*H*_2_-C_q_-6), 4.484 (bs, 2H, C*H*_2_-N of NHR_1_), 4.855 (d, 4H, J 4.9, C*H*_2_ of R_2_), 5.368 (bs, 1H, N*H*-CS), 6.725 (s, 1H, C*H*-3), 6.895 (d, 1H, J 1.7, C*H*-5), 7.117–7.247 (m, 9H, C*H* Ph), 7.282–7.318 (m, 6H, C*H* Ph), 9.613 (bs, 1H, O*H*); ^13^C NMR 20.48 (*C*H_3_), 33.70 (*C*H_2_-Ph of NHR_1_), 35.02 (*C*H_2_-Ph of NR_1_), 48.19 (*C*H_2_-N of NHR_1_), 49.21 (*C*H_2_-N of NR_1_), 50.21 (*C*H_2_-of R_2_), 51.72 (*C*H_2_-C_q_-2), 55.46 (*C*H_2_-C_q_-6), 122.16 (*C*_q_-2), 126.44 (*C*H Ph-p), 126.76 (*C*H Ph-p), 127.11 (*C*H Ph-p), 127.74 (*C*H Ph), 128.42 (*C*H Ph), 128.51 (*C*_q_-4), 128.64 (*C*H Ph), 128.65 (*C*H Ph), 128.80 (*C*H Ph), 128.94 (*C*H Ph), 129.32 (*C*H-3), 129.60 (*C*H-5), 138.46 (*C*_q_-1 Ph), 139.21 (*C*_q_-1 Ph), 152.77 (*C*_q_-1), 181.57 (*C*=S); HRMS (HESI^+^) *m*/*z* calcd. for C_33_H_38_N_3_OS^+^ [M + H]^+^ 524.2730, found 524.2727, ∆ = −0.3 mDa.

*Ligand **4cb**:* R_f_ 0.46 (DCM); colourless oil; ^1^H NMR 2.248 (s, 3H, C*H*_3_), 2.907 (bt, 4H, C*H*_2_-Ph of R_1_), 3.800 (bs, 4H, C*H*_2_-N of R_1_), 4.712 (d, 4H, J 4.7, C*H*_2_ of R_2_), 4.779 (bs, 4H, C*H*_2_-C_q_-2 and C*H*_2_-C_q_-6), 5.999 (bs, 2H, N*H*-CS), 6.878 (s, 2H, C*H*-3 and C*H*-5), 7.129–7.309 (m, 20H, C*H*-of 4Ph), 9.289 (bs, 1H, O*H*); ^13^C NMR 20.47 (*C*H_3_), 33.35 (*C*H_2_-Ph of R_1_), 50.44 (*C*H_2_-of R_2_*, C*H_2_-C_q_-2 and *C*H_2_-C_q_-6), 122.00 (*C*_q_-2 and *C*_q_-6), 126.88 (*C*H Ph-p), 127.60 (*C*H Ph-p), 127.92 (*C*H Ph), 128.69 (*C*H Ph), 128.92 (*C*H Ph), 129.00 (*C*_q_-4), 131.39 (*C*H-3 and *C*H-5), 137.65 (*C*_q_-1 Ph of R_2_), 138.45 (*C*_q_-1 Ph of R_1_), 151.25 (*C*_q_-1), 181.31 (*C*=S); HRMS (HESI^+^) *m*/*z* calcd. for C_41_H_45_N_4_OS_2_^+^ [M + H]^+^ 673.3029, found 673.3030, ∆ = 0.1 mDa.

*Ligand **3cc**:* R_f_ 0.16 (5% MeOH/DCM); colourless oil; ^1^H NMR 2.189 (s, 3H, C*H*_3_), 2.843–2.908 (m, 4H, C*H*_2_-Ph of R_1_), 2.931 (m, 2H, C*H*_2_-N of R_1_), 3.876 (m, 2H, C*H*_2_-NH of R_2_), 3.898 (s, 2H, C*H*_2_-C_q_-2), 4.060 (bs, 2H, C*H*_2_-NH of R_1_), 4.444 (bs, 2H, C*H*_2_-C_q_-6), 6.736 (s, 1H, C*H*-3), 6.823 (s, 1H, C*H*-5), 7.164–7.306 (m, 15H, C*H*-of 3Ph), 9.584 (bs, 1H, O*H*); ^13^C NMR 20.51 (*C*H_3_), 33.57 (*C*H_2_-Ph(NH) of R_1_), 35.27 (*C*H_2_-Ph(N) of R_1_ and *C*H_2_-Ph R_2_), 47.20 (*C*H_2_-NH R_2_), 48.30 (*C*H_2_-C_q_-6), 49.32 (*C*H_2_-N of R_1_), 51.99 (*C*H_2_-C_q_-2), 54.22 (*C*H_2_-NH of R_1_), 121.72 (*C*_q_-6), 122.03 (*C*_q_-2), 126.22 (*C*H Ph-p), 126.36 (*C*H Ph-p), 126.75 (*C*H Ph-p), 128.48 (*C*H Ph), 128.57 (*C*H Ph), 128.62 (*C*_q_-4), 128.65 (*C*H Ph), 128.78 (*C*H Ph), 128.81 (*C*H-5), 128.93 (*C*H-3), 139.28 (*C*_q_-1 2Ph), 152.96 (*C*_q_-1), 181.53 (*C*=S); HRMS (HESI^+^) *m*/*z* calcd. for C_34_H_40_N_3_OS^+^ [M + H]^+^ 538.2887, found 538.2887, ∆ = 0 mDa.

*Ligand **4cc**:* R_f_ 0.50 (DCM); colourless oil; ^1^H NMR 2.217 (s, 3H, C*H*_3_), 2.801 (t, 8H, J 7.1, C*H*_2_-Ph of R_1_ and R_2_), 3.796 (m, 8H, C*H*_2_-N of R_1_ and R_2_), 4.725 (bs, 4H, C*H*_2_-C_q_-2 and C*H*_2_-C_q_-6), 5.915 (bs, 2H, N*H*-CS), 6.823 (s, 2H, C*H*-3 and C*H*-5), 7.069–7.290 (m, 20H, C*H*-of 4Ph), 9.412 (bs, 1H, O*H*); ^13^C NMR 20.53 (*C*H_3_), 33.18 and 35.03 (*C*H_2_-Ph of R_1_ and R_2_), 47.23 (*C*H_2_-N of R_1_ and R_2_), 50.86 (*C*H_2_-C_q_-2 and *C*H_2_-C_q_-6), 121.99 (*C*_q_-2 and *C*_q_-6), 126.61 (*C*H Ph), 126.84 (*C*H Ph), 128.77 (few overlapped *C*H Ph), 128.89 (*C*H Ph), 128.97 (*C*_q_-4), 131.26 (*C*H-3 and *C*H-5), 138.40 and 138.71 (*C*_q_-1 4Ph), 151.23 (*C*_q_-1), 180.88 (*C*=S); HRMS (HESI^+^) *m*/*z* calcd. for C_43_H_49_N_4_OS_2_^+^ [M + H]^+^ 701.3342, found 701.3343, ∆ = 0.1 mDa.

### 3.3. Crystallography

Crystals of ligands **4aa**, **4ba**, **3bb** and **3bc** were grown by recrystallization from suitable solvents. Single crystals with appropriate size ((0.15 − 0.4) × (0.15 − 0.3) × (0.05 − 0.3) mm^3^) and diffraction quality were carefully selected and mounted on a nylon loop or glass capillary using cryoprotectant oil (Paratone). Diffraction data were collected on a D8 Venture diffractometer equipped with the Photon CPAD detector using micro-focus Mo*K*α radiation (λ = 0.71073 Å). Data were processed with CryAlisPro (41.117a-64bit) software [79]. The structures were solved with direct or intrinsic phasing methods and refined by the full-matrix least-squares method on *F*^2^ by using ShelxS, ShelxT and ShelxL program packages [80,81] as integrated in OLEX v.1.5 software [82]. All nonhydrogen atoms were located successfully from the Fourier map and were refined anisotropically. All hydrogen atoms riding on a parent carbon atom were placed in calculated positions using the following scheme: *U*_eq_ = 1.2 for C-H_aromatic_ = 0.93 Å, C-H_methyl_ = 0.96 Å and C-H_methylenic_ = 0.97 Å. The hydrogen atoms bonded to a heteroatom (nitrogen or oxygen) were located from the electron density maps [83]. The molecules in the asymmetric unit (ASU) were illustrated by ORTEP-3v2 software. Three-dimensional packing visualization of the molecules was made using CCDC Mercury [84]. The most important data collection and crystallographic refinement parameters are given in Tables S1–S9. Complete crystallographic data for the reported structures have been deposited in the CIF format with the CCDC as 2380793 to 2380796. These data can be obtained free of charge from The Cambridge Crystallographic Data Centre via www.ccdc.cam.ac.uk/structures (accessed on 30 August 2024).

### 3.4. Isothermal Titration Calorimetry (ITC)

All ITC experiments were conducted on an Affinity ITC isothermal titration calorimeter (TA Instruments, Northampton, MA, USA) at 298.15 K (25 °C). Calibration of the Affinity ITC calorimeter was carried out by using electrically generated heat pulses. The active cell volume of the calorimeter is 0.19 mL, with syringe volume up to 0.2 mL. The reference cell was filled with the nanopure water, conductivity not exceeding 0.18 µS cm^−1^ (Adrona, Riga, Latvia). The heat normalized per mole of injectant was processed with nanoAnalylze (TA Instruments). A CaCl_2_–EDTA titration (test kit TA Instruments) was performed in order to check the apparatus and results processing (*n*—stoichiometry, *K*d, Δ*H*). The typical experiment for assessing the **3** and **4** ligands includes 30 injections of 2.0 µL into the reaction cell. The reaction cell contains a ligand at a concentration of 0.025 mM, whereas the syringe contains a 0.125 mM solution of chloride or K^+^, Cs^+^, Ca^2+^ or Zn^2+^ salts. The first (initial) 2 µL injection was discarded from each data set to remove the effect of titrant diffusion and allow the equilibration process. Compounds **3** and **4** were dissolved and degassed prior to titration. The titrant was injected at a 200–300 s interval to ensure that the titration peak returned to the baseline before the next injection. The stirrer speed was kept constant at 125 rpm in order to achieve homogeneous mixing in the cell.

## 4. Conclusions

Two series of novel *N*,*O*,*S*-ligands possessing thiourea units attached to a *p*-cresol scaffold are obtained and characterized. The mono-acylated *bis*-amines **3** and symmetrical *bis*-thioureas **4** are prepared by a common two-step protocol and separated by chromatography. The transformation involves the initial synthesis of thiocarbamic chlorides **2** and further reaction with symmetrical *bis*-amines **1**. The proportions of the latter are varied, resulting in a predominance of monoacylated (**3**) or *bis*-substituted (**4**) compounds when using equimolar reagents’ amounts or excesses of the corresponding thiocarbamic chloride, respectively. It is observed that the conversion does not proceed cleanly and the products are generated in low to moderate overall yield; up to 66%. At the same time, it is found that the reaction output is strongly dependent on both *bis*-amine and thiocarbamic chloride substituents. The best total yields are achieved from *bis*-amines with phenyl (**1a**) and benzyl (**1b**) substituents, while those with phenethyl (**1c**) are lower in general, with the yield of *bis*-thioureas **4** being predominant. An extremely unfavourable case is the reaction with phenylthiocarbamic chloride **2a**, where mono-acylated ligands **3** are not detected from the reactions with both phenyl and phenethyl *bis*-amines **1a** and **1c**. The products are characterized by 1D and 2D NMR spectra in solution and by single crystal XRD of selected samples in solid state. It is shown that the S-atom in *bis*-thioureas **4** is involved in intramolecular O-H…S interactions, while in mono-acylated ligands **3** intermolecular N-H…S interactions operate. A preliminary study on the coordination properties of selected products in the neutral pH range performed by ICT shows that the ligands tested interact substantially only with potassium ions.

The synthetic protocol provides vast opportunities to obtain new entities by varying the substituents in both reagents.

## Figures and Tables

**Table 1:** Synthesis of ligands **3** and **4**.

Entry	Conditions ^a^	Products				
		3	Yield, %	4	Yield, %	Total, %
1	**1a:2a** 1:1	**3aa**	-	**4aa**	14	14
2	**1a**:**2a** 1:2		-		58	58
3	**1a**:**2a** 1:3		-		66	66
4	**1a**:**2b** 1:1	**3ab**	36	**4ab**	-	36
5	**1a**:**2b** 1:2		24		11	35
6	**1a**:**2b** 1:3		-		38	38
7	**1a**:**2c** 1:1	**3ac**	24	**4ac**	17	41
8	**1a**:**2c** 1:2		20		31	51
9	**1a**:**2c** 1:3		16		23	39
10	**1b**:**2a** 1:1	**3ba**	32	**4ba**	-	32
11	**1b**:**2a** 1:2		23		32	55
12	**1b**:**2a** 1:3		-		54	54
13	**1b**:**2b** 1:1	**3bb**	59	**4bb**	3	62
14	**1b**:**2b** 1:2		2		34	36
15	**1b**:**2b** 1:3		2		39	41
16	**1b**:**2c** 1:1	**3bc**	33	**4bc**	11	44
17	**1b**:**2c** 1:2		-		62	62
18	**1b**:**2c** 1:3		-		60	60
19	**1c**:**2a** 1:1	**3ca**	-	**4ca**	27	27
20	**1c**:**2a** 1:2		-		47	47
21	**1c**:**2a** 1:3		-		48	48
22	**1c**:**2b** 1:1	**3cb**	25	**4cb**	-	25
23	**1c**:**2b** 1:2		-		16	16
24	**1c**:**2b** 1:3		-		19	19
25	**1c**:**2c** 1:1	**3cc**	42	**4cc**	5	47
26	**1c**:**2c** 1:2		21		24	45
27	**1c**:**2c** 1:3		-		24	24

^a^ Common conditions: **2** from amine: thiophosgene 1:1, pyridine, benzene, rt, 24 h.

**Table 2:** Selected ^1^H and ^13^C NMR resonances (ppm) of the ligands **3** and **4**.

Ligand	C*H*-3/*C*H-3	C*H*-5/*C*H-5	C*H*_2_-C_q_-2/*C*H_2_-C_q_-2	C*H*_2_-C_q_-6/*C*H_2_-C_q_-6
**4aa**	6.622/131.69		5.472/54.36	
**3ab**	6.263/131.38	6.991/130.09	5.441/55.67	4.360/45.29
**4ab**	6.601/131.16		5.418/54.45	
**3ac**	6.978/130.01	6.210/131.37	5.365/55.31	4.344/45.06
**4ac**	6.559/130.95		5.349/54.05	
**3ba**	6.850/131.15	6.830/129.68	4.645 br *	4.005/51.35
**4ba**	6.941/133.00		5.017 br *	
**3bb**	6.770/129.88	6.713/129.26	4.518 br *	3.785/51.24
**4bb**	6.824/131.77		4.893 br *	
**3bc**	6.763 (common)/129.82 (common)		4.497 br *	5.213 br *
**4bc**	6.776/131.75		4.836 br *	
**4ca**	7.027/132.78		4.956 br *	
**3cb**	6.725/129.32	6.895/129.60	3.805/51.72	4.187/55.46
**4cb**	6.878/131.39		4.779/50.44	
**3cc**	6.736/128.93	6.823/128.81	3.898/51.99	4.444/48.30
**4cc**	6.823/131.26		4.725/50.86	

* Broad signals in ^1^H spectra; the corresponding signals do not appear in ^13^C and 2D spectra.

**Table 3:** Details about the geometrical parameters of the π…π interactions observed in compounds **3bb**, **3bc**, **4aa** and **4ba**; provided are the atoms participating in rings (Ring A and Ring B), the centroid-to-centroid (Cg…Cg) distances in Å, and the displacement (shift) between the rings in Å where applicable. Symmetry operations related to each interaction are provided below the table.

	π…π Type	Ring A	Ring B	Cg…Cg	Shift
				Å	Å
**3bb**	T-shape	C20-C21-C23-C22-C18-C19	C28-C27-C29-C31-C30-C26 ^1^	4.084	–
	T-shape	C20-C21-C23-C22-C18-C19	C20-C21-C23-C22-C18-C19 ^1^	3.909	–
**3bc**	T-shape	C151-C101-C121-C141-C131-C111	C52-C62-C42-C72-C32-C22 ^2^	3.726	–
	T-shape	C191-C201-C241-C211-C221-C231	C302-C292-C272-C322-C312-C282 ^2^	3.791	–
**4aa**	Parallel displaced	C2-C3-C4-C5-C6-C7	C2-C3-C4-C5-C6-C7 ^3^	3.807	1.357
**4ba**	T-shape	C32-C36-C35-C37-C34-C33	C30-C28-C29-C27-C26-C25 ^4^	3.919	–

Symmetry operations: ^1^ *x*, 1 − *y*, −1/2 + *z*, ^2^ −1 + *x*, *y*, *z*, ^3^ −*x*, −*y*, 1 − *z*, ^4^ 1 − *x*, 2 − *y*, 1 − *z*.

**Table 4:** Summary of the ITC results for the titration of KCl with **3ab**, **3ac**, **3ba**, **3bb**, **3bc**, **4aa**, **4ac**, **4ba** and **4bc** derivatives; all interactions are conducted at 25 °C with 125 rpm continuous stirring; Ka is the association constant and n represents the stoichiometry, for example, the number of molecules that associate with a metal ion.

Cell Contents (µM)	Syringe Contents (µM)	*K*a. 10^6^ (M^−1^)	N *
**3ab** 0.25	KCl 1.25	1.89 ± 0.52	2.114
**3ac** 0.25	KCl 1.25	1.02 ± 0.41	1.996
**3ba** 0.25	KCl 1.25	1.57 ± 0.57	2.070
**3bb** 0.25	KCl 1.25	2.31 ± 0.84	2.193
**3bc** 0.25	KCl 1.25	0.87 ± 0.61	1.919
**4aa** 0.25	KCl 1.25	1.22 ± 0.31	2.165
**4ac** 0.25	KCl 1.25	2.29 ± 0.58	1.582
**4ba** 0.25	KCl 1.25	1.92 ± 0.65	2.364
**4bc** 0.25	KCl 1.25	0.98 ± 0.64	2.037

* Independent model used.

## Data Availability

The data of the current study are available from the corresponding authors on reasonable request.

## Supplementary Materials

The following supporting information can be downloaded at: https://www.mdpi.com/article/10.3390/molecules29204906/s1, NMR data (Figures S1 and S2), crystallographic data (Tables S1–S13 and Figures S3–S7), original NMR spectra (Figures S8–S86) and original HESI HRMS spectra (Figures S87–S102).
