# Supplementary material for: Novel Thiourea Ligands—Synthesis, Characterization and Preliminary Study on Their Coordination Abilities
Source: Molecules. 2024 Oct 16;29(20):4906. doi: 10.3390/molecules29204906 (PMC11510064; doi:10.3390/molecules29204906)
Supplement: Supplementary file 1 [file molecules-29-04906-s001.zip › molecules-3218984-supplementary.pdf]

SUPPLEMANTARY MATERIAL

Novel thiourea ligands – synthesis, characterisation and preliminary study on their coordination abilities

Stanislava E. Todorova[1], Rusi I. Rusew[2], Zhanina S. Petkova[1,3], Boris L. Shivachev[2,*], Vanya B. Kurteva[1,*]

[1]Institute of Organic Chemistry with Centre of Phytochemistry, Bulgarian Academy of Sciences, Acad. G. Bonchev str., bl. 9, 1113 Sofia, Bulgaria.
[2]Institute of Mineralogy and Crystallography "Acad. Ivan Kostov", Bulgarian Academy of Sciences, Acad. G. Bonchev str., bl. 107, 1113 Sofia, Bulgaria.
[3]Centre of Competence "Sustainable Utilization of Bio-resources and Waste of Medicinal and Aromatic Plants for Innovative Bioactive Products" (CoC BioResources), Acad. G. Bonchev Str., bl. 9, 1113 Sofia, Bulgaria.

Table of Contents

**Content**	**Page**
NMR data	2
Crystallographic data	3
Original NMR spectra	15
Original HRMS spectra	54

NMR data

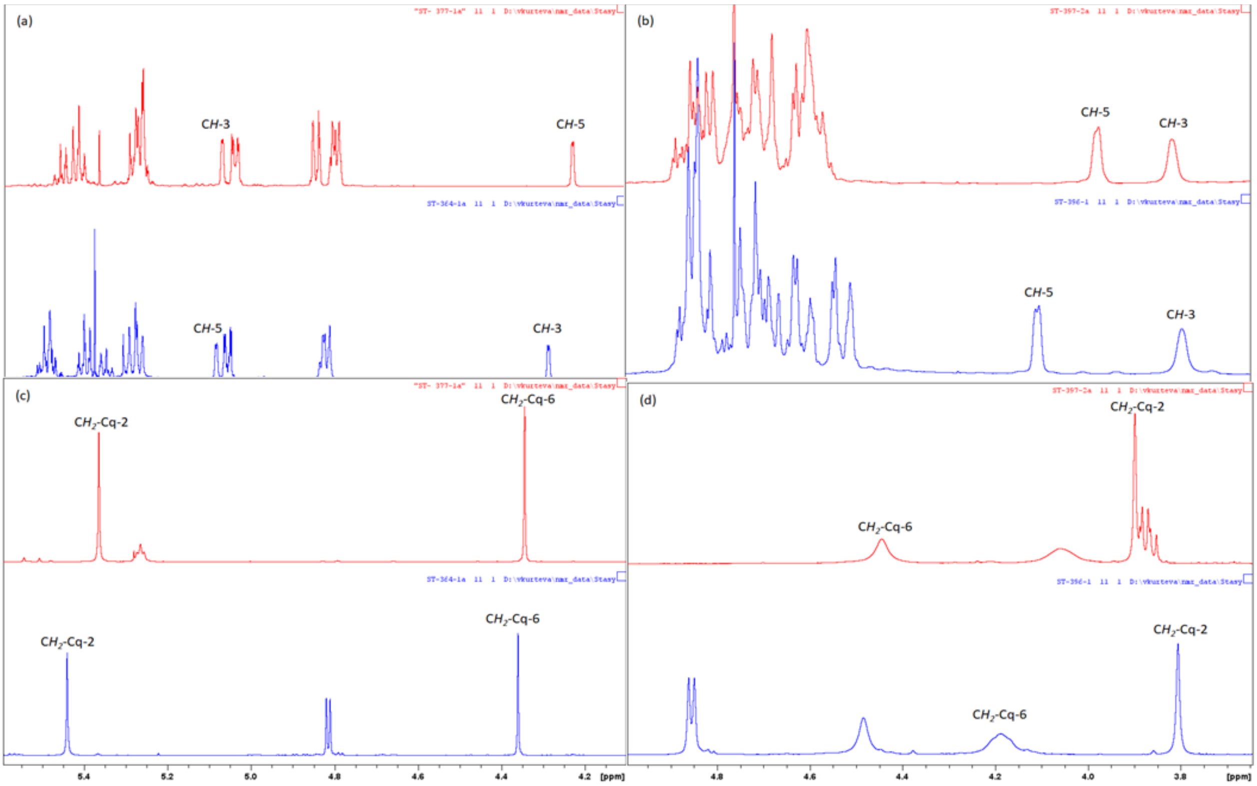

Figure S1. Partial ^{1}H NMR spectra: CH-3+5 area of a) **3ab** (blue) and **3ac** (red), b) **3cb** (blue) and **3cc** (red), and CH_2-C_q-2+6 area of c) **3ab** (blue) and **3ac** (red), d) **3cb** (blue) and **3cc** (red).

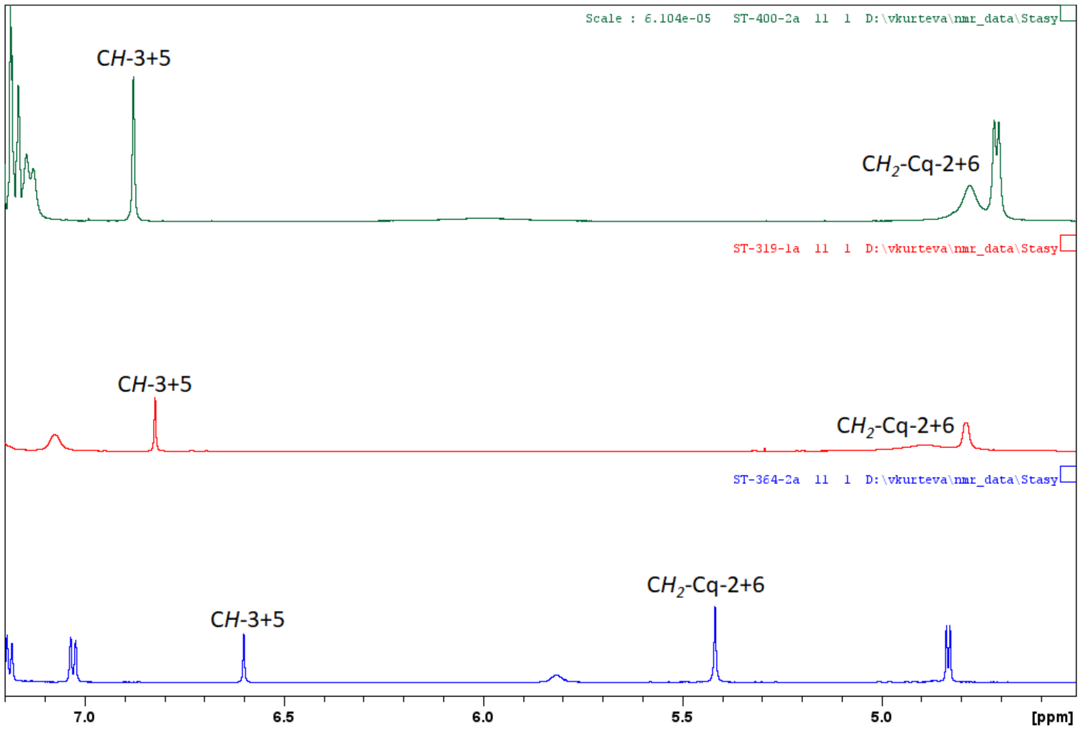

Figure S2. Partial ^{1}H NMR spectra of **4ab** (blue), **4bb** (red), and **4cb** (green).

Crystallographic data

Table S1. Most important data collection and crystallographic refinement parameters for compounds **4aa**, **4ba**, **3bb** and **3bc**.

Identification code	B_0192	B_0193	B_0425	424b
Identification code	**4aa**	**4ba**	**3bb**	**3bc**
Empirical formula	$C_{35}H_{32}N_4OS_2$	$C_{37}H_{36}N_4OS_2$	$C_{31}H_{33}N_3OS$	$C_{32}H_{35}N_3OS$
Formula weight	588.76	616.82	495.66	509.69
Temperature/K	290	290	290	290
Crystal system	Triclinic	Triclinic	Monoclinic	Triclinic
Space group	*P*-1	*P*-1	*Pc*	*P*-1
a/Å	8.605(3)	10.492(7)	14.082(5)	8.4971(4)
b/Å	10.804(4)	10.541(7)	11.443(4)	15.2378(6)
c/Å	16.619(7)	15.350(1)	8.670(3)	22.0447(10)
α/°	100.417(1)	88.479(3)	90	95.7980(10)
β/°	99.541(1)	74.632(3)	96.942(1)	90.634(2)
γ/°	90.989(1)	79.518(3)	90	97.1950(10)
Volume/Å^3	1496.78(1)	1609.25(2)	1386.9(8)	2816.5(2)
Z	2	2	2	4
ρ_{calc}g/cm^3	1.306	1.273	1.187	1.202
μ/mm^{-1}	0.213	0.202	0.144	0.144
F(000)	620.0	652.0	528.0	1088.0
Crystal size/mm^3	0.3×0.2×0.2	0.3×0.2×0.2	0.4×0.3×0.3	0.4 × 0.1 × 0.05
Radiation	Mo*K*α λ = 0.71073	Mo*K*α λ = 0.71073	Mo*K*α λ = 0.71073	MoKα (λ = 0.71073)
2Θ range for data collection/°	4.184 – 50.048	4.286 – 50.448	4.6 – 52.04	4.366 – 52.788
Reflections collected/independent	38472/ 5281	28627/5704	32163/5452	146458/11526
R_{int} / R_{sigma}	0.0321/0.0236	0.0338/0.0255	0.0689/0.0470	0.0951/ 0.0423
Data/restraints/parameters	5281/2/478	5704/0/410	5452/2/338	11526/0/694
Goodness-of-fit on F^2	1.050	1.062	1.159	1.050
Final *R* indexes [*I*>=2σ (*I*)]	R_1 = 0.0371 wR_2 = 0.0939	R_1 = 0.0454, wR_2 = 0.1188	R_1 = 0.0600, wR_2 = 0.1258	R_1= 0.0515, wR_2= 0.1108
Final *R* indexes [all data]	R_1 = 0.0424 wR_2 = 0.0995	R_1 = 0.0509, wR_2 = 0.1229	R_1 = 0.0708, wR_2 = 0.1302	R_1= 0.0922, wR_2= 0.1313
Largest diff. peak/hole / e Å^{-3}	0.22/-0.22	0.32/-0.35	0.25/-0.16	0.22/-0.1
CCDC	2380795	2380793	2380794	2380796

Table S2. Bond Lengths for **3bb**.

Atom	Atom	Length/Å	Atom	Atom	Length/Å
S1	C16	1.695(4)	C7	C6	1.385(7)
O5	C5	1.375(6)	C17	C18	1.481(8)
N1	C9	1.464(6)	C2	C1	1.518(8)
N1	C8	1.481(5)	C11	C12	1.385(7)
N1	C16	1.344(6)	C18	C23	1.377(8)
N3	C24	1.472(6)	C18	C19	1.380(8)
N3	C25	1.480(7)	C23	C22	1.372(1)
N2	C17	1.446(7)	C31	C30	1.374(1)
N2	C16	1.352(6)	C12	C13	1.371(1)
C24	C4	1.516(7)	C19	C20	1.379(9)
C9	C10	1.513(6)	C27	C28	1.387(1)
C3	C2	1.376(7)	C15	C14	1.390(1)
C3	C4	1.380(7)	C22	C21	1.376(1)
C8	C6	1.502(6)	C14	C13	1.365(1)
C26	C25	1.484(9)	C28	C29	1.359(1)
C26	C31	1.362(8)	C30	C29	1.370(1)
C26	C27	1.394(9)	C20	C21	1.358(1)
C10	C11	1.390(7)	C5	C4	1.398(6)
C10	C15	1.381(7)	C5	C6	1.393(6)
C7	C2	1.381(7)			

Table S3. Bond Lengths for **3bc**.

Atom	Atom	Length/Å	Atom	Atom	Length/Å
S11	C161	1.692(2)	C22	C72	1.386(3)
S12	C162	1.687(2)	C22	C32	1.383(3)
O51	C51	1.360(3)	C22	C12	1.507(3)
O52	C52	1.365(3)	C71	C21	1.389(3)
N11	C91	1.461(3)	C271	C261	1.501(3)
N11	C81	1.475(3)	C271	C281	1.377(3)
N11	C161	1.356(3)	C271	C321	1.374(3)
N12	C162	1.357(3)	C31	C21	1.384(3)
N12	C82	1.470(3)	C112	C122	1.380(3)
N12	C92	1.462(3)	C21	C11	1.507(3)
N31	C251	1.469(3)	C121	C131	1.375(4)
N31	C261	1.462(3)	C122	C132	1.362(4)
N32	C252	1.465(3)	C192	C182	1.505(4)
N32	C262	1.459(3)	C192	C202	1.361(4)
N21	C161	1.336(3)	C192	C242	1.358(4)
N21	C171	1.447(3)	C191	C181	1.504(3)
N22	C162	1.341(3)	C191	C201	1.378(4)
N22	C172	1.454(3)	C191	C241	1.372(4)
C51	C61	1.393(3)	C151	C141	1.381(4)
C51	C41	1.405(3)	C171	C181	1.492(3)
C52	C62	1.390(3)	C322	C312	1.380(4)
C52	C42	1.402(3)	C172	C182	1.491(3)
C61	C81	1.512(3)	C281	C291	1.372(4)
C61	C71	1.384(3)	C131	C141	1.364(4)
C62	C82	1.512(3)	C321	C311	1.378(4)
C62	C72	1.381(3)	C152	C142	1.377(4)
C101	C91	1.504(3)	C282	C292	1.377(4)
C101	C111	1.388(3)	C201	C211	1.389(4)
C101	C151	1.381(3)	C241	C231	1.368(4)
C41	C251	1.510(3)	C312	C302	1.362(4)
C41	C31	1.377(3)	C302	C292	1.369(4)
C42	C32	1.380(3)	C311	C301	1.363(4)
C42	C252	1.512(3)	C202	C212	1.383(5)
C92	C102	1.502(3)	C132	C142	1.373(4)
C102	C112	1.387(3)	C211	C221	1.359(4)
C102	C152	1.378(3)	C301	C291	1.374(4)
C272	C262	1.505(3)	C221	C231	1.355(4)
C272	C322	1.374(3)	C242	C232	1.372(5)
C272	C282	1.376(3)	C212	C222	1.359(6)
C111	C121	1.381(3)	C222	C232	1.341(6)

Table S4. Bond Lengths for **4aa**.

Atom	Atom	Length/Å	Atom	Atom	Length/Å
S2	C29	1.6672(16)	C30	C35B	1.396(2)
S1	C15	1.6814(17)	C24	C25	1.384(3)
O5	C5	1.3702(19)	C10	C11	1.373(3)
N3	C29	1.361(2)	C28	C27	1.383(3)
N3	C23	1.439(2)	C14	C13	1.393(3)
N3	C22	1.465(2)	C16A	C21A	1.31(2)
N1	C9	1.435(2)	C16A	C17A	1.64(2)
N1	C8	1.482(2)	C25	C26	1.363(3)
N1	C15	1.356(2)	C26	C27	1.373(4)
N4	C29	1.354(2)	C11	C12	1.354(4)
N4	C30	1.426(2)	C12	C13	1.365(4)
N2	C15	1.350(2)	C33	C34A	1.398(6)
N2	C16A	1.37(2)	C33	C32A	1.318(5)
N2	C16B	1.51(3)	C33	C32B	1.503(16)
C5	C4	1.396(2)	C33	C34B	1.216(11)
C5	C6	1.397(2)	C21A	C20A	1.402(9)
C4	C3	1.387(2)	C35A	C34A	1.391(5)
C4	C22	1.513(2)	C17A	C18A	1.377(9)
C6	C7	1.387(2)	C31A	C32A	1.383(5)
C6	C8	1.506(2)	C20A	C19A	1.355(11)
C3	C2	1.391(2)	C19A	C18A	1.376(10)
C9	C10	1.385(2)	C31B	C32B	1.383(15)
C9	C14	1.373(2)	C35B	C34B	1.349(13)
C7	C2	1.383(2)	C16B	C21B	1.67(3)
C2	C1	1.503(2)	C16B	C17B	1.12(3)
C23	C24	1.381(2)	C21B	C20B	1.363(15)
C23	C28	1.374(3)	C19B	C20B	1.372(19)
C30	C35A	1.363(4)	C19B	C18B	1.323(18)
C30	C31A	1.352(4)	C17B	C18B	1.402(12)
C30	C31B	1.396(9)			

Table S5. Bond Lengths for **4ba**.

Atom	Atom	Length/Å	Atom	Atom	Length/Å
S1	C16	1.6798(18)	C17	C22	1.384(3)
S2	C31	1.685(2)	C17	C18	1.373(3)
O5	C5	1.363(2)	C22	C21	1.369(3)
N4	C32	1.424(3)	C21	C20	1.370(4)
N4	C31	1.356(3)	C20	C19	1.367(4)
N3	C31	1.351(3)	C3	C2	1.387(3)
N3	C23	1.476(2)	C2	C7	1.385(3)
N3	C24	1.461(3)	C2	C1	1.503(3)
C4	C23	1.508(3)	C9	C10	1.501(3)
C4	C5	1.396(3)	C10	C15	1.374(3)
C4	C3	1.387(3)	C10	C11	1.376(3)
C6	C5	1.398(2)	C15	C14	1.383(4)
C6	C8	1.514(3)	C14	C13	1.344(5)
C6	C7	1.388(3)	C13	C12	1.354(5)
N1	C8	1.476(2)	C12	C11	1.389(3)
N1	C16	1.357(2)	C19	C18	1.381(3)
N1	C9	1.459(2)	C24	C25	1.512(3)
N2	C16	1.357(3)	C25	C30	1.377(3)
N2	C17	1.420(3)	C25	C26	1.380(3)
C35	C36	1.367(4)	C30	C29	1.379(3)
C35	C34	1.364(4)	C29	C28	1.361(4)
C36	C37	1.385(3)	C28	C27	1.369(5)
C37	C32	1.379(3)	C27	C26	1.383(4)
C32	C33	1.377(3)	C33	C34	1.381(4)

Table S6. Bond Angles for **3bb**.

Atom	Atom	Atom	Angle/°	Atom	Atom	Atom	Angle/°
C9	N1	C8	114.6(3)	C26	C31	C30	121.1(7)
C16	N1	C9	121.0(4)	C13	C12	C11	120.5(6)
C16	N1	C8	124.3(4)	C20	C19	C18	122.0(6)
C24	N3	C25	111.9(4)	C28	C27	C26	121.5(6)
C16	N2	C17	123.1(4)	C10	C15	C14	120.3(6)
N3	C24	C4	111.6(4)	C23	C22	C21	121.7(7)
N1	C9	C10	115.1(4)	C13	C14	C15	120.8(6)
C2	C3	C4	122.1(4)	C29	C28	C27	120.5(7)
N1	C8	C6	115.7(3)	C29	C30	C31	122.0(7)
C31	C26	C25	121.6(6)	C14	C13	C12	119.5(6)
C31	C26	C27	117.0(6)	C28	C29	C30	118.0(8)
C27	C26	C25	121.5(6)	C21	C20	C19	120.0(7)
C11	C10	C9	122.3(4)	C20	C21	C22	118.6(7)
C15	C10	C9	119.1(5)	O5	C5	C4	120.4(4)
C15	C10	C11	118.5(5)	O5	C5	C6	118.9(4)
N3	C25	C26	112.3(5)	C6	C5	C4	120.7(4)
C2	C7	C6	122.9(5)	C3	C4	C24	121.0(4)
N2	C17	C18	115.4(5)	C3	C4	C5	118.8(4)
C3	C2	C7	117.7(4)	C5	C4	C24	120.1(4)
C3	C2	C1	121.1(5)	N1	C16	S1	122.0(3)
C7	C2	C1	121.2(5)	N1	C16	N2	116.3(4)
C12	C11	C10	120.4(5)	N2	C16	S1	121.6(4)
C23	C18	C17	116.9(5)	C7	C6	C8	120.9(4)
C23	C18	C19	117.5(6)	C7	C6	C5	117.8(4)
C19	C18	C17	125.6(5)	C5	C6	C8	121.2(4)
C22	C23	C18	120.2(7)				

Table S7. Bond Angles for **3bc**.

Atom	Atom	Atom	Angle/°	Atom	Atom	Atom	Angle/°
C91	N11	C81	114.78(17)	C61	C71	C21	122.4(2)
C161	N11	C91	121.76(17)	C42	C32	C22	122.5(2)
C161	N11	C81	123.29(17)	C281	C271	C261	118.8(2)
C162	N12	C82	123.51(18)	C321	C271	C261	123.5(2)
C162	N12	C92	121.65(18)	C321	C271	C281	117.7(2)
C92	N12	C82	114.80(18)	N31	C251	C41	113.19(18)
C261	N31	C251	112.31(19)	C41	C31	C21	122.6(2)
C262	N32	C252	113.29(19)	N32	C252	C42	112.20(18)
C161	N21	C171	123.7(2)	C122	C112	C102	120.8(2)
C162	N22	C172	123.3(2)	C71	C21	C11	121.1(2)
O51	C51	C61	119.12(18)	C31	C21	C71	117.5(2)
O51	C51	C41	120.4(2)	C31	C21	C11	121.4(2)
C61	C51	C41	120.5(2)	C131	C121	C111	120.4(2)
O52	C52	C62	119.08(19)	C132	C122	C112	120.3(3)
O52	C52	C42	120.38(19)	N32	C262	C272	114.0(2)

Table S7. Bond Angles for **3bc**.

Atom	Atom	Atom	Angle/°	Atom	Atom	Atom	Angle/°
C62	C52	C42	120.5(2)	C202	C192	C182	121.3(3)
C51	C61	C81	121.47(19)	C242	C192	C182	120.6(3)
C71	C61	C51	118.51(19)	C242	C192	C202	118.1(3)
C71	C61	C81	119.9(2)	C201	C191	C181	121.4(3)
C52	C62	C82	121.5(2)	C241	C191	C181	120.5(3)
C72	C62	C52	118.5(2)	C241	C191	C201	118.0(3)
C72	C62	C82	119.86(19)	C141	C151	C101	120.9(2)
C111	C101	C91	121.4(2)	N31	C261	C271	114.3(2)
C151	C101	C91	120.4(2)	N21	C171	C181	110.7(2)
C151	C101	C111	118.1(2)	C272	C322	C312	120.9(3)
C51	C41	C251	120.9(2)	N22	C172	C182	110.9(2)
C31	C41	C51	118.5(2)	C291	C281	C271	121.6(3)
C31	C41	C251	120.36(19)	C141	C131	C121	119.5(3)
N11	C91	C101	114.79(17)	C271	C321	C311	121.1(3)
C52	C42	C252	121.0(2)	C171	C181	C191	110.6(2)
C32	C42	C52	118.5(2)	C142	C152	C102	121.1(3)
C32	C42	C252	120.4(2)	C272	C282	C292	120.8(3)
N12	C162	S12	122.83(17)	C191	C201	C211	120.3(3)
N22	C162	S12	121.48(18)	C172	C182	C192	112.4(2)
N22	C162	N12	115.6(2)	C231	C241	C191	121.5(3)
N12	C82	C62	116.31(17)	C131	C141	C151	120.6(3)
N11	C81	C61	116.19(17)	C302	C312	C322	120.8(3)
N11	C161	S11	122.73(17)	C312	C302	C292	118.6(3)
N21	C161	S11	121.51(17)	C301	C311	C321	120.4(3)
N21	C161	N11	115.69(19)	C302	C292	C282	120.9(3)
N12	C92	C102	115.99(18)	C192	C202	C212	121.1(3)
C112	C102	C92	121.8(2)	C122	C132	C142	119.8(3)
C152	C102	C92	120.0(2)	C221	C211	C201	119.8(3)
C152	C102	C112	118.0(2)	C132	C142	C152	120.1(3)
C322	C272	C262	122.9(2)	C311	C301	C291	119.4(3)
C322	C272	C282	118.0(3)	C281	C291	C301	119.9(3)
C282	C272	C262	119.1(2)	C231	C221	C211	120.4(3)
C121	C111	C101	120.6(2)	C221	C231	C241	119.9(3)
C72	C22	C12	121.2(2)	C192	C242	C232	120.9(4)
C32	C22	C72	117.4(2)	C222	C212	C202	119.6(4)
C32	C22	C12	121.4(2)	C232	C222	C212	119.5(4)
C62	C72	C22	122.6(2)	C222	C232	C242	120.9(4)

Table S8. Bond Angles for **4aa**.

Atom	Atom	Atom	Angle/°	Atom	Atom	Atom	Angle/°
C29	N3	C23	121.73(13)	C31B	C30	N4	116.4(5)
C29	N3	C22	121.53(13)	C35B	C30	N4	124.9(4)
C23	N3	C22	116.62(12)	C35B	C30	C31B	117.8(6)
C9	N1	C8	116.30(13)	C23	C24	C25	119.5(2)

Table S8. Bond Angles for **4aa**.

Atom	Atom	Atom	Angle/°	Atom	Atom	Atom	Angle/°
C15	N1	C9	121.16(14)	C11	C10	C9	120.4(2)
C15	N1	C8	122.46(14)	C23	C28	C27	119.6(2)
C29	N4	C30	126.26(14)	C9	C14	C13	118.6(2)
C15	N2	C16A	126.8(10)	N2	C16A	C17A	110.6(14)
C15	N2	C16B	129.2(13)	C21A	C16A	N2	134(2)
O5	C5	C4	116.25(13)	C21A	C16A	C17A	107.1(14)
O5	C5	C6	123.23(13)	C26	C25	C24	120.6(2)
C4	C5	C6	120.47(14)	C25	C26	C27	119.79(19)
C5	C4	C22	118.19(14)	C12	C11	C10	120.4(2)
C3	C4	C5	118.85(14)	C11	C12	C13	119.8(2)
C3	C4	C22	122.90(14)	C26	C27	C28	120.4(2)
C5	C6	C8	122.38(14)	C12	C13	C14	121.2(2)
C7	C6	C5	118.45(14)	C32A	C33	C34A	119.3(3)
C7	C6	C8	119.16(14)	C34B	C33	C32B	119.8(6)
C4	C3	C2	122.10(15)	C16A	C21A	C20A	130.5(11)
C10	C9	N1	119.47(15)	C30	C35A	C34A	119.5(3)
C14	C9	N1	120.86(16)	C18A	C17A	C16A	119.4(10)
C14	C9	C10	119.64(17)	C35A	C34A	C33	119.7(3)
C2	C7	C6	122.66(15)	C30	C31A	C32A	120.7(3)
N3	C29	S2	122.82(12)	C19A	C20A	C21A	118.5(6)
N4	C29	S2	122.14(12)	C33	C32A	C31A	121.1(3)
N4	C29	N3	115.03(14)	C20A	C19A	C18A	120.7(5)
C3	C2	C1	121.60(15)	C19A	C18A	C17A	121.1(5)
C7	C2	C3	117.46(15)	C32B	C31B	C30	121.1(9)
C7	C2	C1	120.94(15)	C31B	C32B	C33	115.4(8)
C24	C23	N3	120.75(16)	C34B	C35B	C30	119.0(7)
C28	C23	N3	119.17(15)	C33	C34B	C35B	126.2(9)
C28	C23	C24	120.07(16)	N2	C16B	C21B	95.3(15)
N1	C8	C6	113.36(12)	C17B	C16B	N2	128(3)
N3	C22	C4	114.94(13)	C17B	C16B	C21B	119(2)
N1	C15	S1	123.71(13)	C20B	C21B	C16B	108.2(13)
N2	C15	S1	121.60(13)	C18B	C19B	C20B	120.8(9)
N2	C15	N1	114.68(15)	C21B	C20B	C19B	121.1(9)
C35A	C30	N4	117.5(2)	C16B	C17B	C18B	119.0(18)
C31A	C30	N4	122.9(2)	C19B	C18B	C17B	121.0(11)
C31A	C30	C35A	119.6(3)				

Table S9. Bond Angles for **4ba**.

Atom	Atom	Atom	Angle/°	Atom	Atom	Atom	Angle/°
C31	N4	C32	128.3(2)	C18	C17	N2	118.2(2)
C31	N3	C23	121.6(2)	C18	C17	C22	119.3(2)
C31	N3	C24	122.6(2)	C21	C22	C17	119.9(2)
C24	N3	C23	115.5(2)	C22	C21	C20	120.7(2)
C5	C4	C23	122.2(2)	C19	C20	C21	119.8(2)

Table S9. Bond Angles for **4ba**.

Atom	Atom	Atom	Angle/°	Atom	Atom	Atom	Angle/°
C3	C4	C23	119.3(2)	C2	C3	C4	122.5(2)
C3	C4	C5	118.5(2)	C3	C2	C1	120.9(2)
C5	C6	C8	120.9(2)	C7	C2	C3	117.4(2)
C7	C6	C5	118.4(2)	C7	C2	C1	121.7(2)
C7	C6	C8	120.7(2)	C2	C7	C6	122.6(2)
C16	N1	C8	124.3(2)	N1	C9	C10	114.4(2)
C16	N1	C9	121.1(2)	C15	C10	C9	119.0(2)
C9	N1	C8	114.7(2)	C15	C10	C11	118.3(2)
C16	N2	C17	127.4(2)	C11	C10	C9	122.7(2)
C34	C35	C36	119.6(2)	C10	C15	C14	120.6(3)
C35	C36	C37	120.8(3)	C13	C14	C15	120.8(3)
C32	C37	C36	119.4(2)	C14	C13	C12	119.6(3)
C37	C32	N4	122.9(2)	C13	C12	C11	120.8(3)
C33	C32	N4	117.4(2)	C10	C11	C12	120.0(2)
C33	C32	C37	119.6(2)	C20	C19	C18	120.0(2)
N4	C31	S2	122.2(2)	C17	C18	C19	120.3(2)
N3	C31	S2	122.5(2)	N3	C24	C25	114.7(2)
N3	C31	N4	115.3(2)	C30	C25	C24	121.9(2)
N3	C23	C4	113.0(2)	C30	C25	C26	117.9(2)
O5	C5	C4	122.8(2)	C26	C25	C24	120.2(2)
O5	C5	C6	116.5(2)	C25	C30	C29	121.3(2)
C4	C5	C6	120.6(2)	C28	C29	C30	120.1(3)
N1	C8	C6	114.3(2)	C29	C28	C27	119.6(3)
N1	C16	S1	122.7(1)	C28	C27	C26	120.4(2)
N2	C16	S1	121.9(1)	C25	C26	C27	120.6(3)
N2	C16	N1	115.5(2)	C32	C33	C34	120.1(3)
C22	C17	N2	122.3(2)	C35	C34	C33	120.5(3)

Table S10. Hydrogen Bonding interactions for **3bb**.

D	H	A	d(D-H)/Å	d(H-A)/Å	d(D-A)/Å	D-H-A/°
O5	H5	N3	0.76(6)	1.86(6)	2.573(5)	156(6)
N3	H3	S1[1]	0.92(4)	2.53(4)	3.384(5)	154(3)
N2	H2	O5	0.80(5)	2.10(6)	2.885(6)	167(5)
C25	H25A	S1[2]	0.97	2.96	3.896(6)	161.3

Symmetry operations:[1]+X,1-Y,-1/2+Z; [2]+X,-1+Y,+Z

Table S11. Hydrogen Bonding interactions for **3bc**.

D	H	A	d(D-H)/Å	d(H-A)/Å	d(D-A)/Å	D-H-A/°
N31	H31	S12[1]	0.86(3)	2.59(3)	3.434(2)	166(2)
O51	H51	N31	0.94(4)	1.69(4)	2.572(2)	155(3)
N32	H32	S11	0.90(3)	2.55(3)	3.428(2)	169(2)
O52	H52	N32	0.93(3)	1.70(3)	2.577(2)	156(3)
N22	H22	O52	0.91(3)	1.95(3)	2.829(3)	161(2)

Table S11. Hydrogen Bonding interactions for **3bc**.

D	H	A	d(D-H)/Å	d(H-A)/Å	d(D-A)/Å	D-H-A/°
N21	H21	O51	0.92(3)	1.91(3)	2.791(3)	159(2)

Symmetry operation: [1]1+X,+Y,+Z

Symmetry operation: [1]-X,-Y,1-Z

Table S12. Hydrogen Bonding interactions for **4ba**.

D	H	A	d(D-H)/Å	d(H-A)/Å	d(D-A)/Å	D-H-A/°
C23	H23B	S2	0.97	2.53	3.051(2)	113.6
C9	H9A	S1	0.97	2.49	3.028(2)	115.1
N2	H2	O5	0.82(2)	2.05(2)	2.869(2)	175(2)
O5	H5	S2	0.79(3)	2.31(3)	3.090(2)	171(2)

Table S13. Hydrogen Bonding interactions for **4aa**.

D	H	A	d(D-H)/Å	d(H-A)/Å	d(D-A)/Å	D-H-A/°
O5	H5	S1	0.82	2.36	3.170(1)	168.0
C8	H8A	S1	0.97	2.61	3.105(2)	112.0
C8	H8B	S2[1]	0.97	2.99	3.956(2)	171.2
C14	H14	S2[1]	0.93	2.86	3.757(2)	162.5

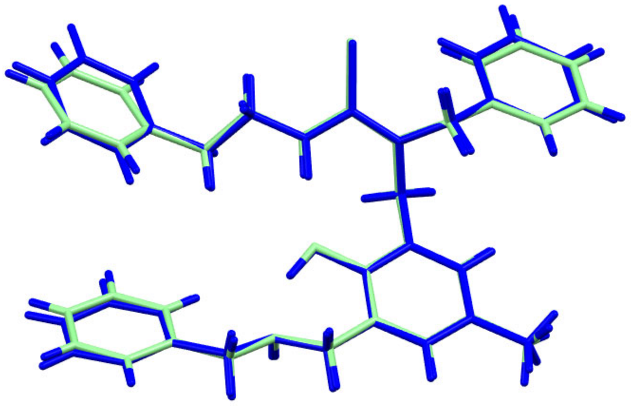

Figure S3. Visualization of the overlay of the molecules present in the asymmetric unit of **3bc**; rmsd is 0.1961 Å.

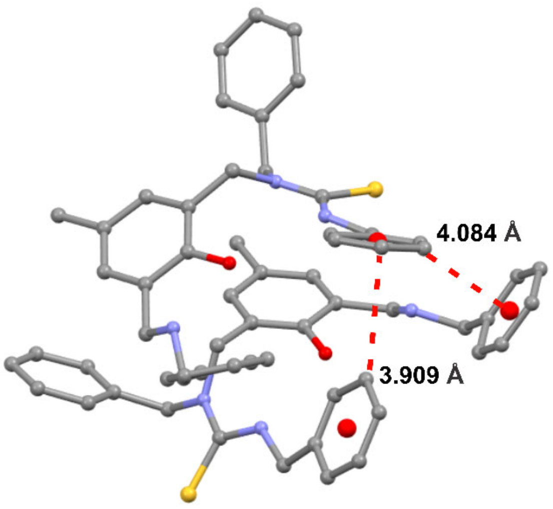

Figure S4. Visualization of the T-shape π... π interactions present in **3bb**.

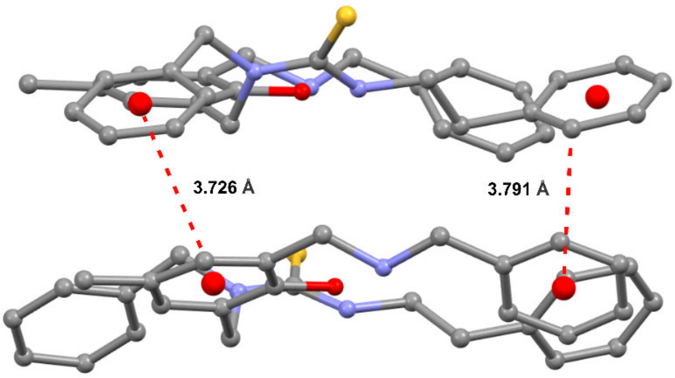

Figure S5. Visualization of the T-shape π... π interactions present in **3bc**.

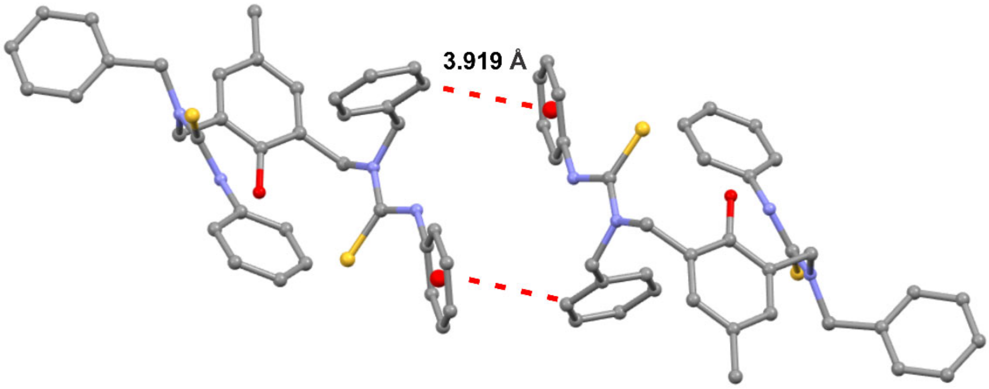

Figure S6. Visualization of the T-shape π... π interactions present in **4ba**.

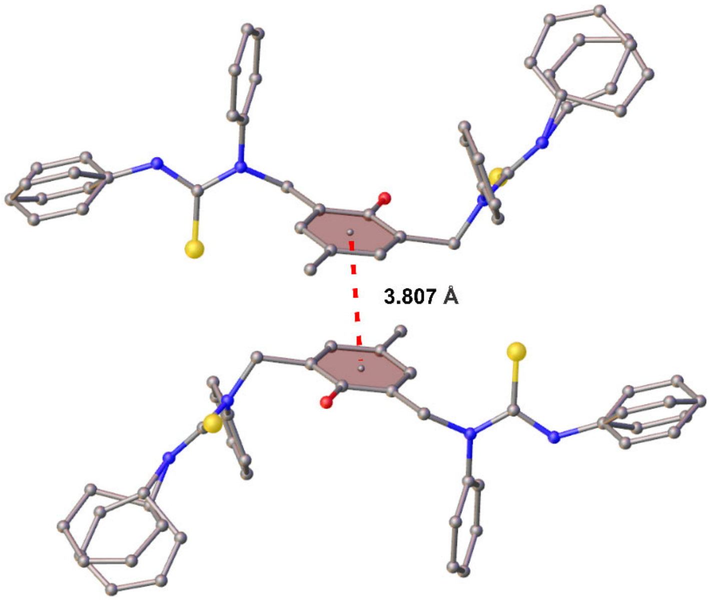

Figure S7. Visualization of the parallel displaced π... π interaction present in **4aa**.

Original NMR spectra

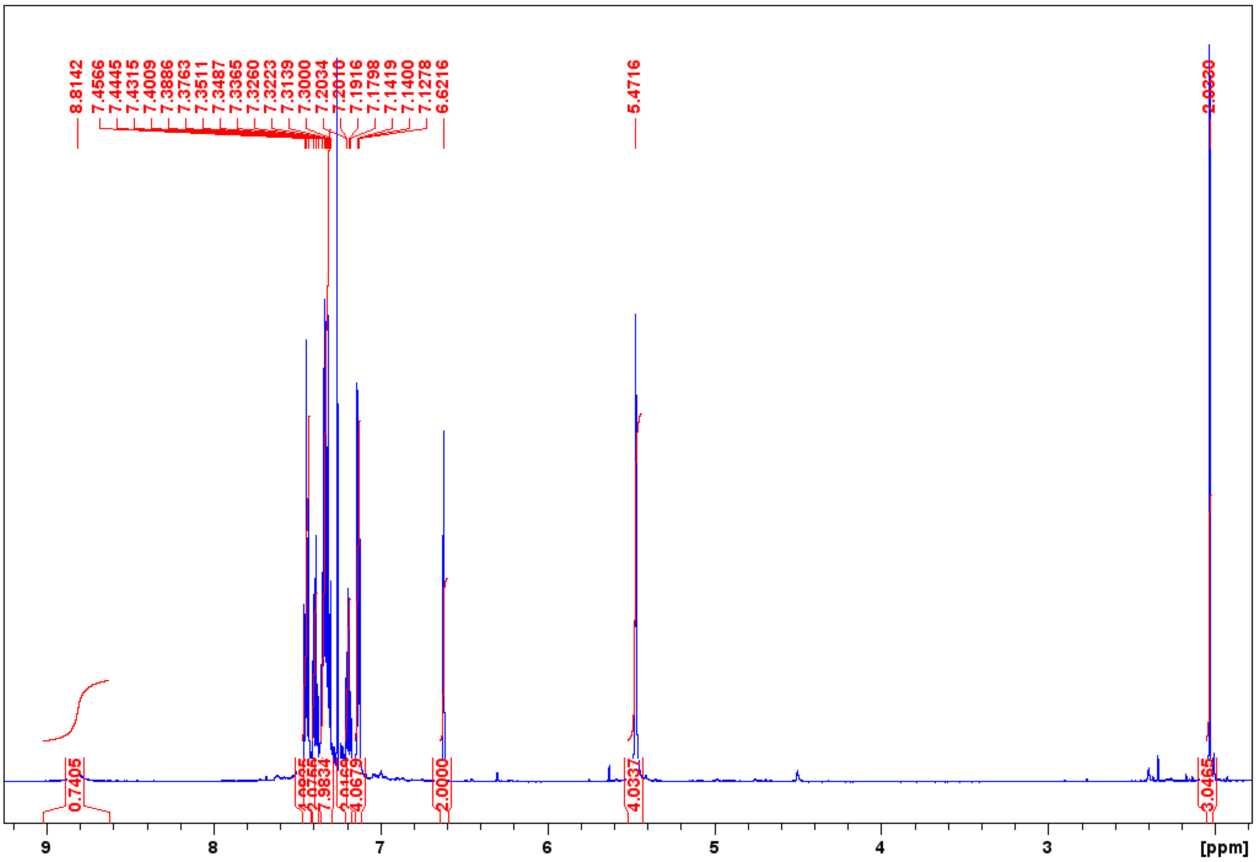

Figure S8. ^{1}H NMR spectrum of compound **4aa**.

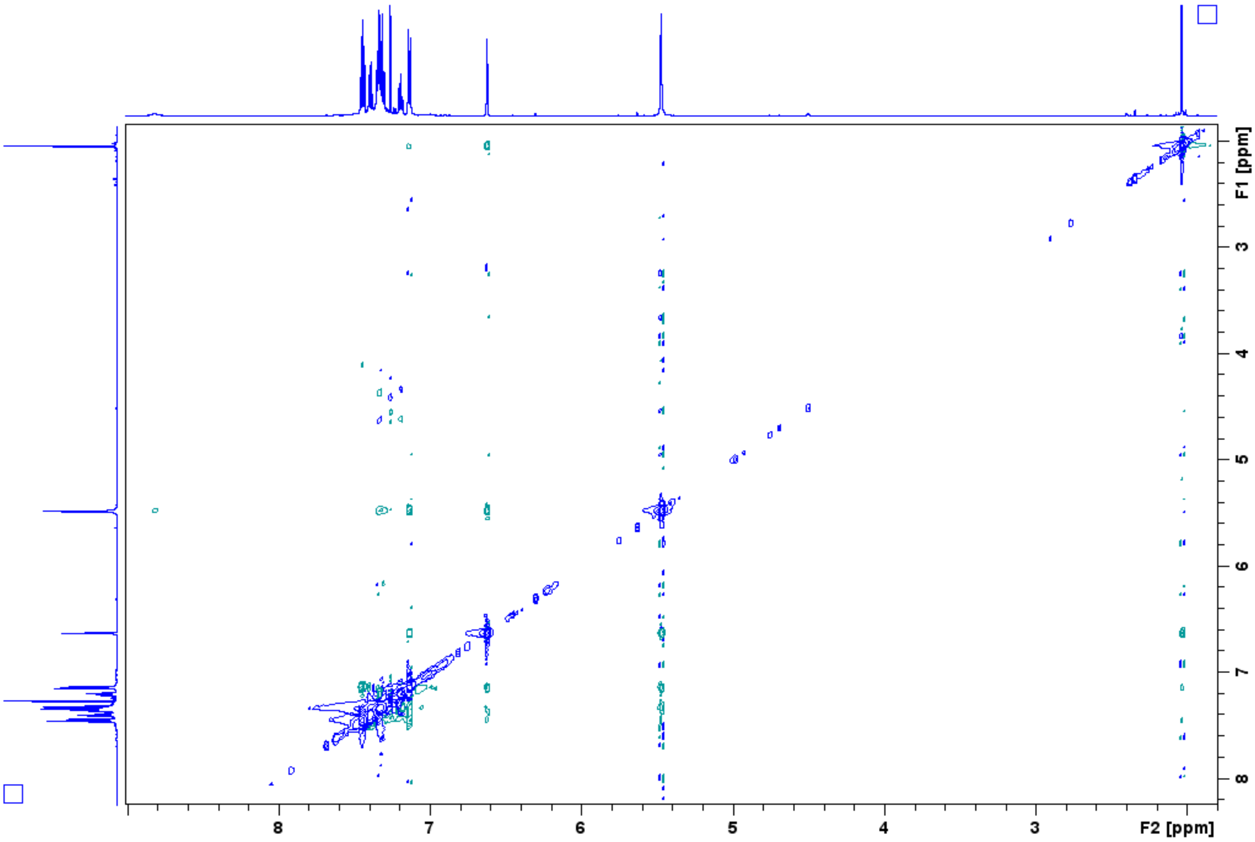

Figure S9. ^{1}H-^{1}H NOESY spectrum of compound **4aa**.

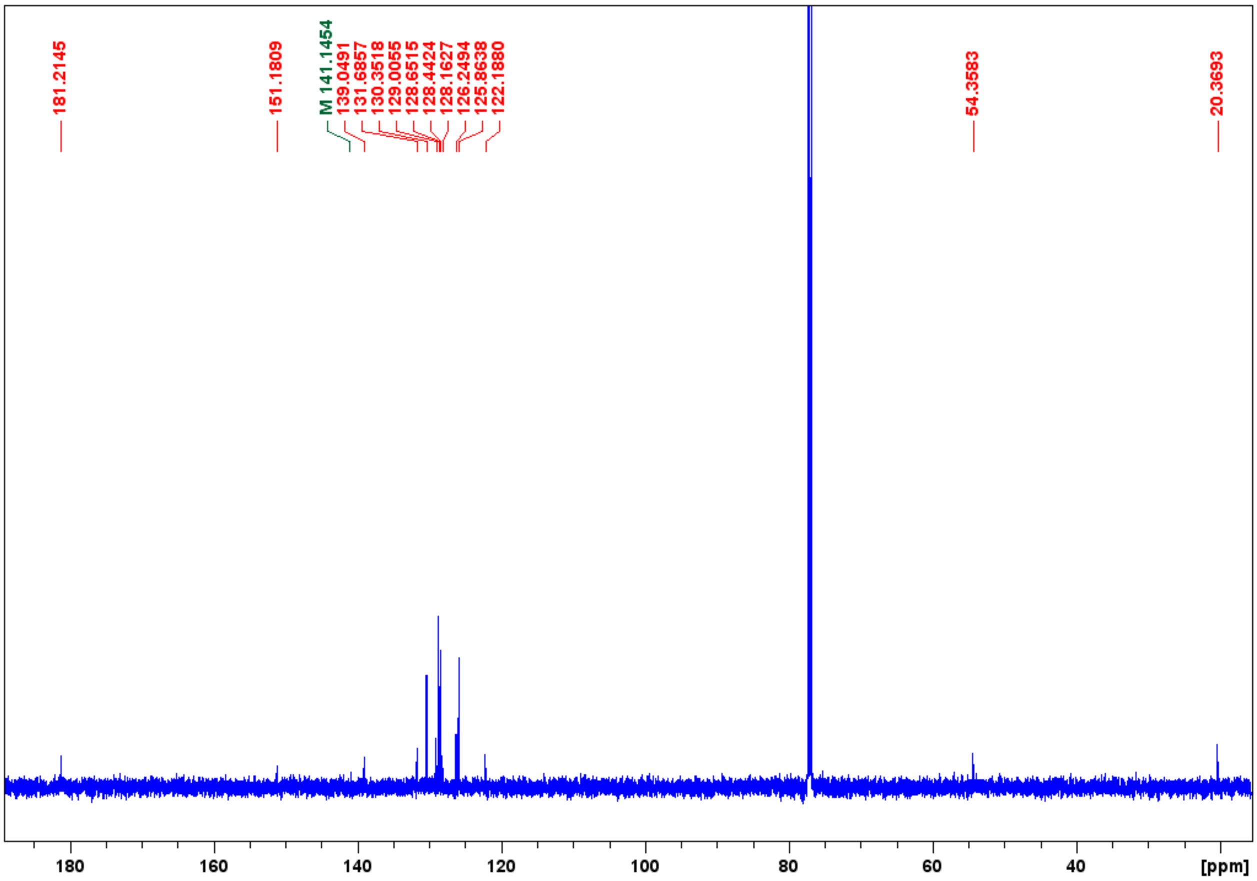

Figure S10. ^{13}C NMR spectrum of compound **4aa**.

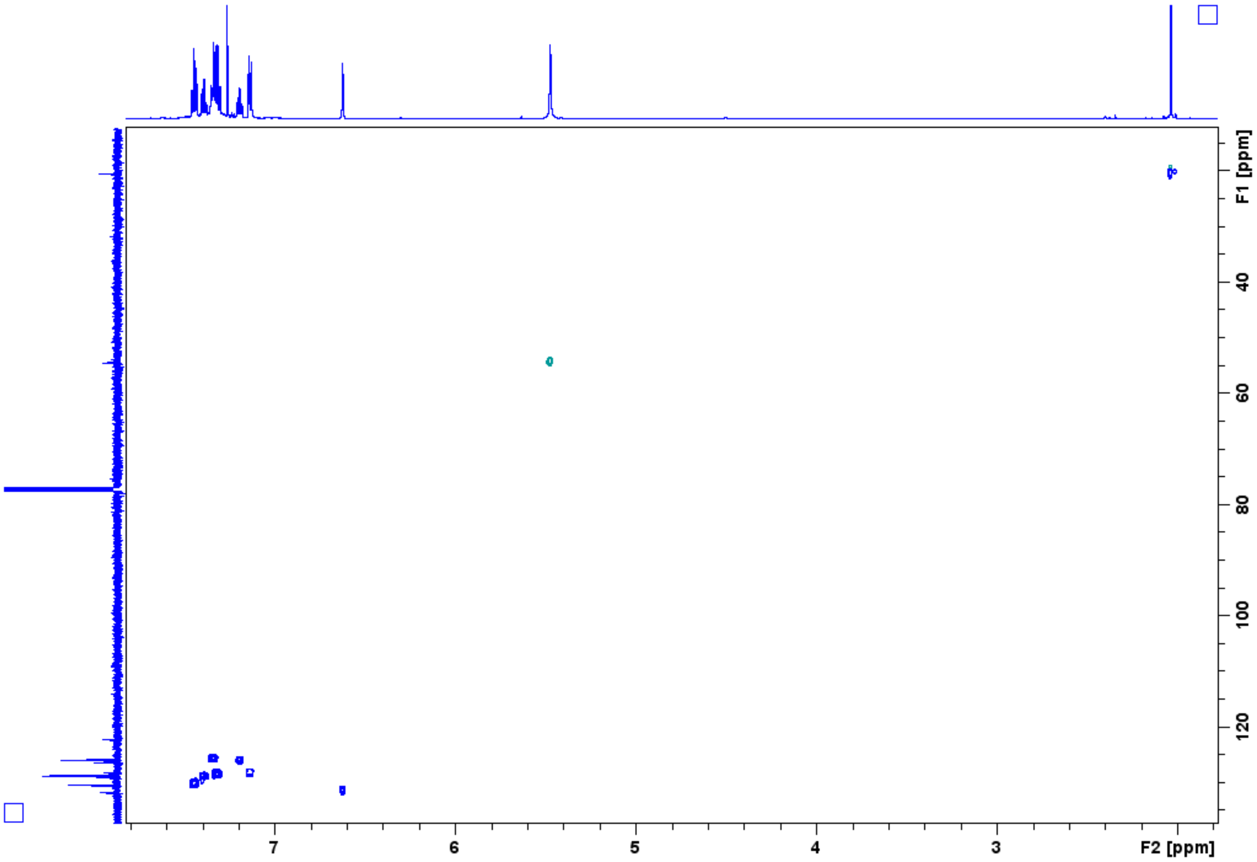

Figure S11. ^{1}H-^{13}C HSQC spectrum of compound **4aa**.

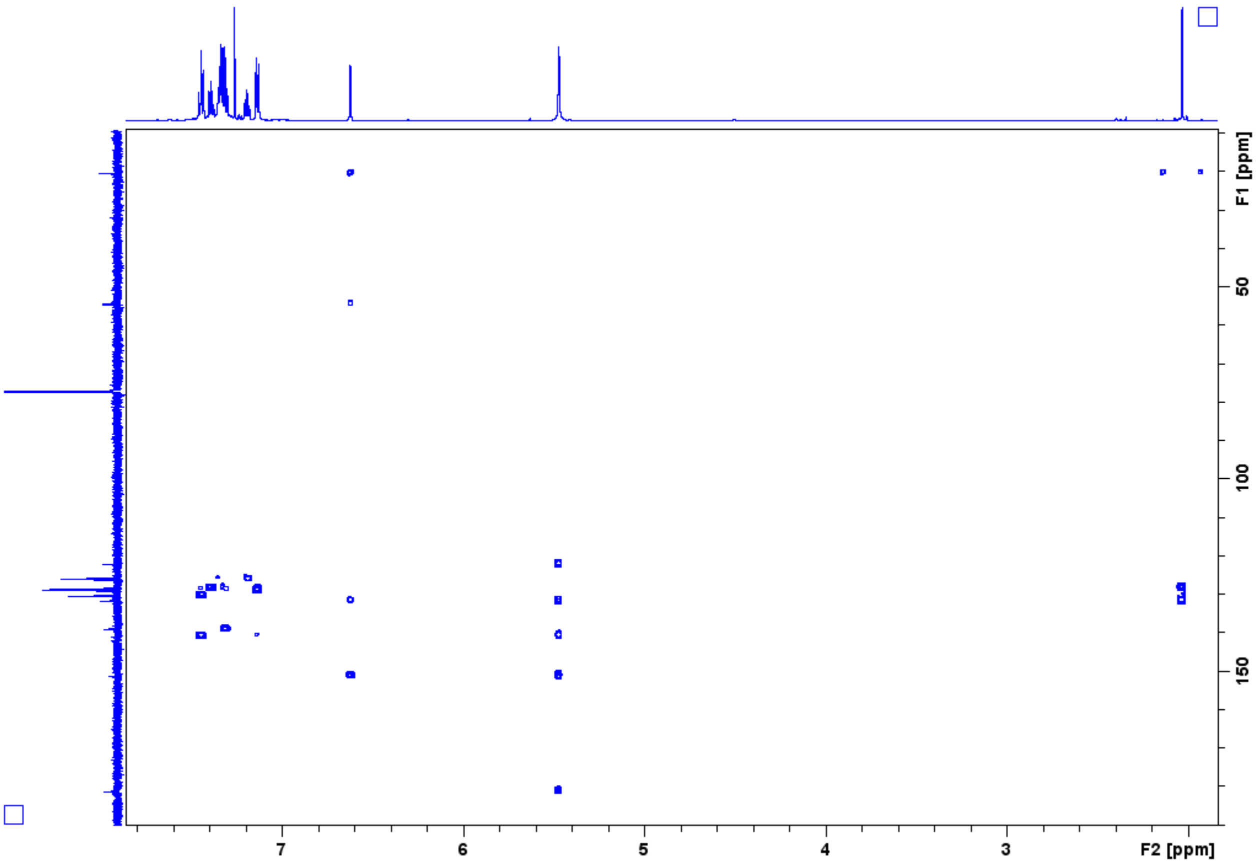

Figure S12. ^{1}H-^{13}C HMBC spectrum of compound **4aa**.

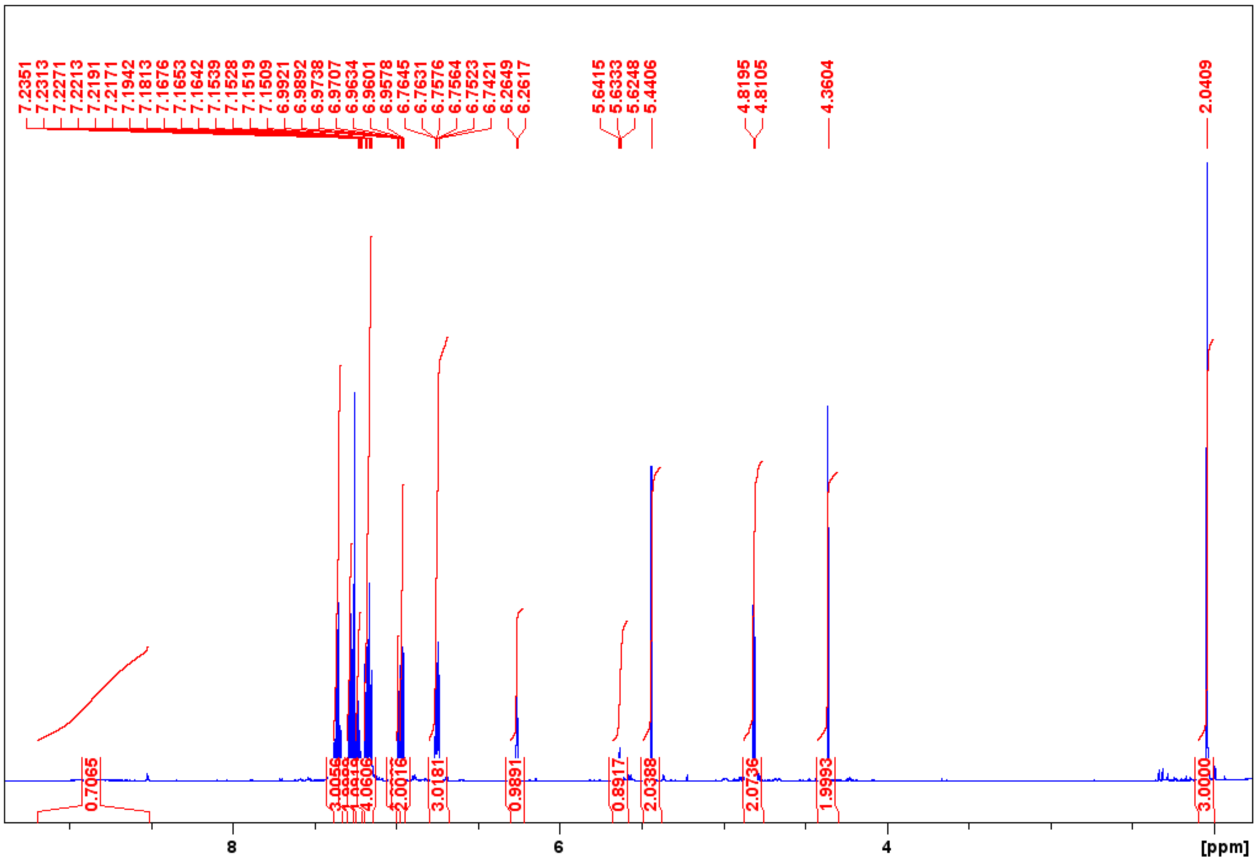

Figure S13. ^{1}H NMR spectrum of compound **3ab**.

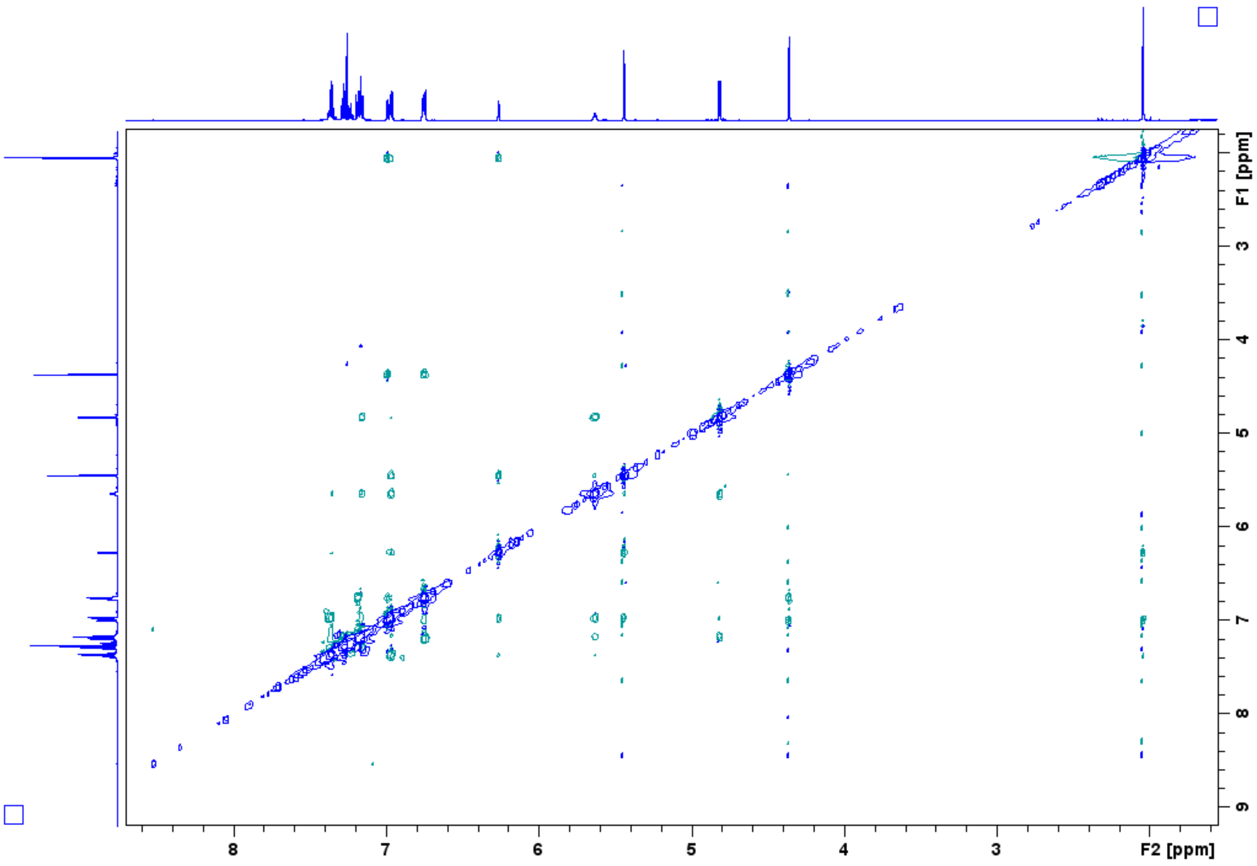

Figure S14. ^{1}H-^{1}H NOESY spectrum of compound **3ab**.

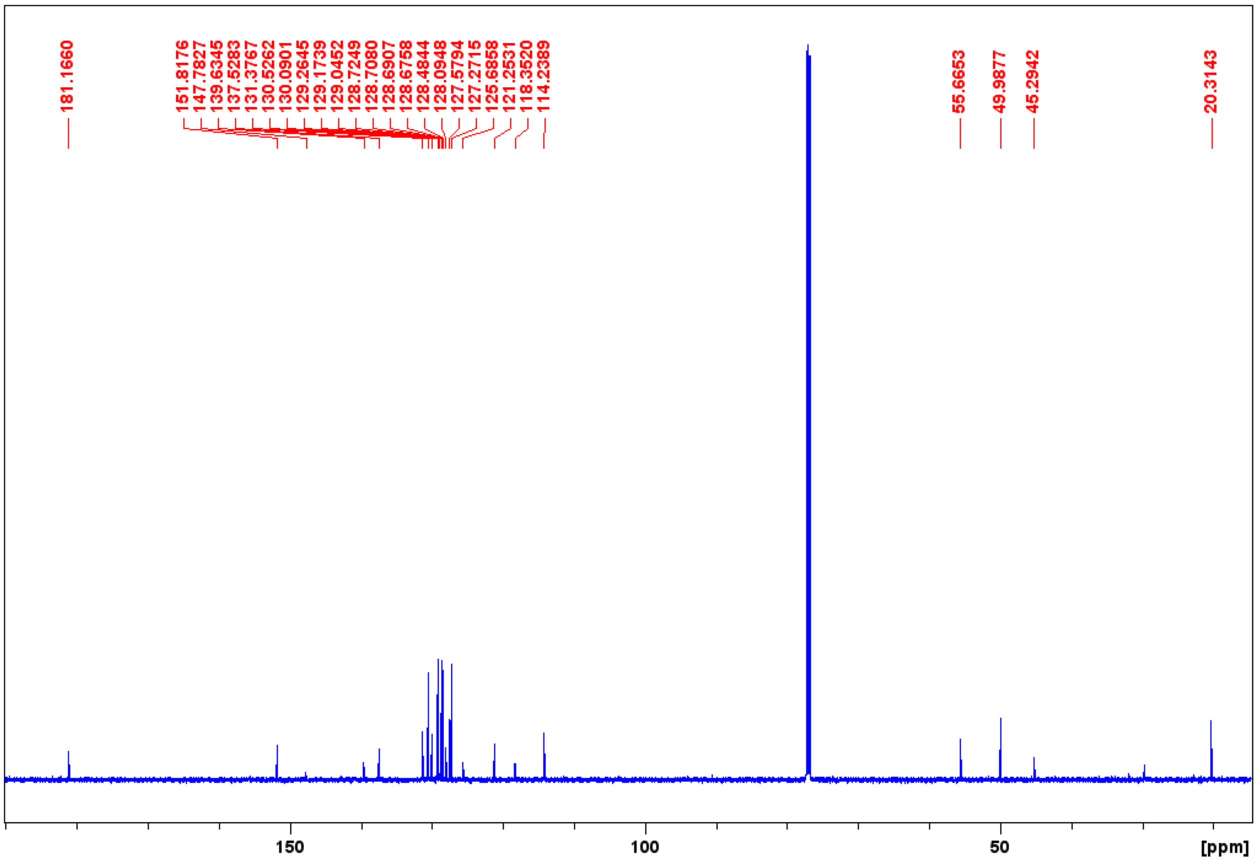

Figure S15. ^{13}C NMR spectrum of compound **3ab**.

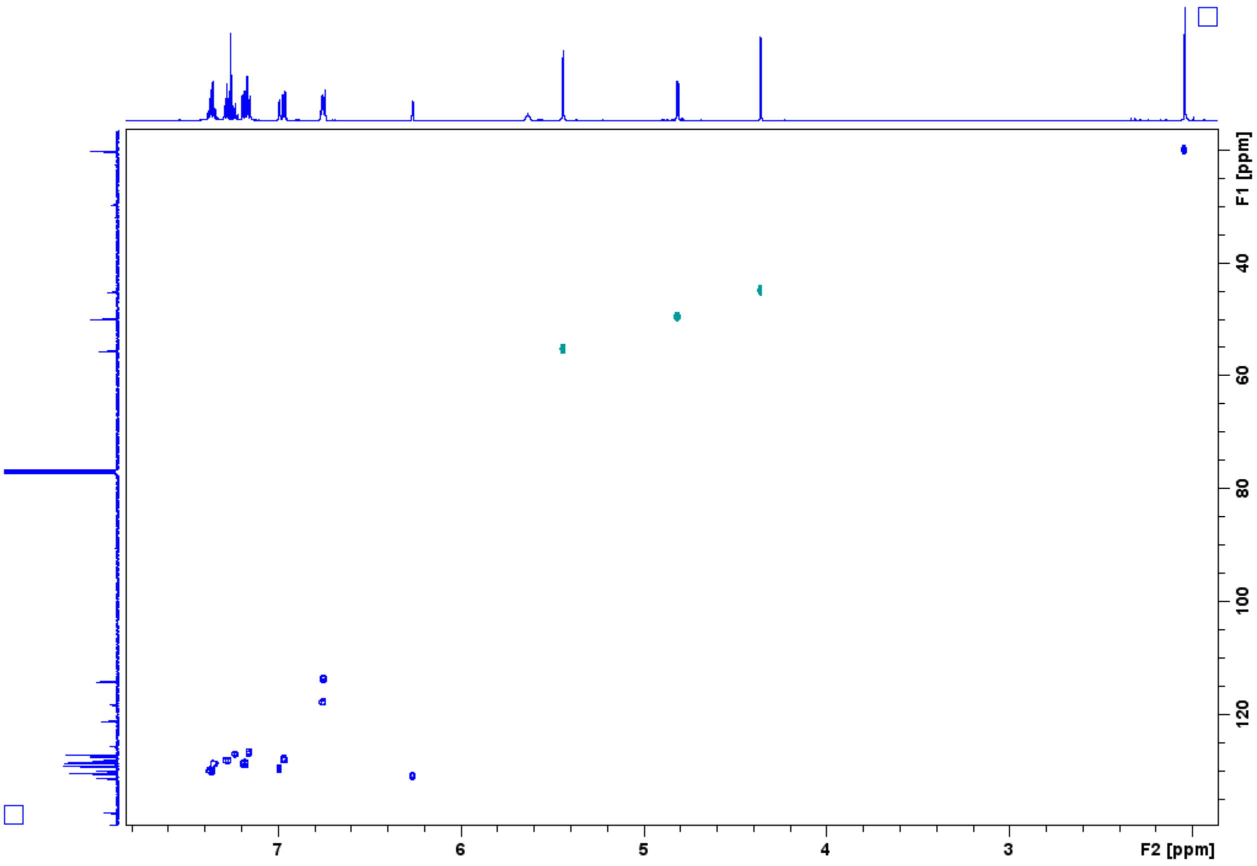

Figure S16. ^{1}H-^{13}C HSQC spectrum of compound **3ab**.

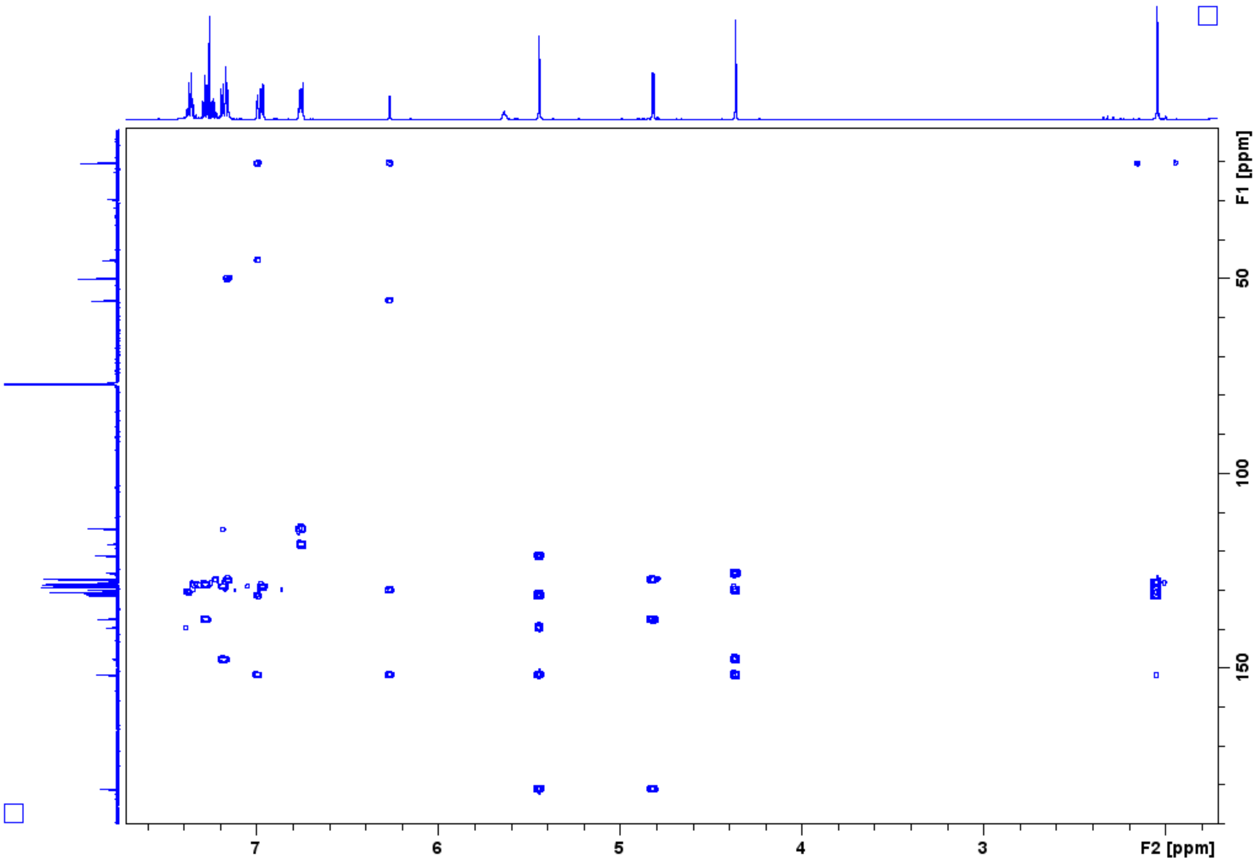

Figure S17. ^{1}H-^{13}C HMBC spectrum of compound **3ab**.

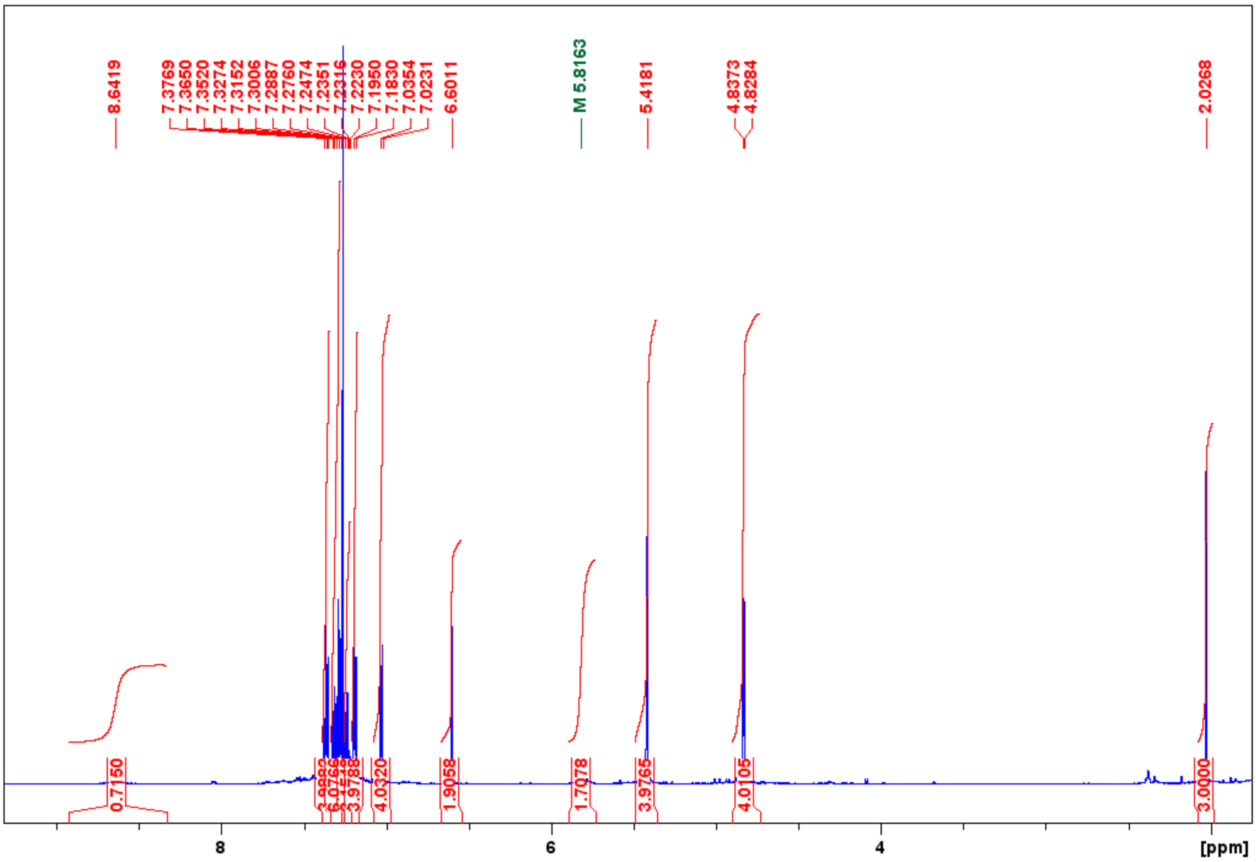

Figure S18. ^{1}H NMR spectrum of compound **4ab**.

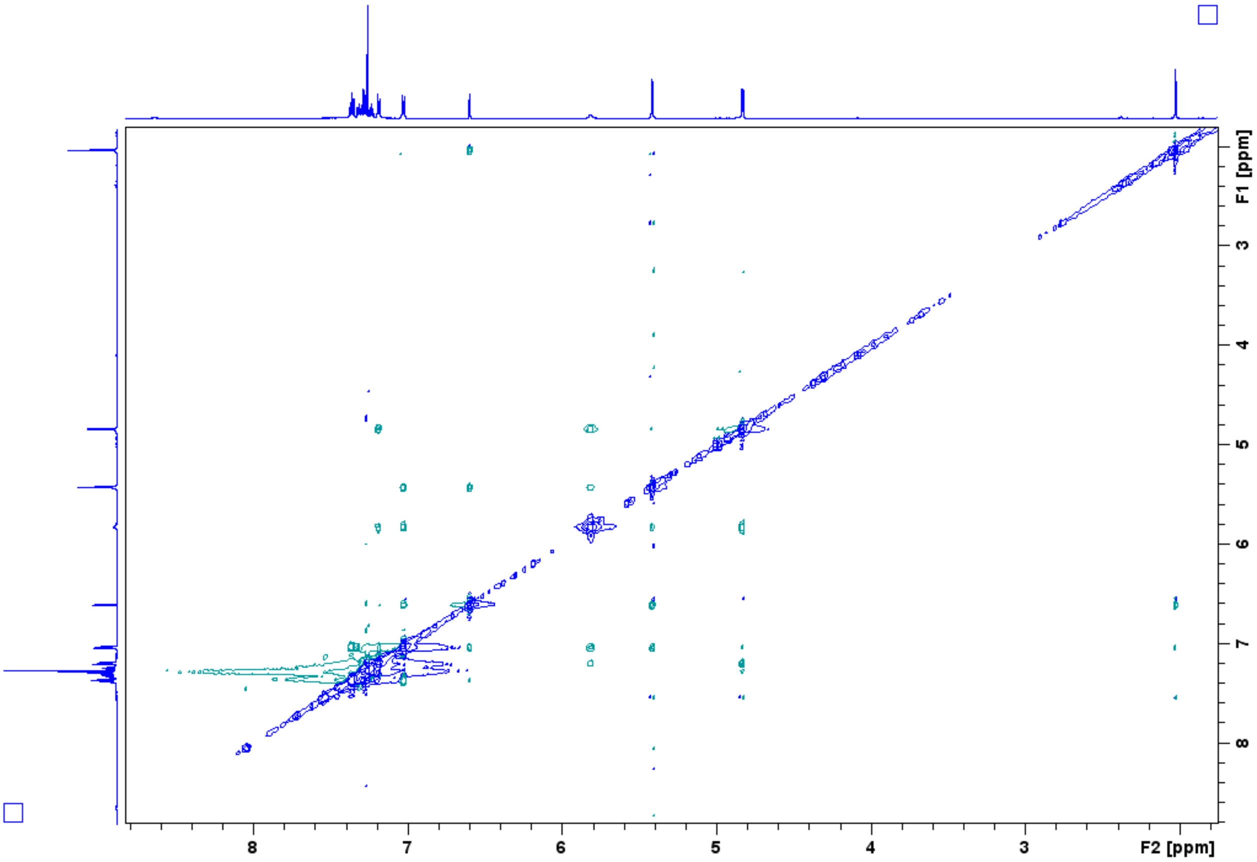

Figure S19. ^{1}H-^{1}H NOESY spectrum of compound **4ab**.

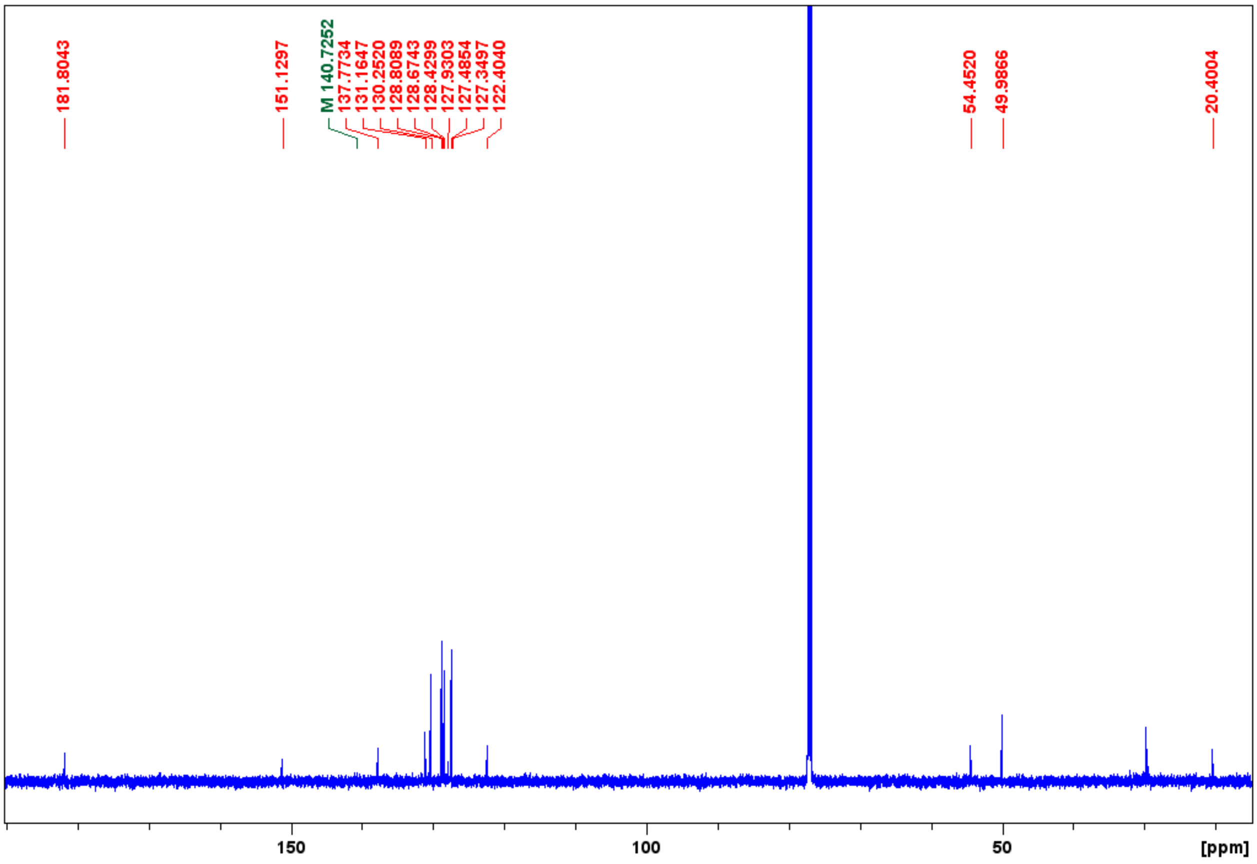

Figure S20. ^{13}C NMR spectrum of compound **4ab**.

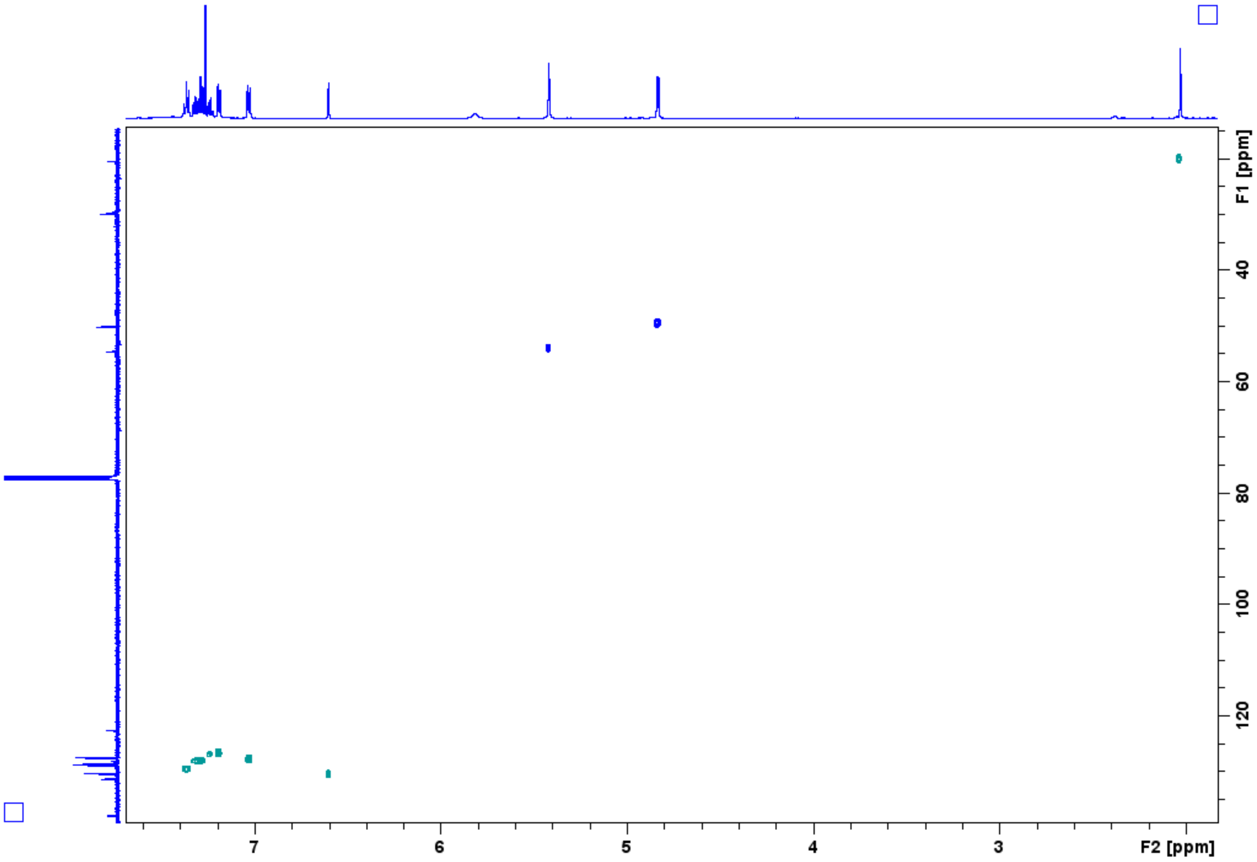

Figure S21. ^{1}H-^{13}C HSQC spectrum of compound **4ab**.

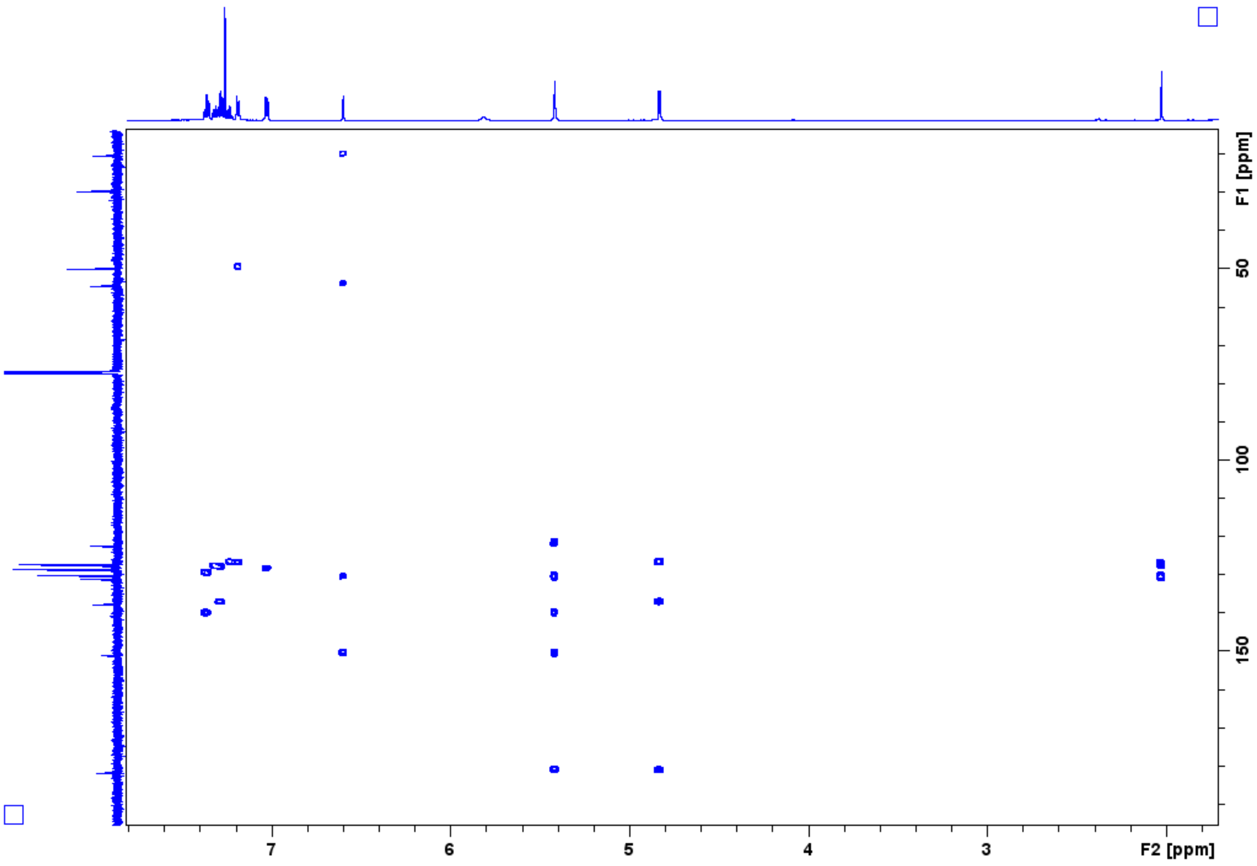

Figure S22. ^{1}H-^{13}C HMBC spectrum of compound **4ab**.

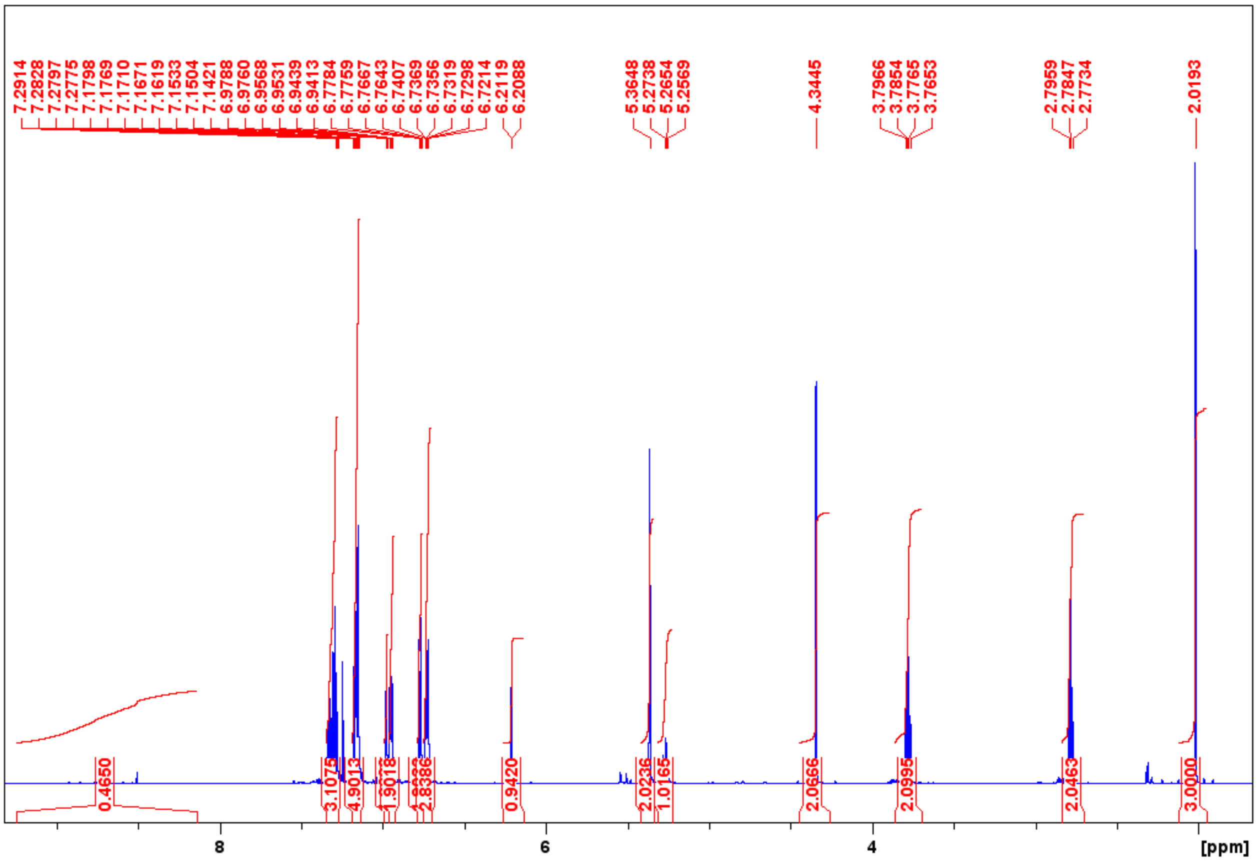

Figure S23. ^{1}H NMR spectrum of compound **3ac**.

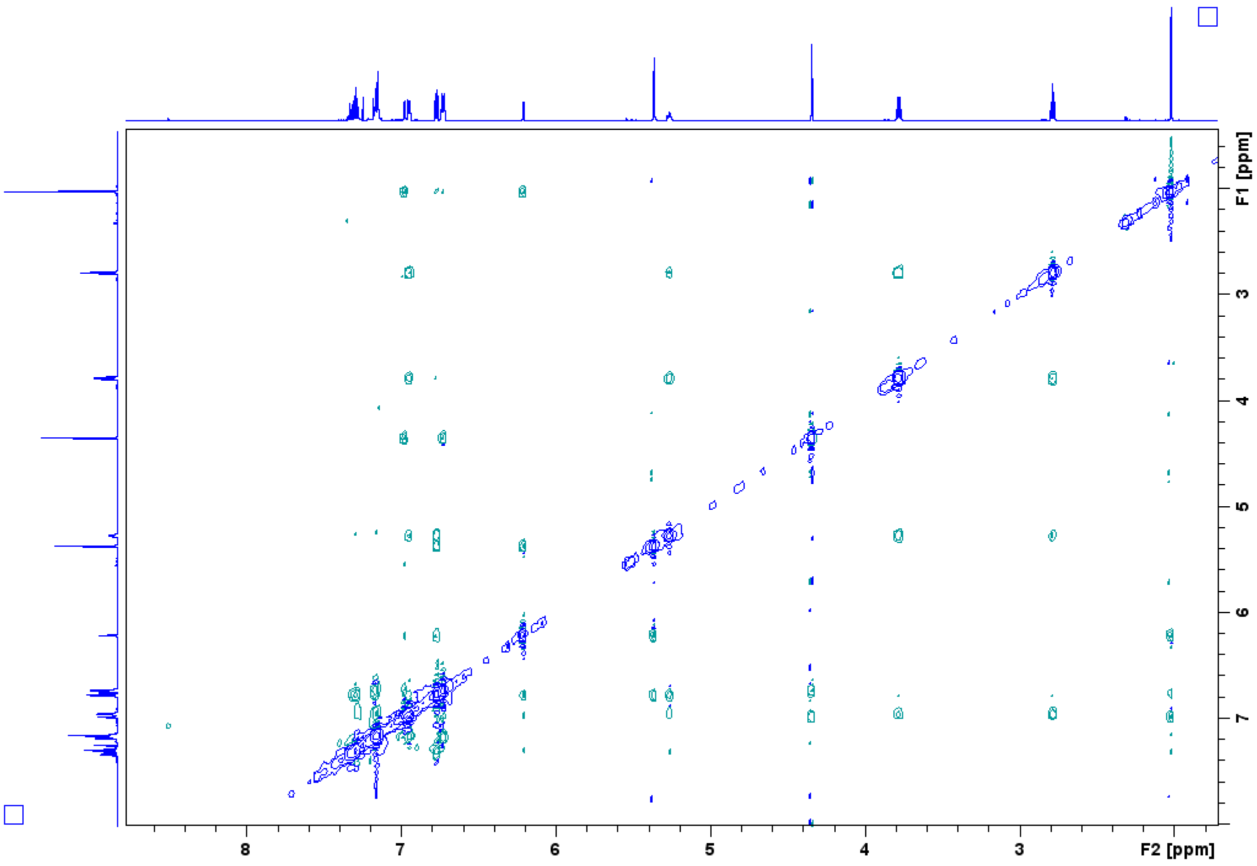

Figure S24. ^{1}H-^{1}H NOESY spectrum of compound **3ac**.

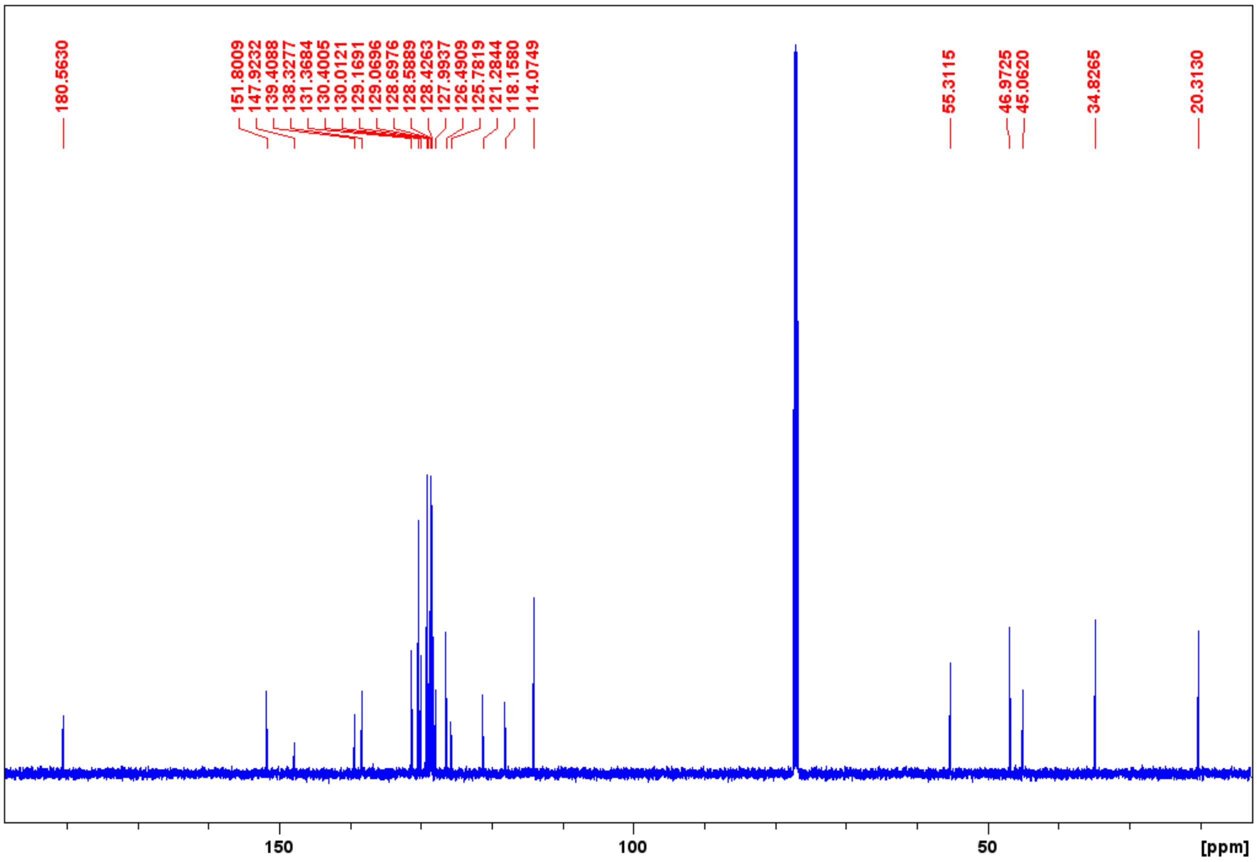

Figure S25. ^{13}C NMR spectrum of compound **3ac**.

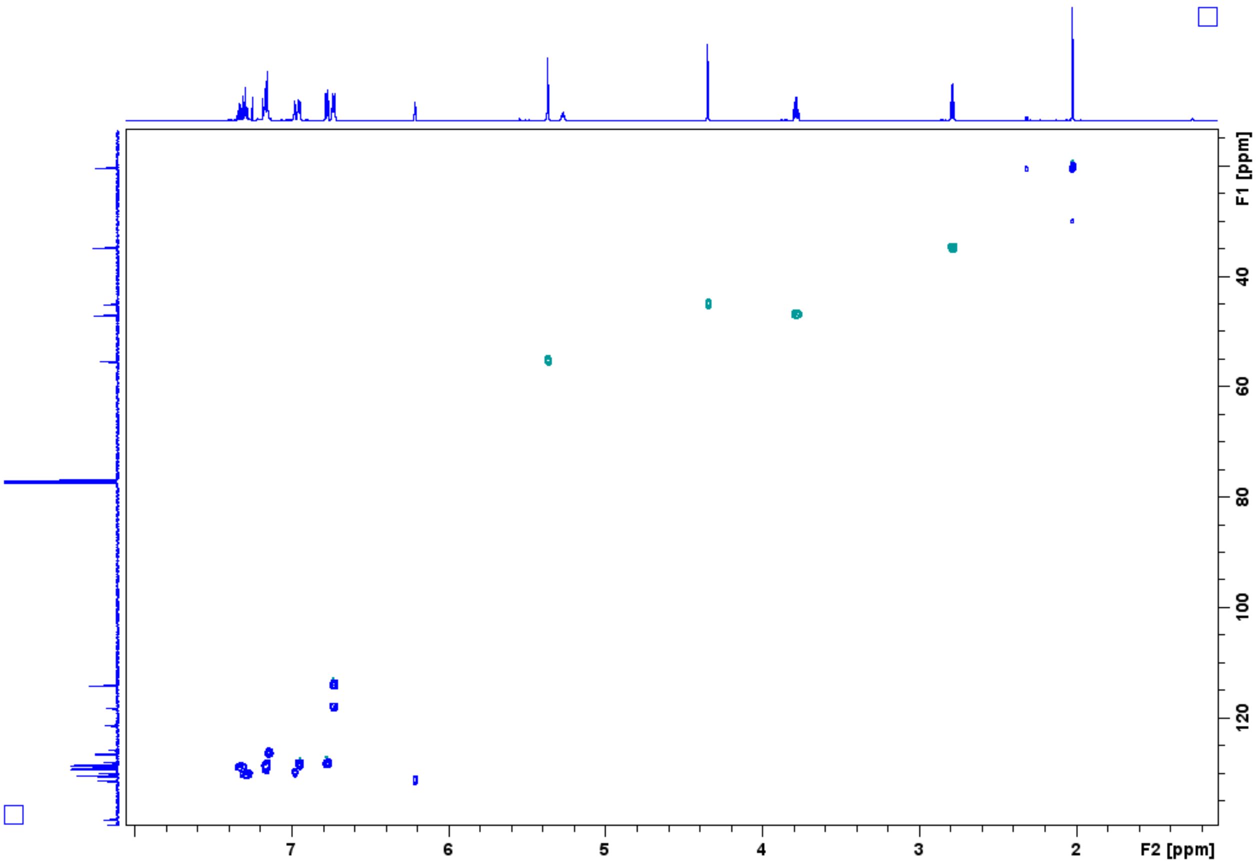

Figure S26. ^{1}H-^{13}C HSQC spectrum of compound **3ac**.

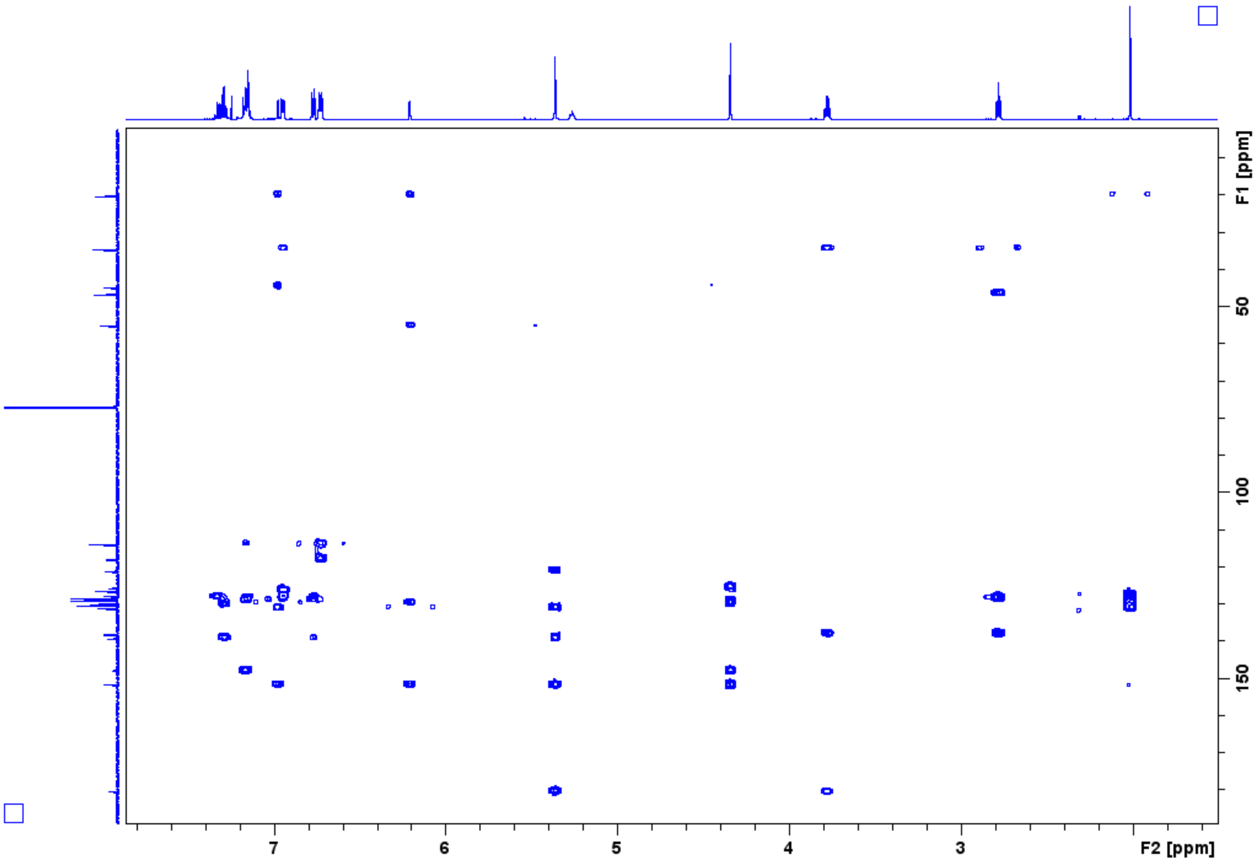

Figure S27. ^{1}H-^{13}C HMBC spectrum of compound **3ac**.

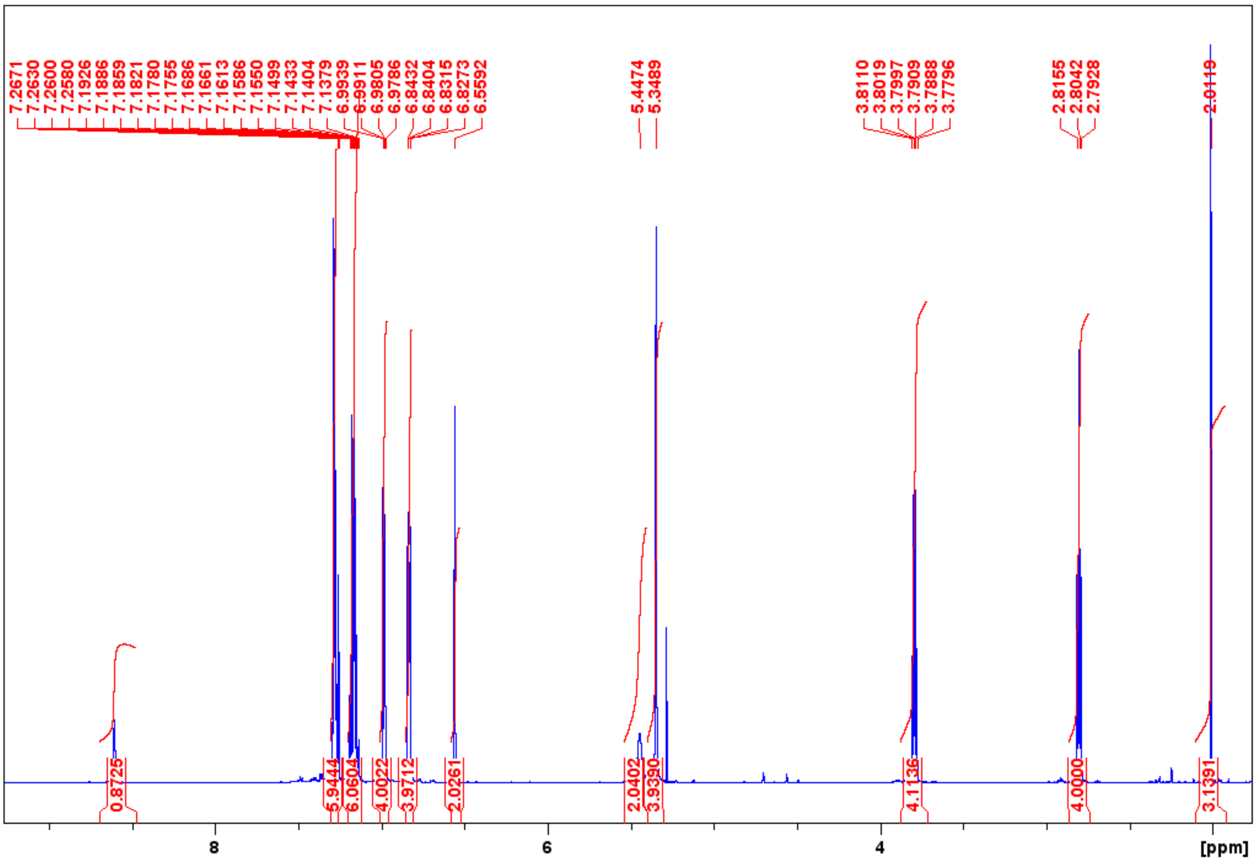

Figure S28. ^{1}H NMR spectrum of compound **4ac**.

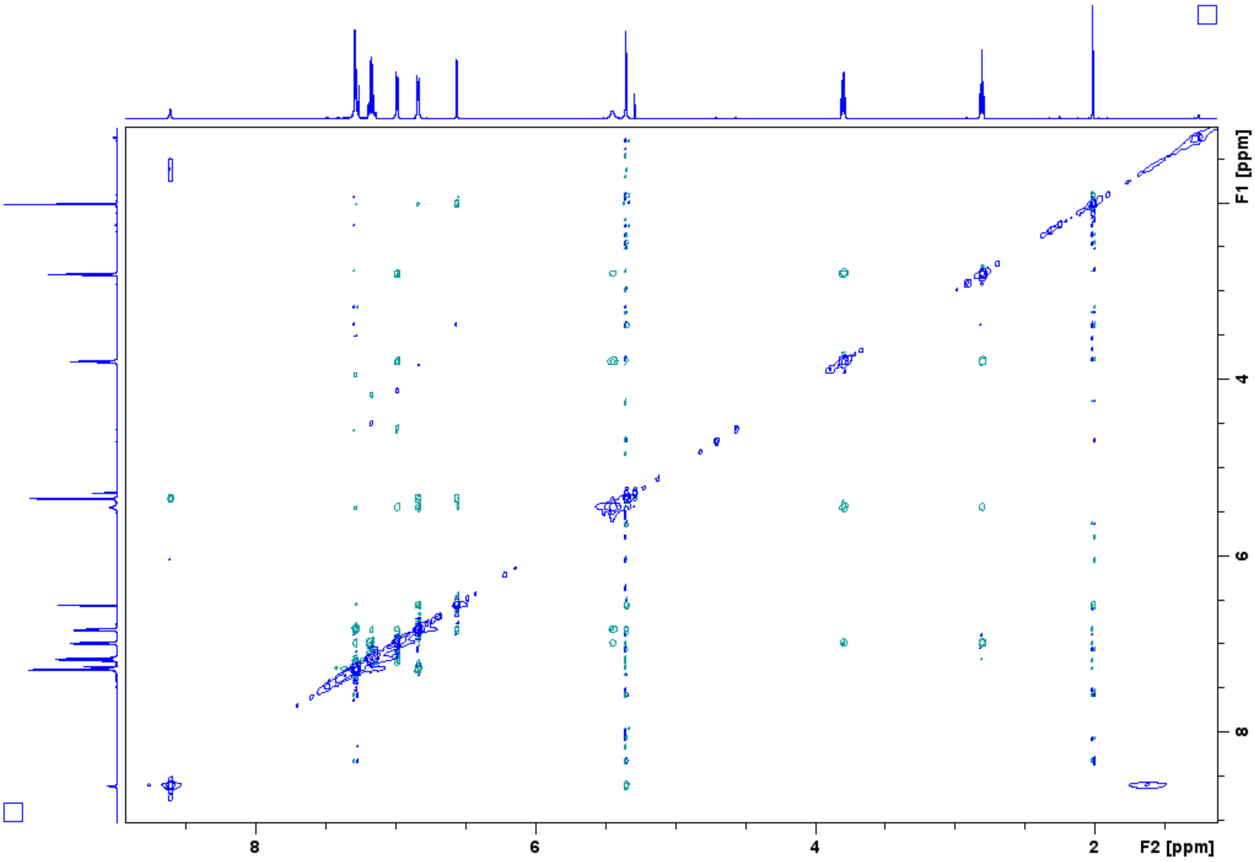

Figure S29. ^{1}H-^{1}H NOESY spectrum of compound **4ac**.

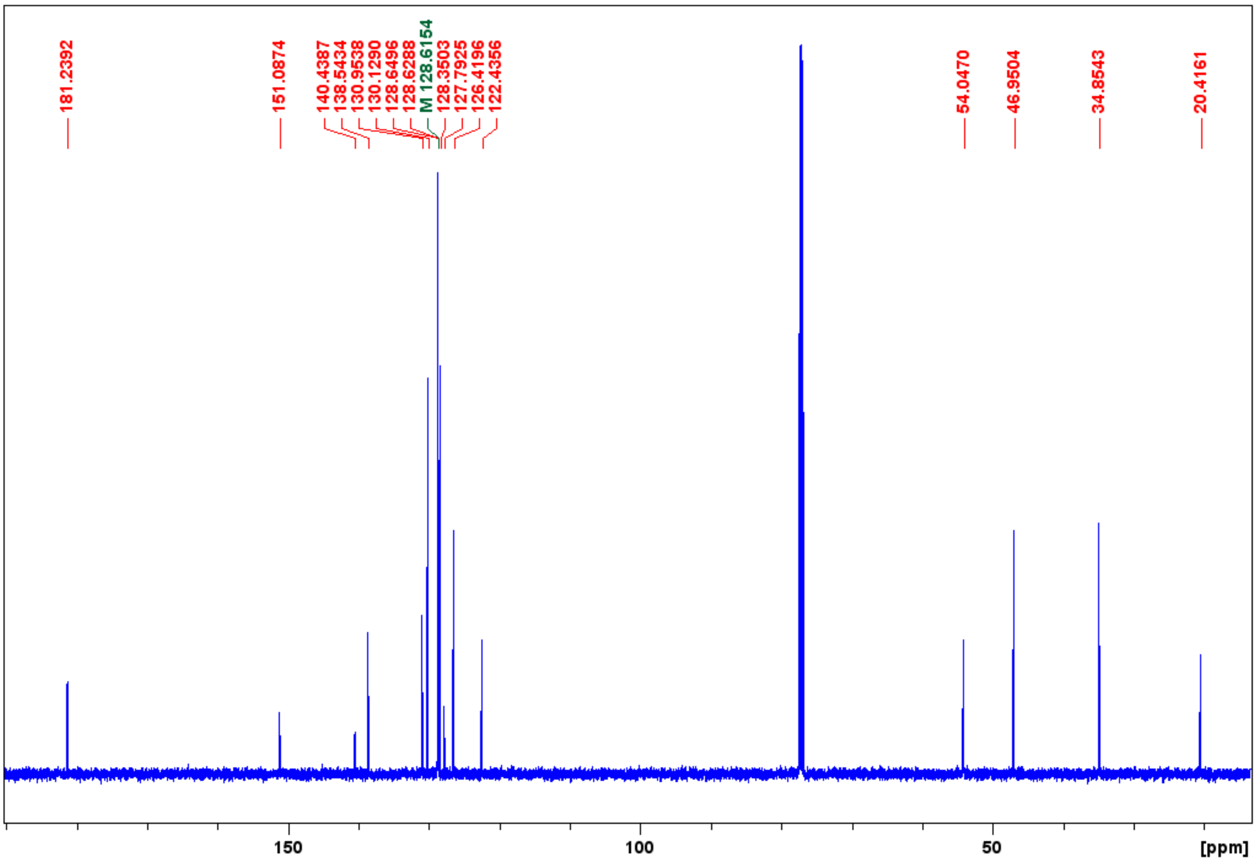

Figure S30. ^{13}C NMR spectrum of compound **4ac**.

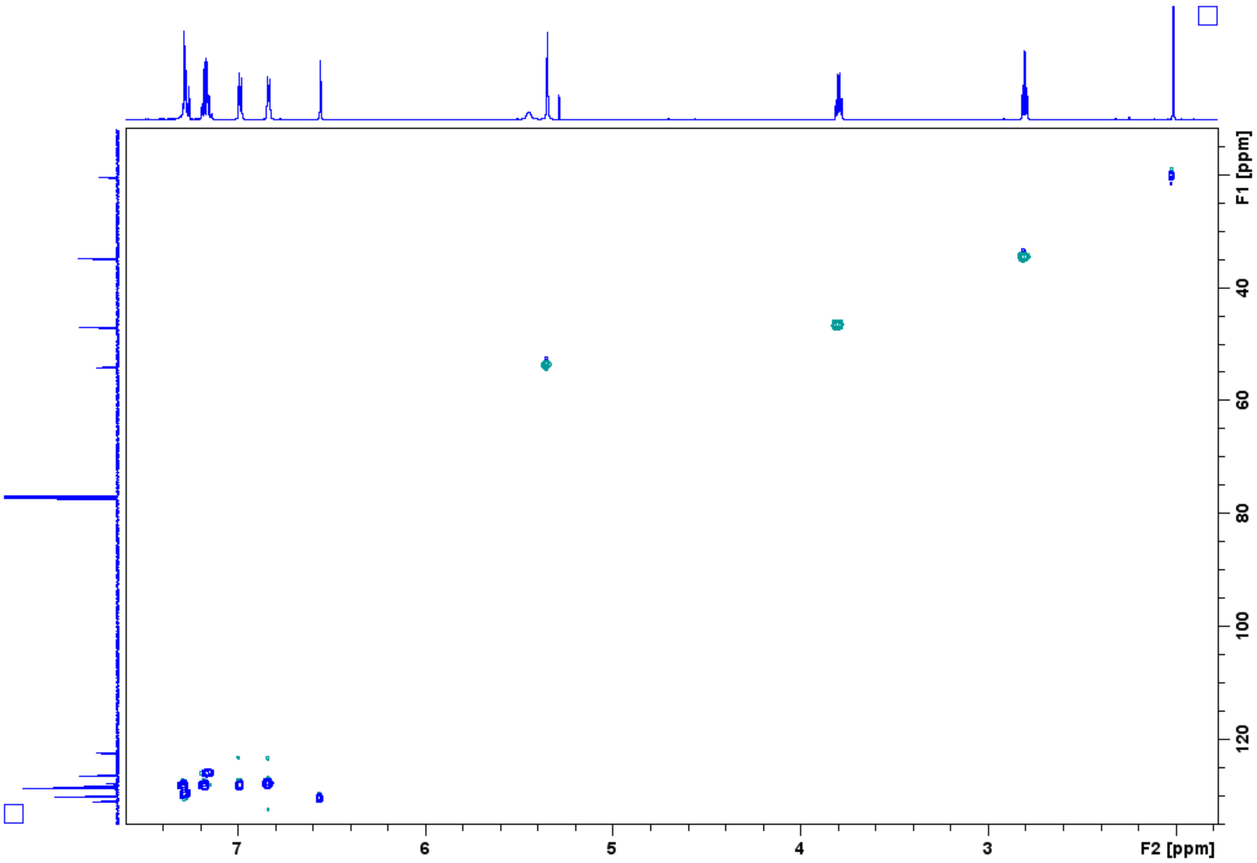

Figure S31. ^{1}H-^{13}C HSQC spectrum of compound **4ac**.

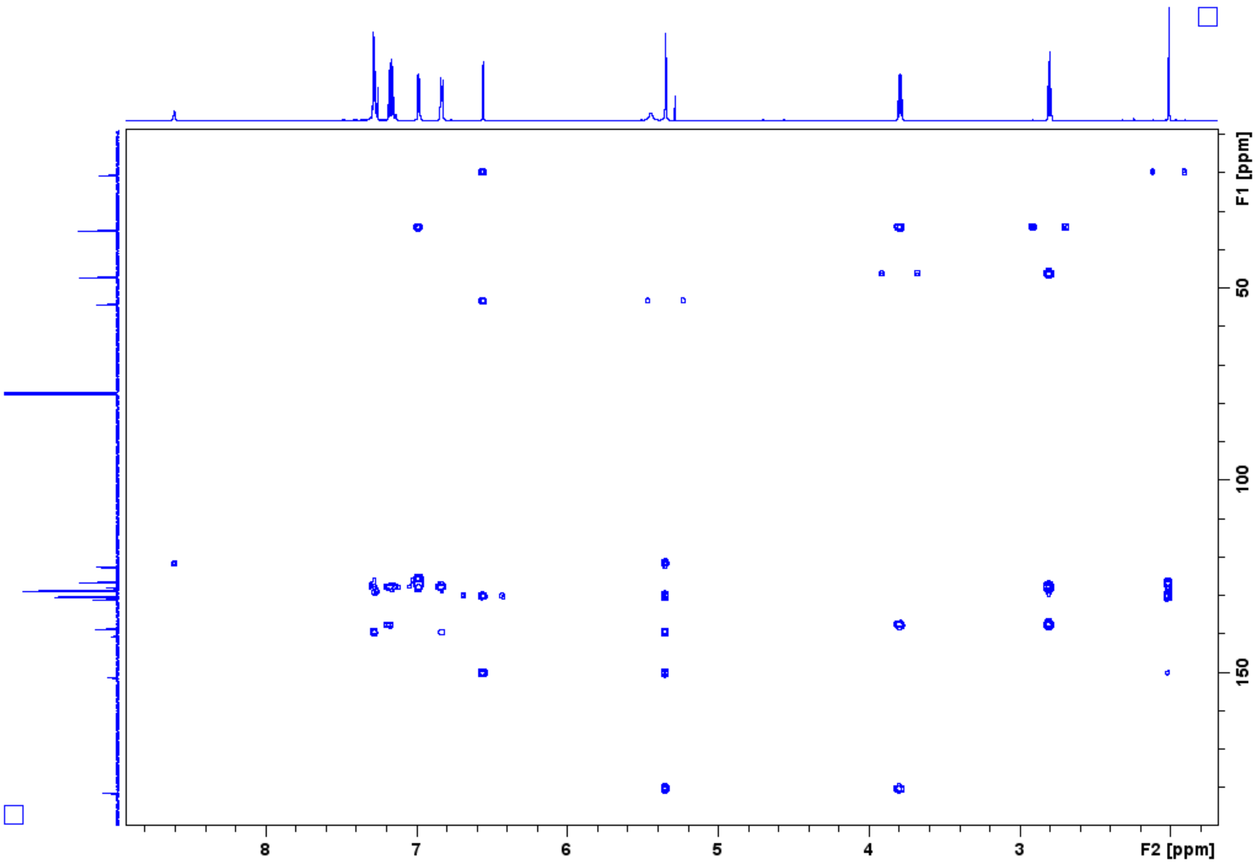

Figure S32. ^{1}H-^{13}C HMBC spectrum of compound **4ac**.

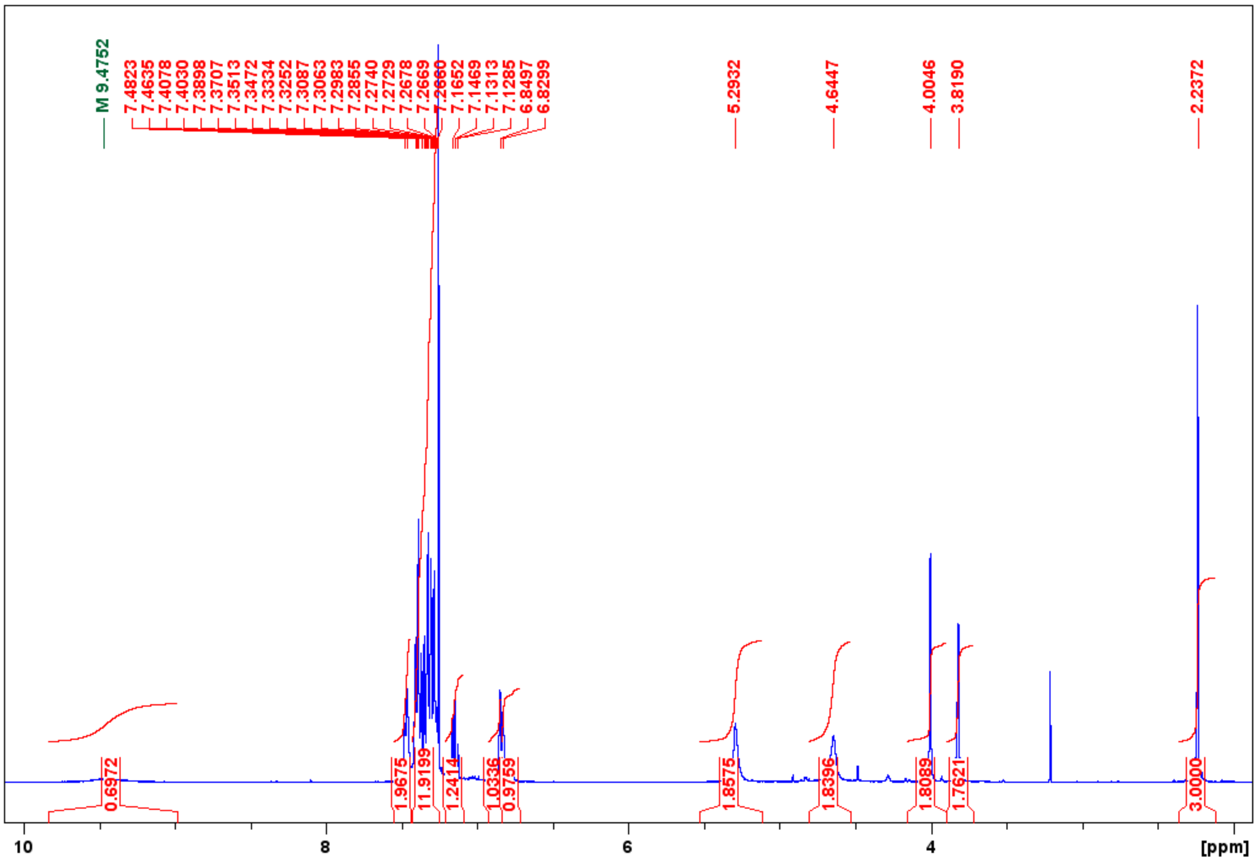

Figure S33. ^{1}H NMR spectrum of compound **3ba**.

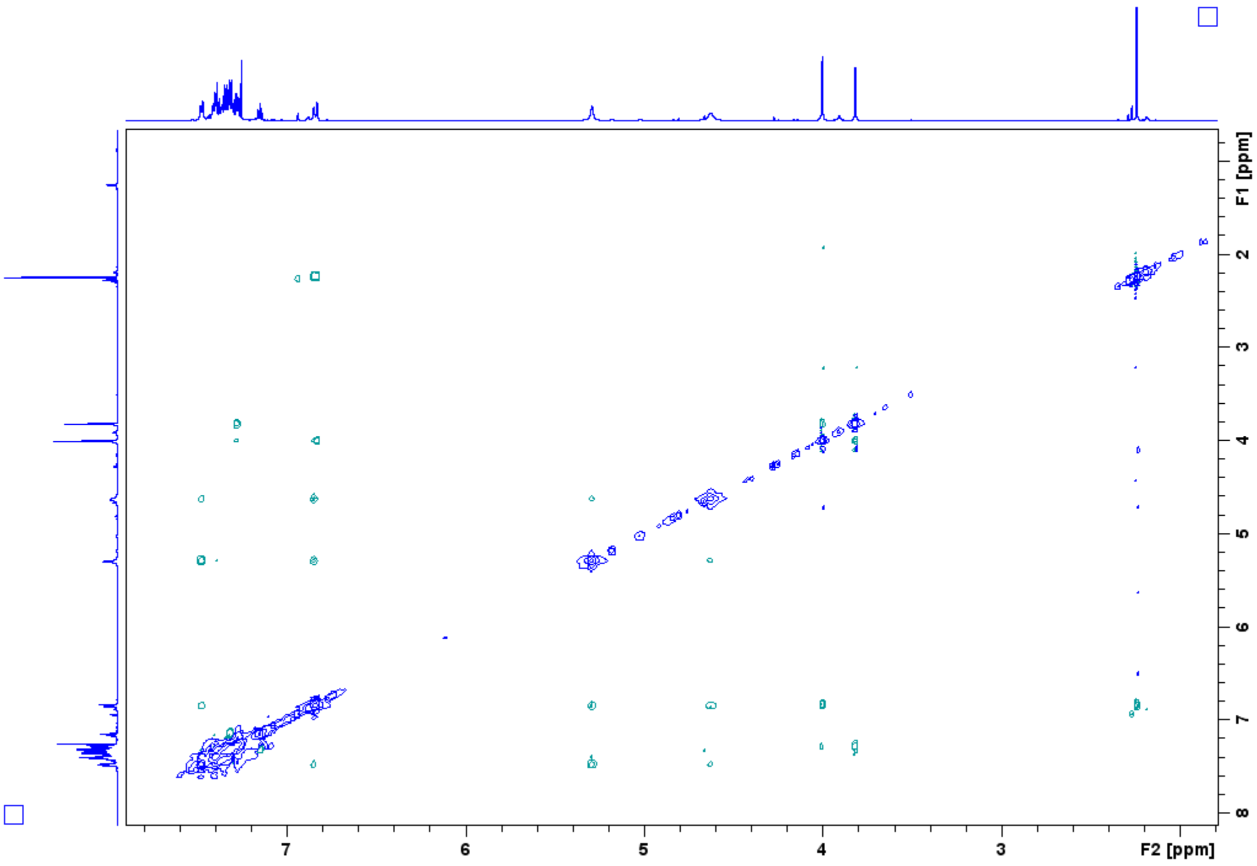

Figure S34. ^{1}H-^{1}H NOESY spectrum of compound **3ba**.

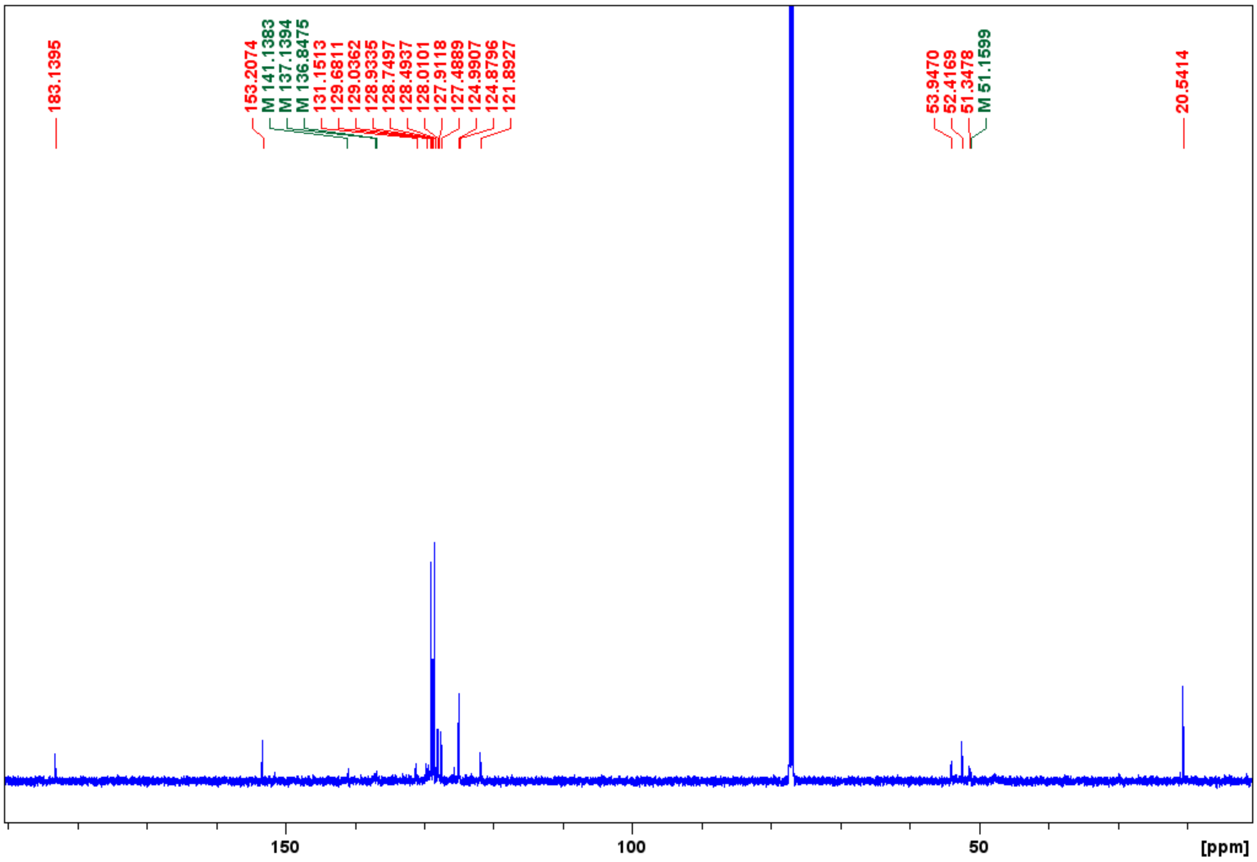

Figure S35. ^{13}C NMR spectrum of compound **3ba**.

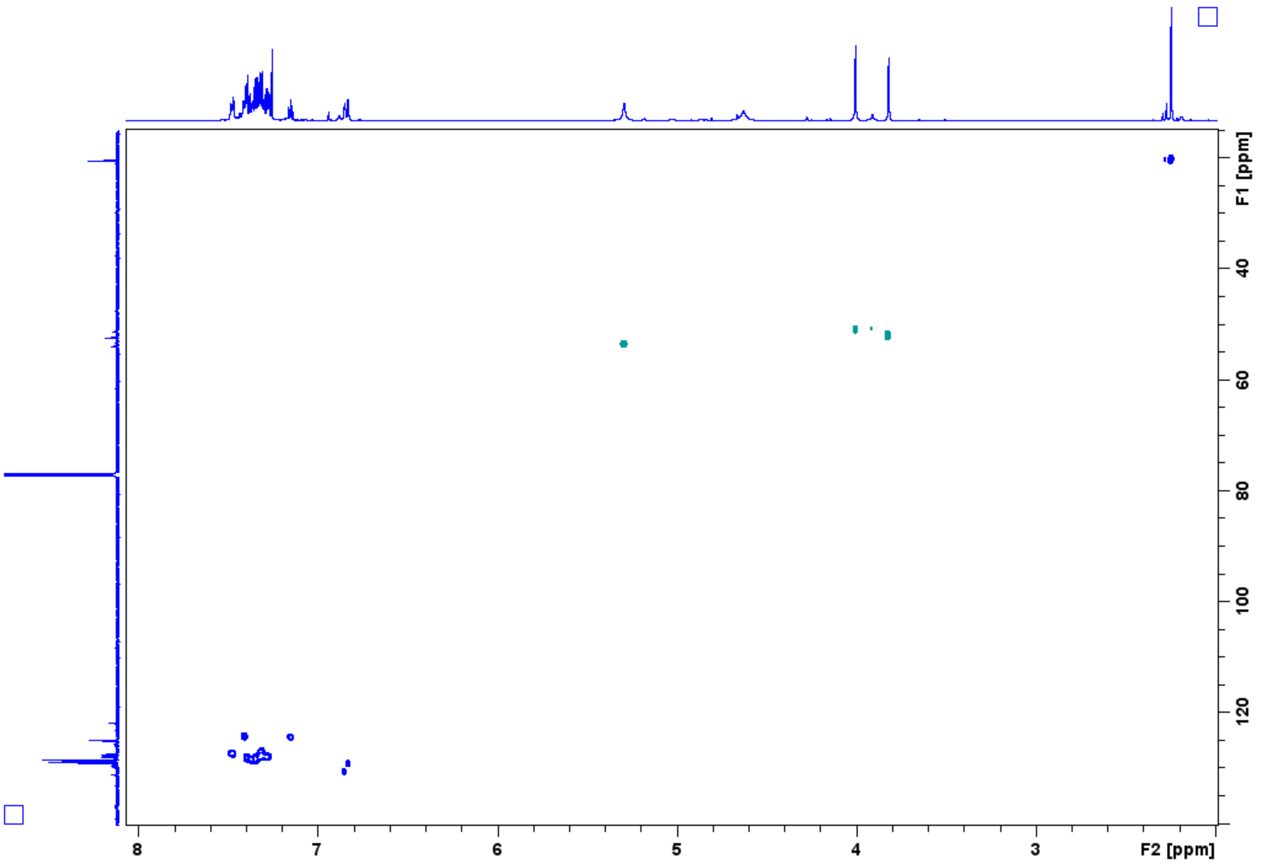

Figure S36. ^{1}H-^{13}C HSQC spectrum of compound **3ba**.

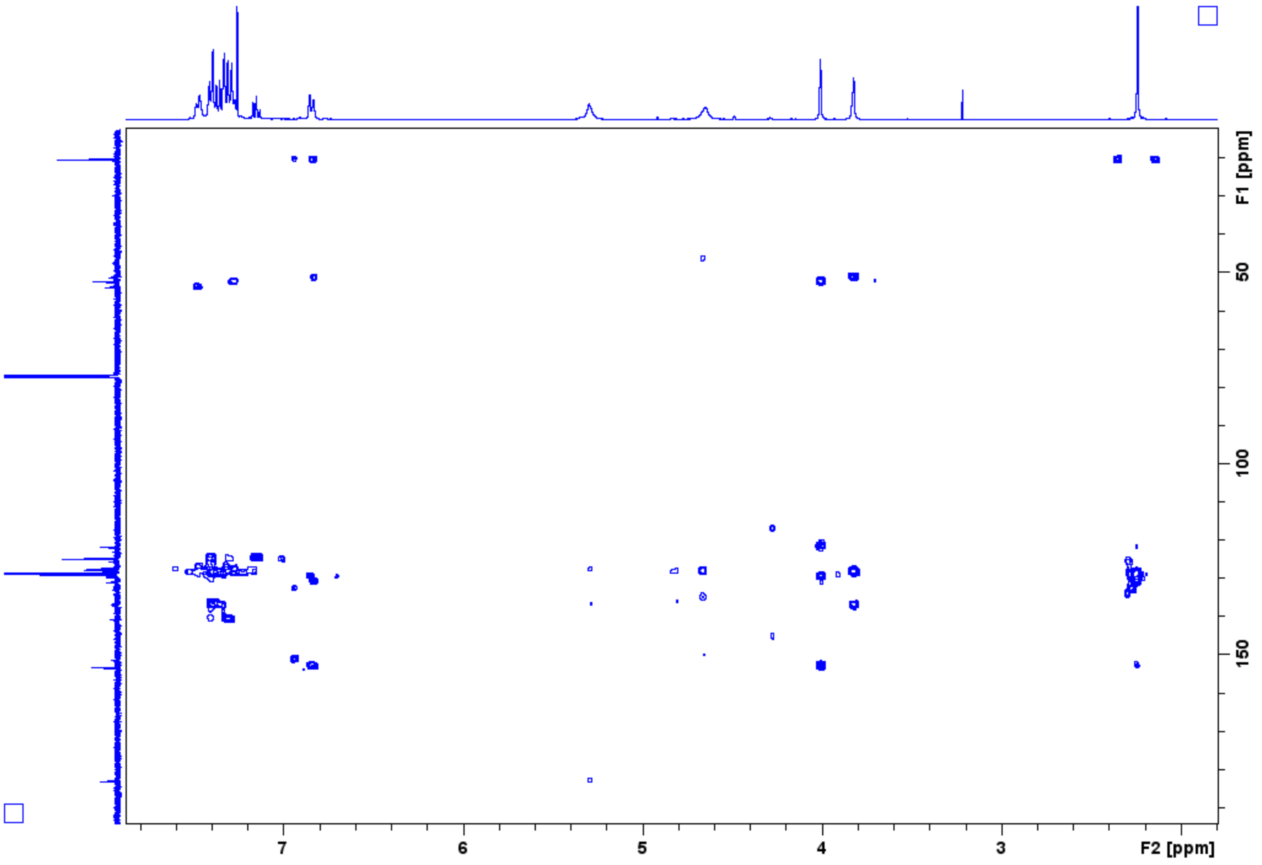

Figure S37. ^{1}H-^{13}C HMBC spectrum of compound **3ba**.

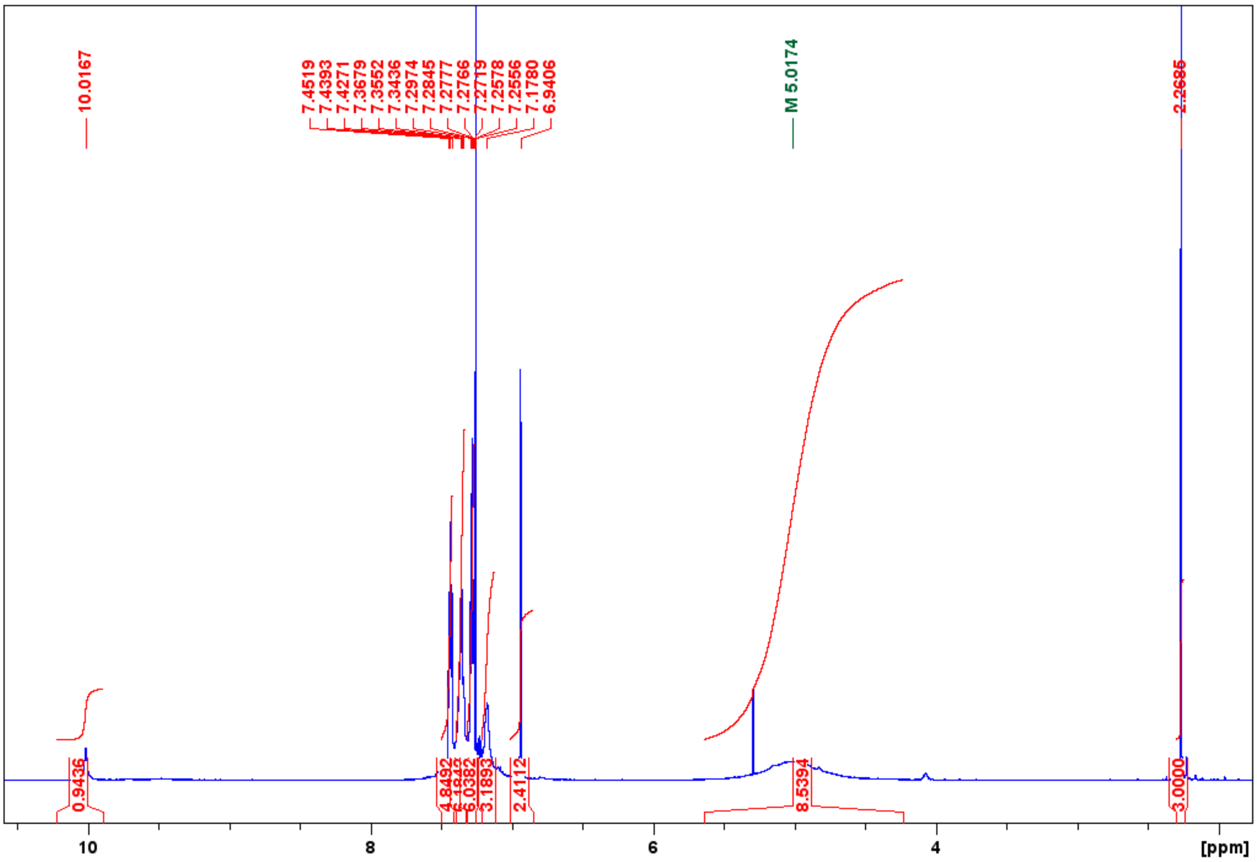

Figure S38. ^{1}H NMR spectrum of compound **4ba**.

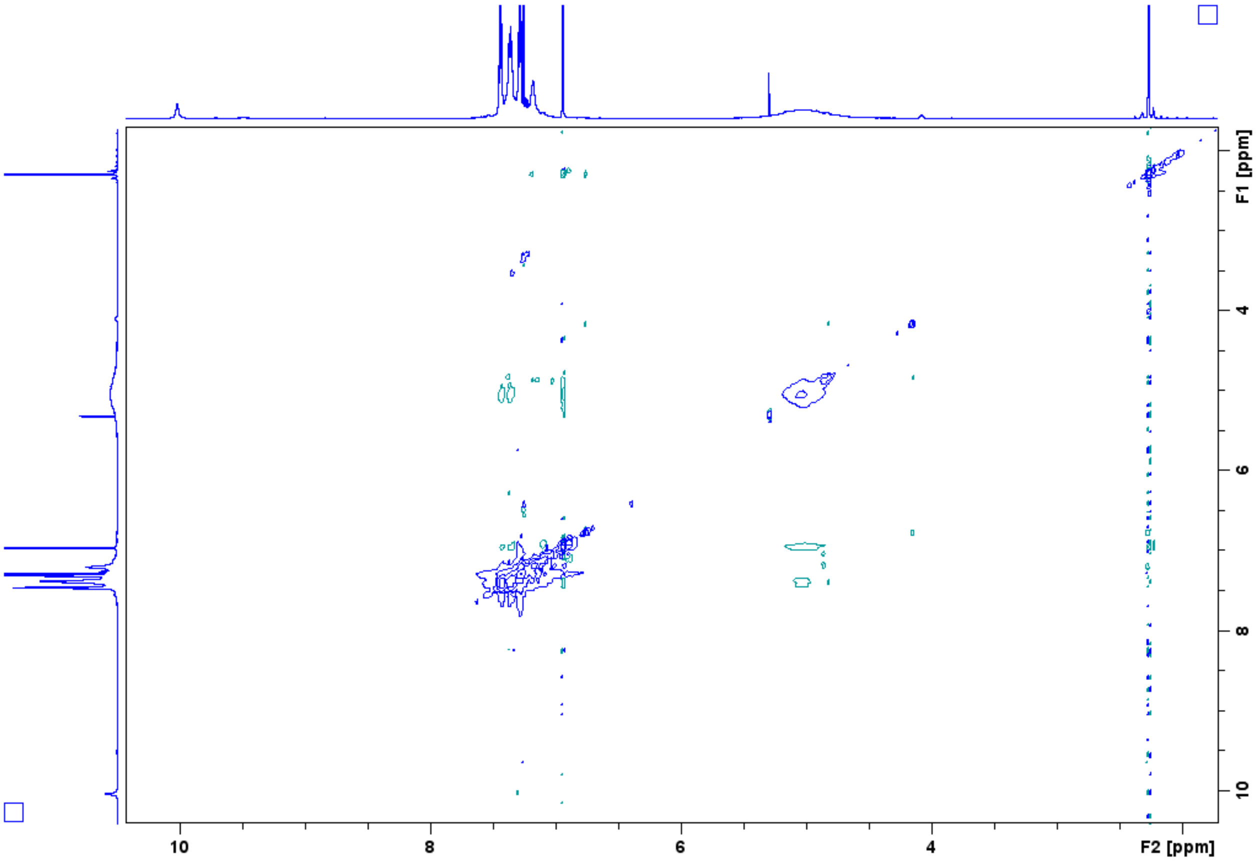

Figure S39. ^{1}H-^{1}H NOESY spectrum of compound **4ba**.

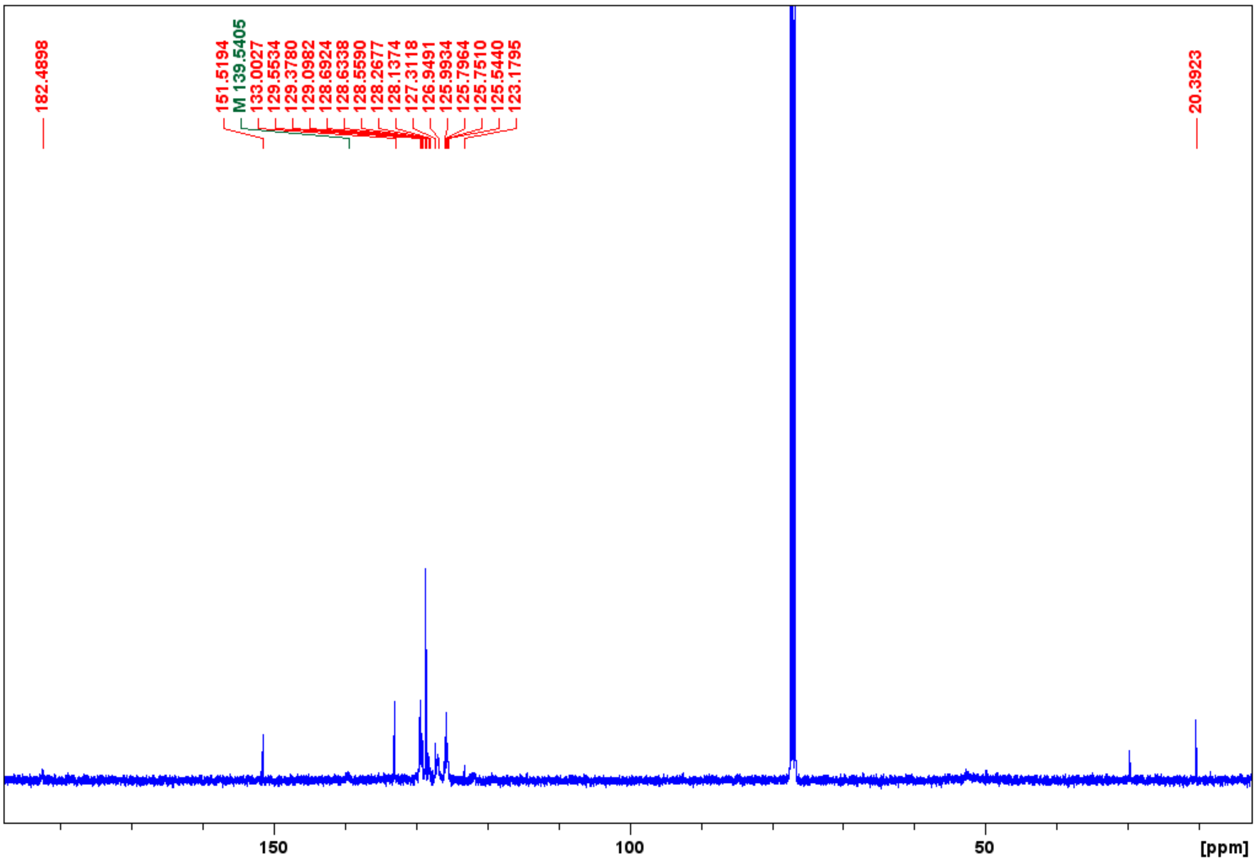

Figure S40. ^{13}C NMR spectrum of compound **4ba**.

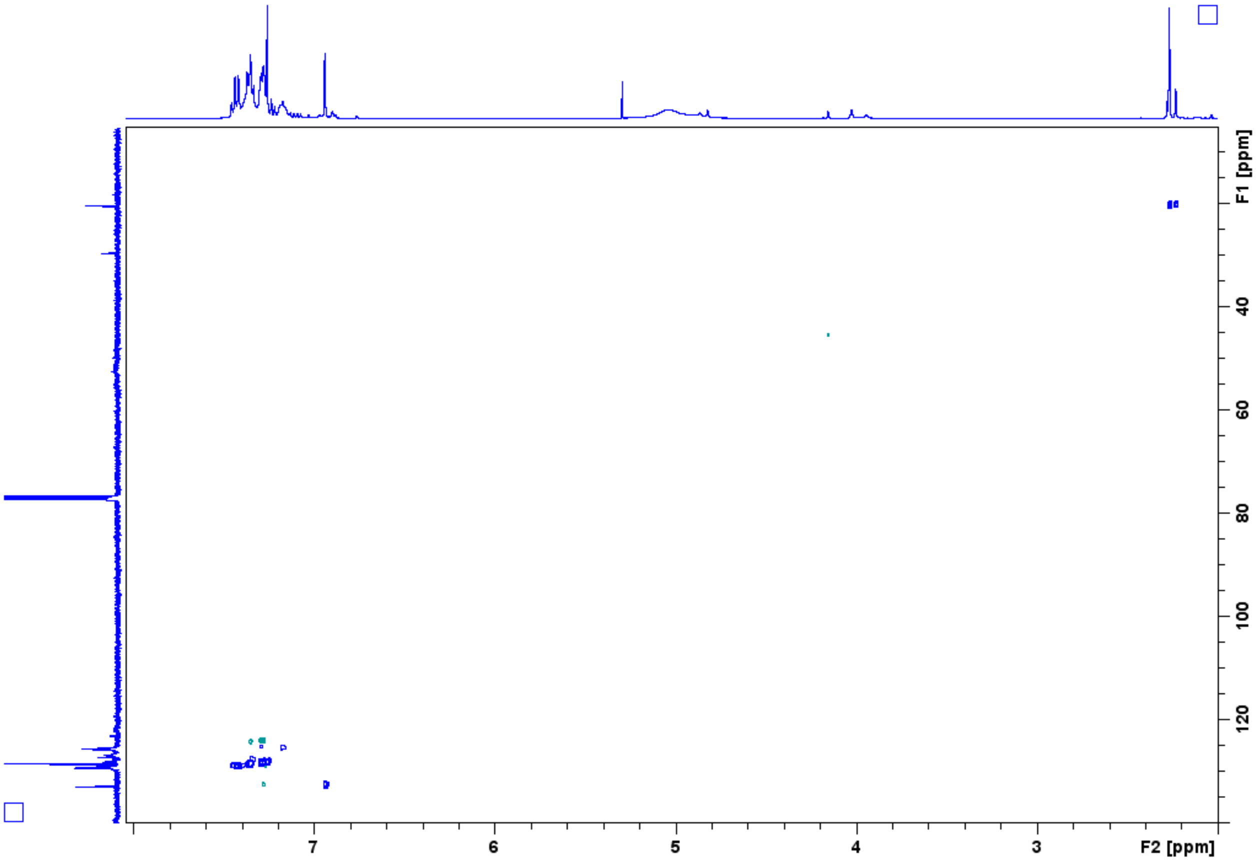

Figure S41. ^{1}H-^{13}C HSQC spectrum of compound **4ba**.

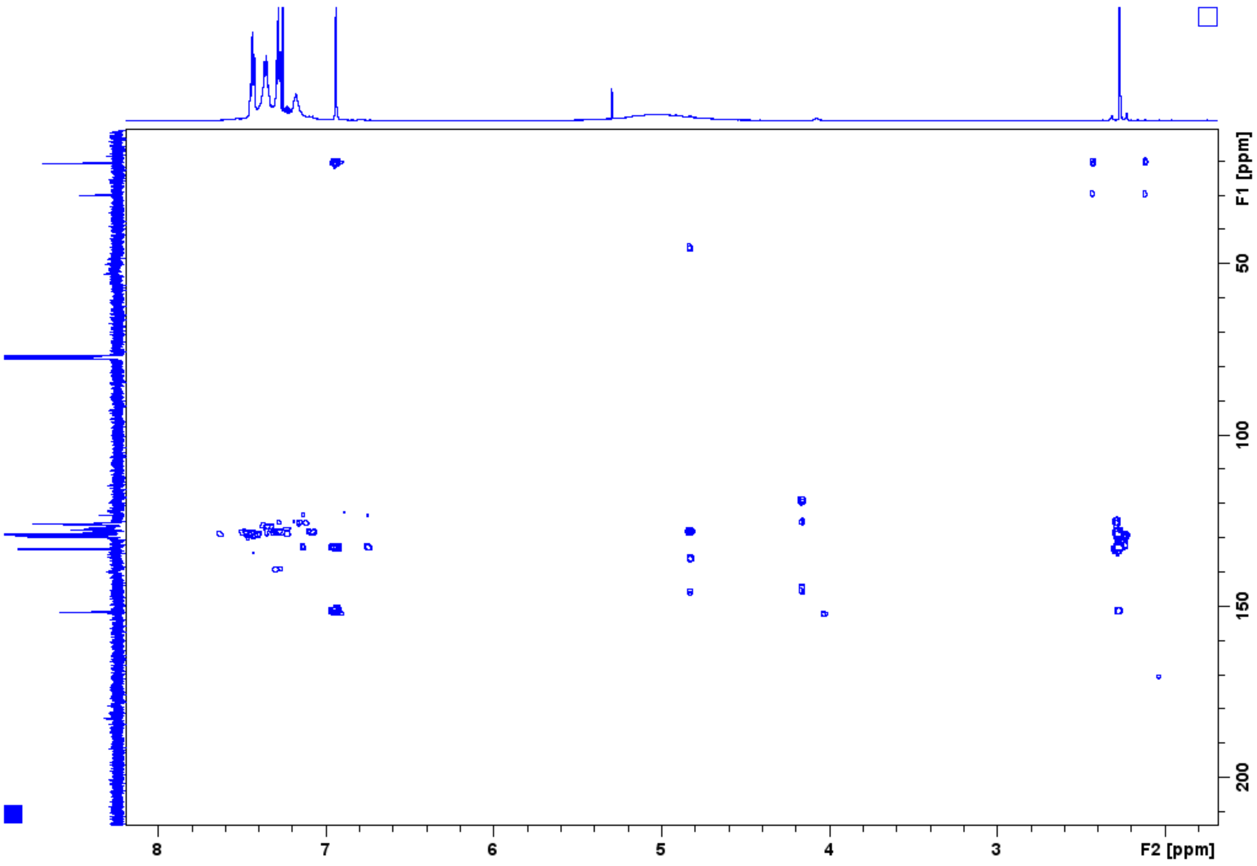

Figure S42. ^{1}H-^{13}C HMBC spectrum of compound **4ba**.

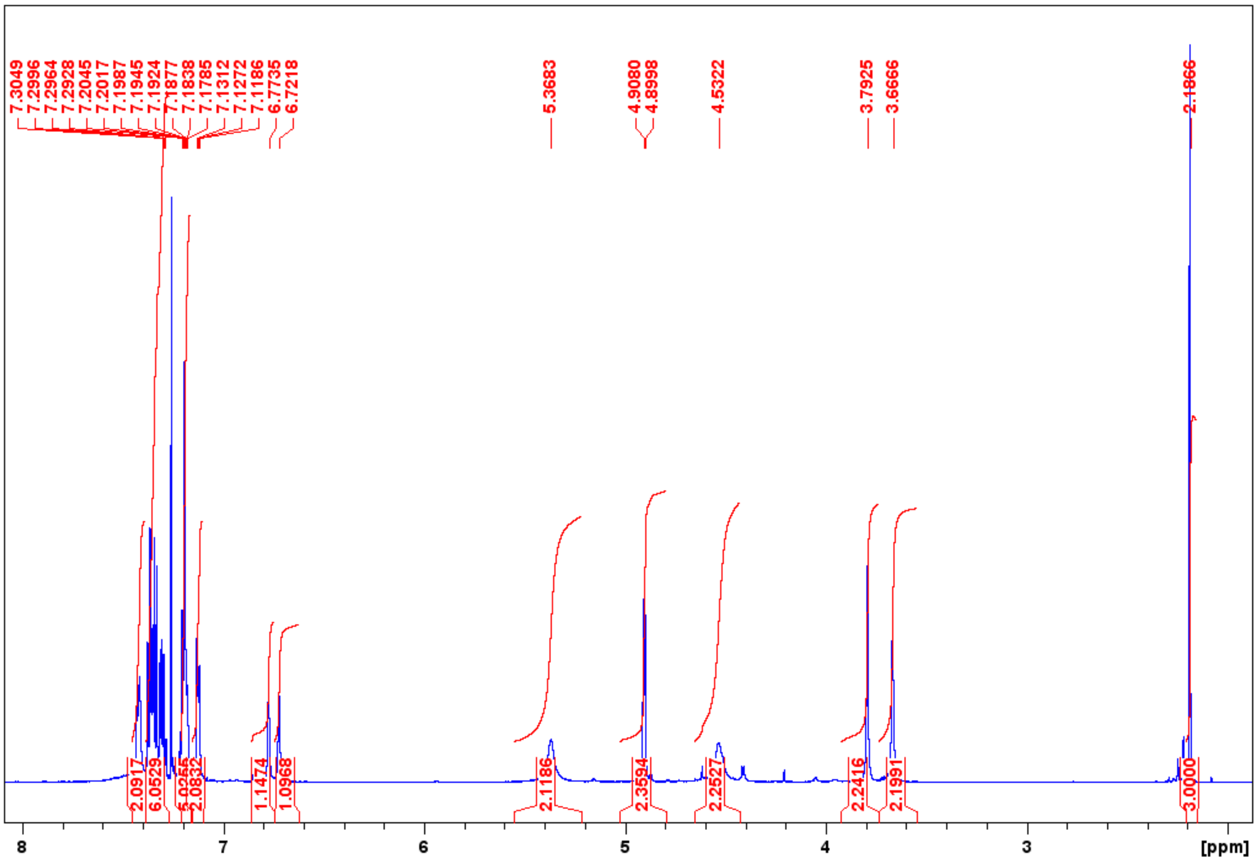

Figure S43. ^{1}H NMR spectrum of compound **3bb**.

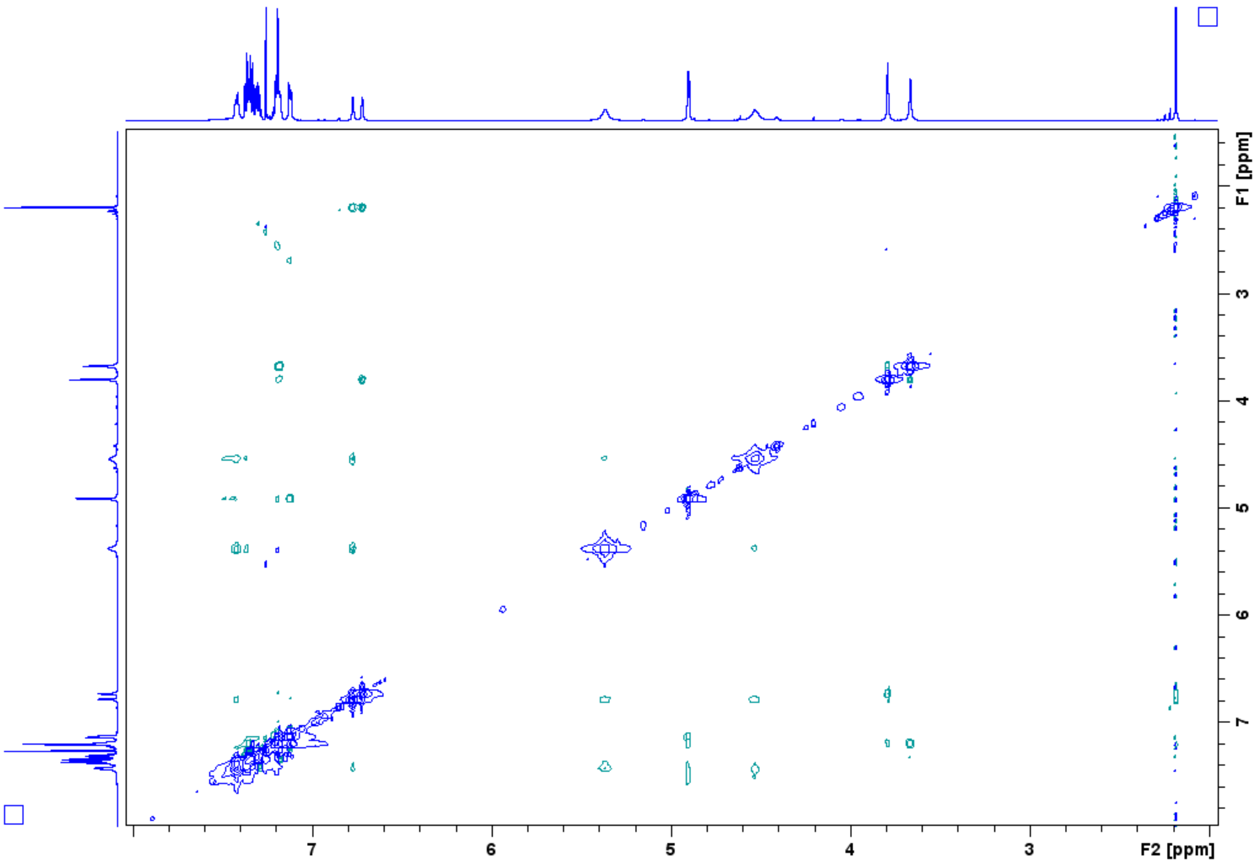

Figure S44. ^{1}H-^{1}H NOESY spectrum of compound **3bb**.

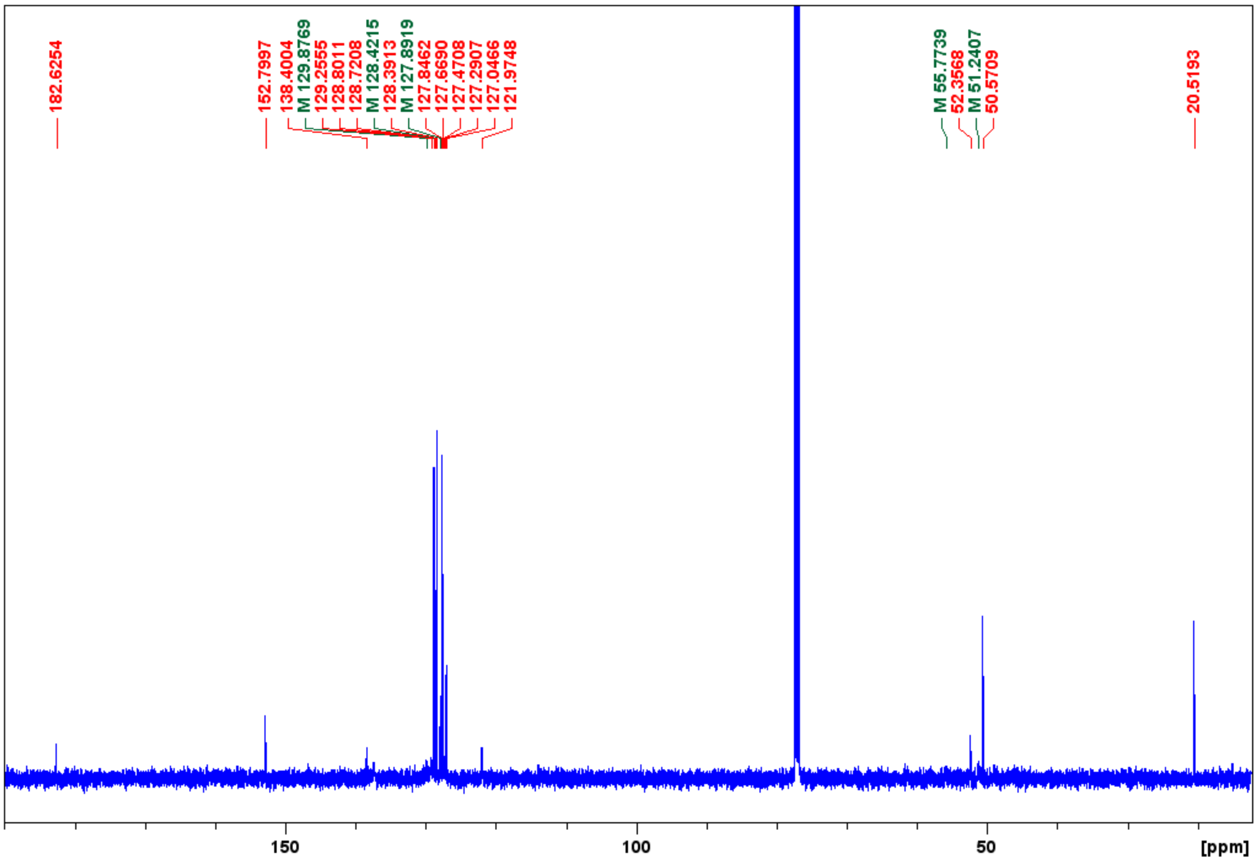

Figure S45. ^{13}C NMR spectrum of compound **3bb**.

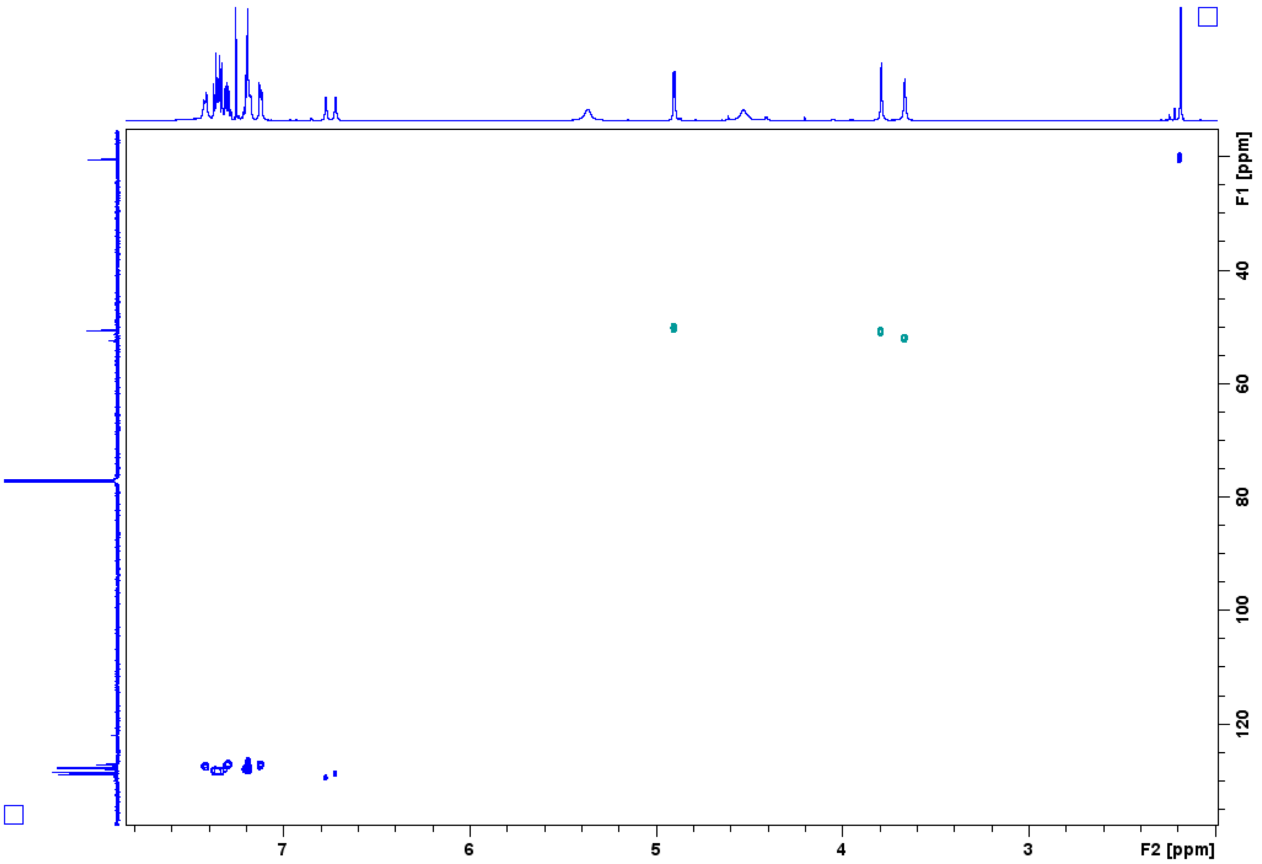

Figure S46. ^{1}H-^{13}C HSQC spectrum of compound **3bb**.

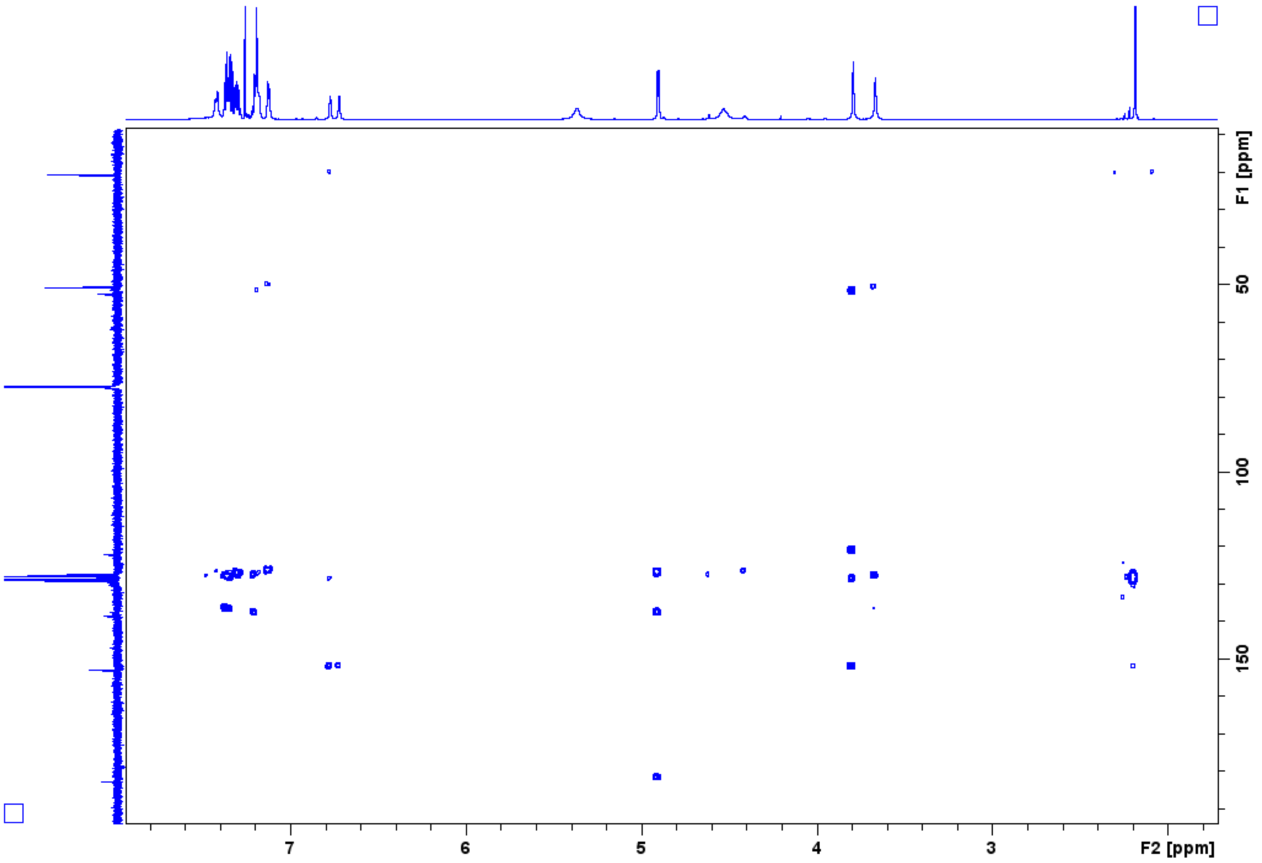

Figure S47. ^{1}H-^{13}C HMBC spectrum of compound **3bb**.

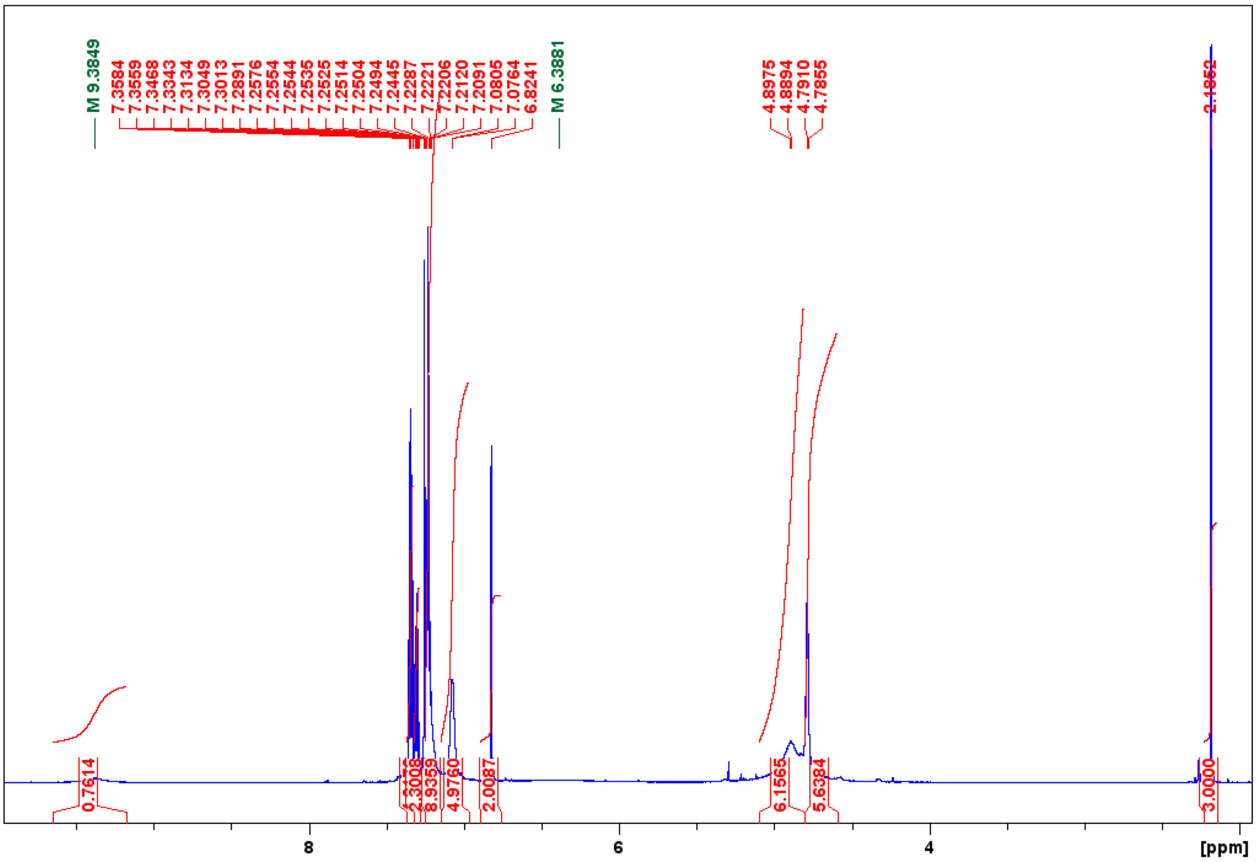

Figure S48. ^{1}H NMR spectrum of compound **4bb**.

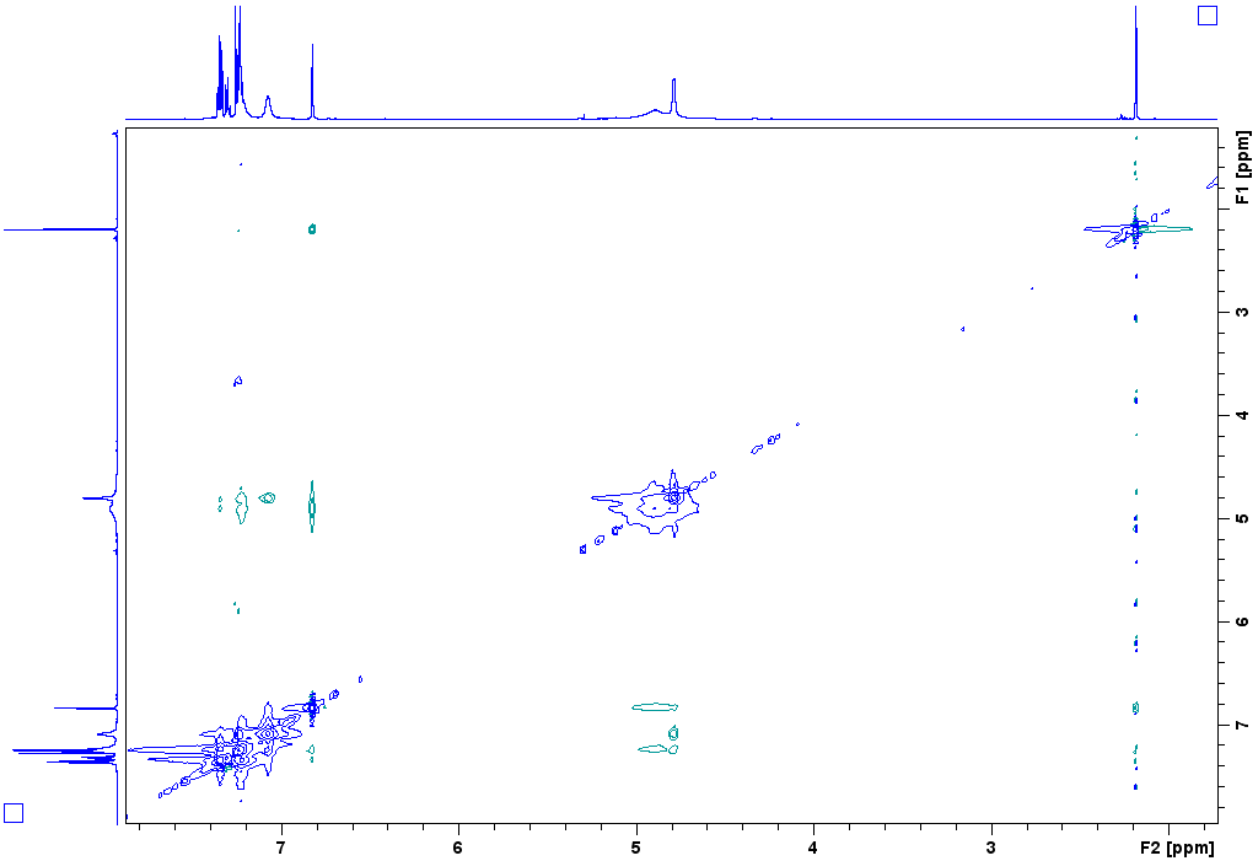

Figure S49. ^{1}H-^{1}H NOESY spectrum of compound **4bb**.

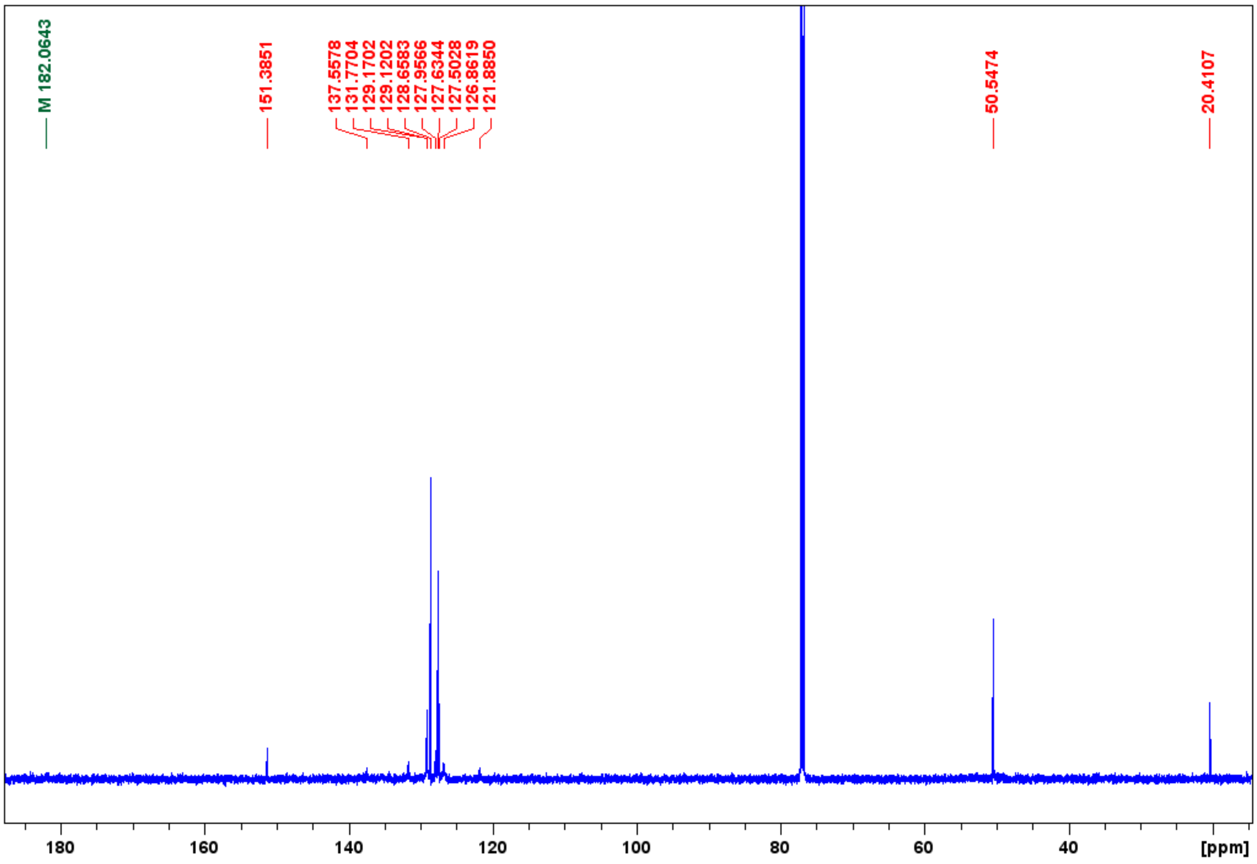

Figure S50. ^{13}C NMR spectrum of compound **4bb**.

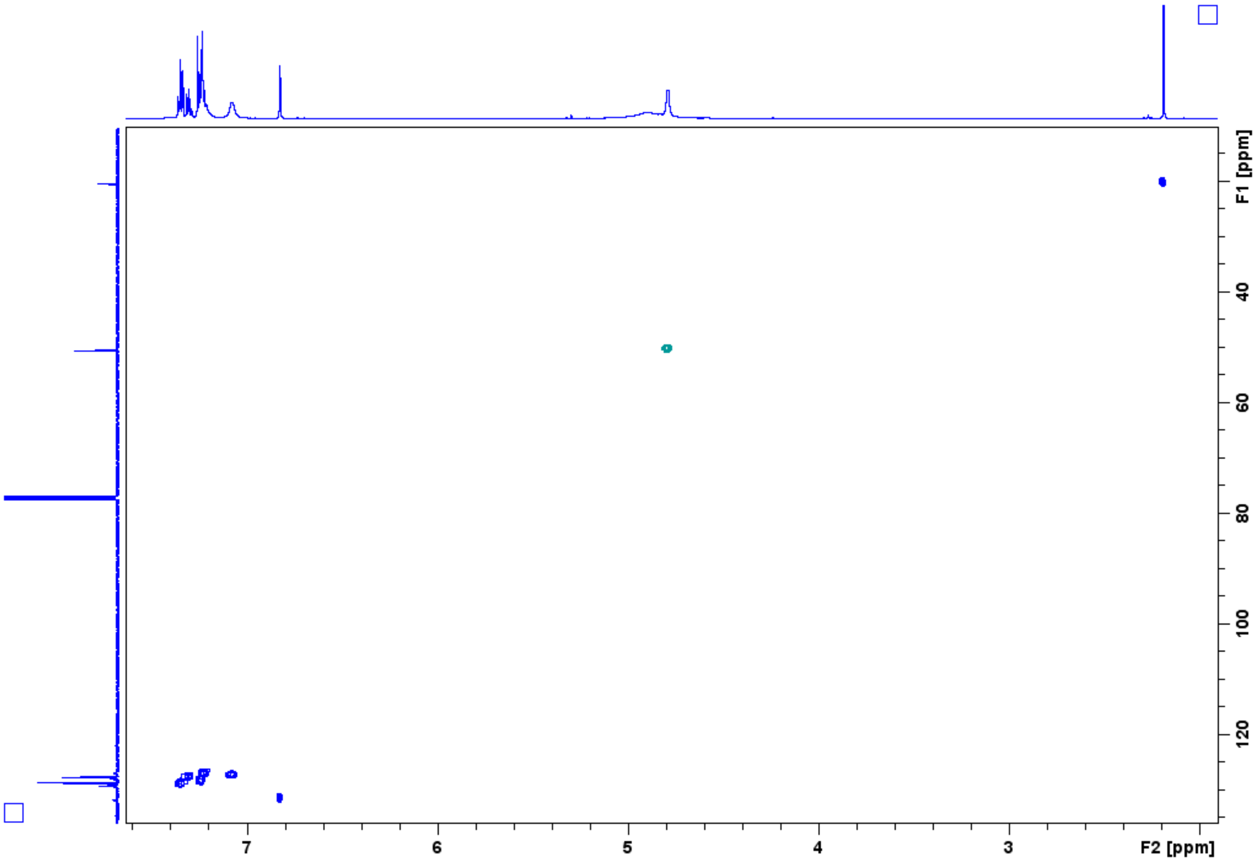

Figure S51. ^{1}H-^{13}C HSQC spectrum of compound **4bb**.

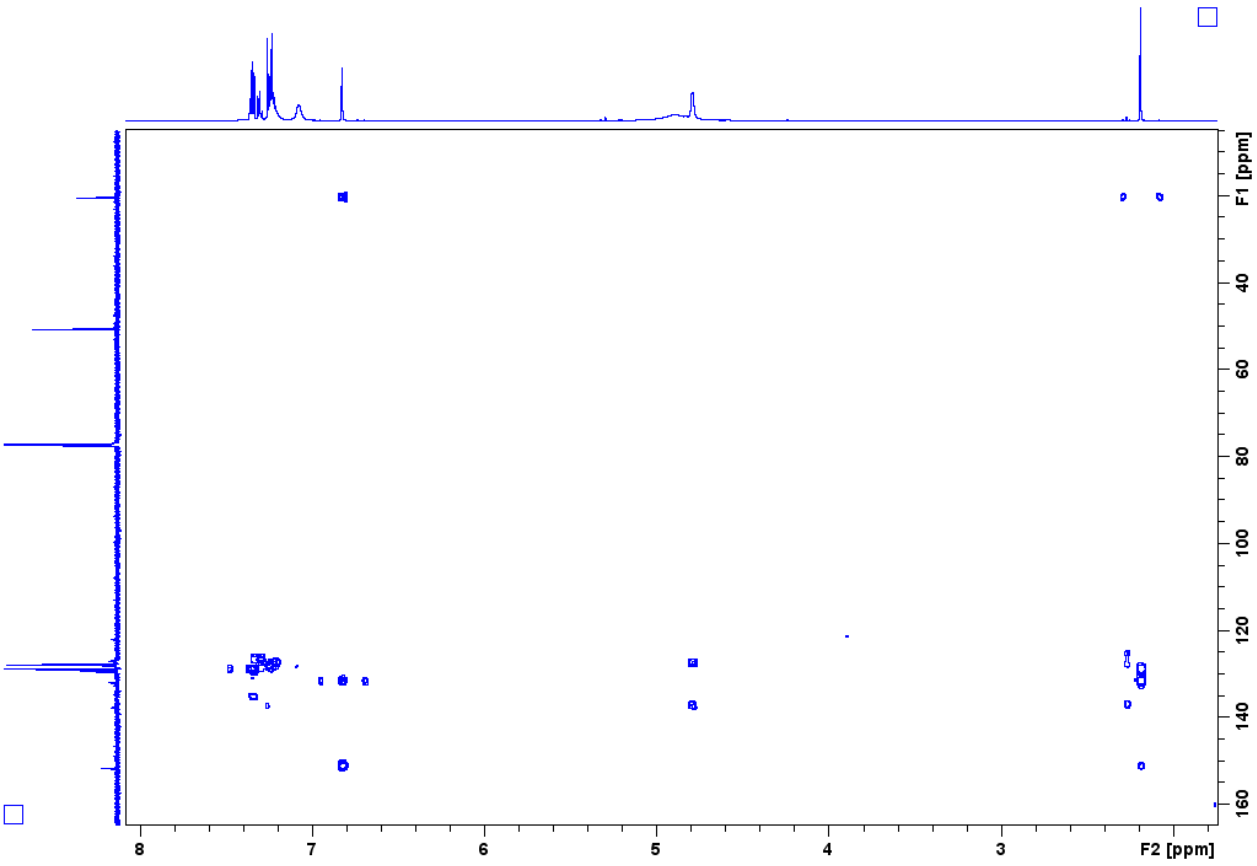

Figure S52. ^{1}H-^{13}C HMBC spectrum of compound **4bb**.

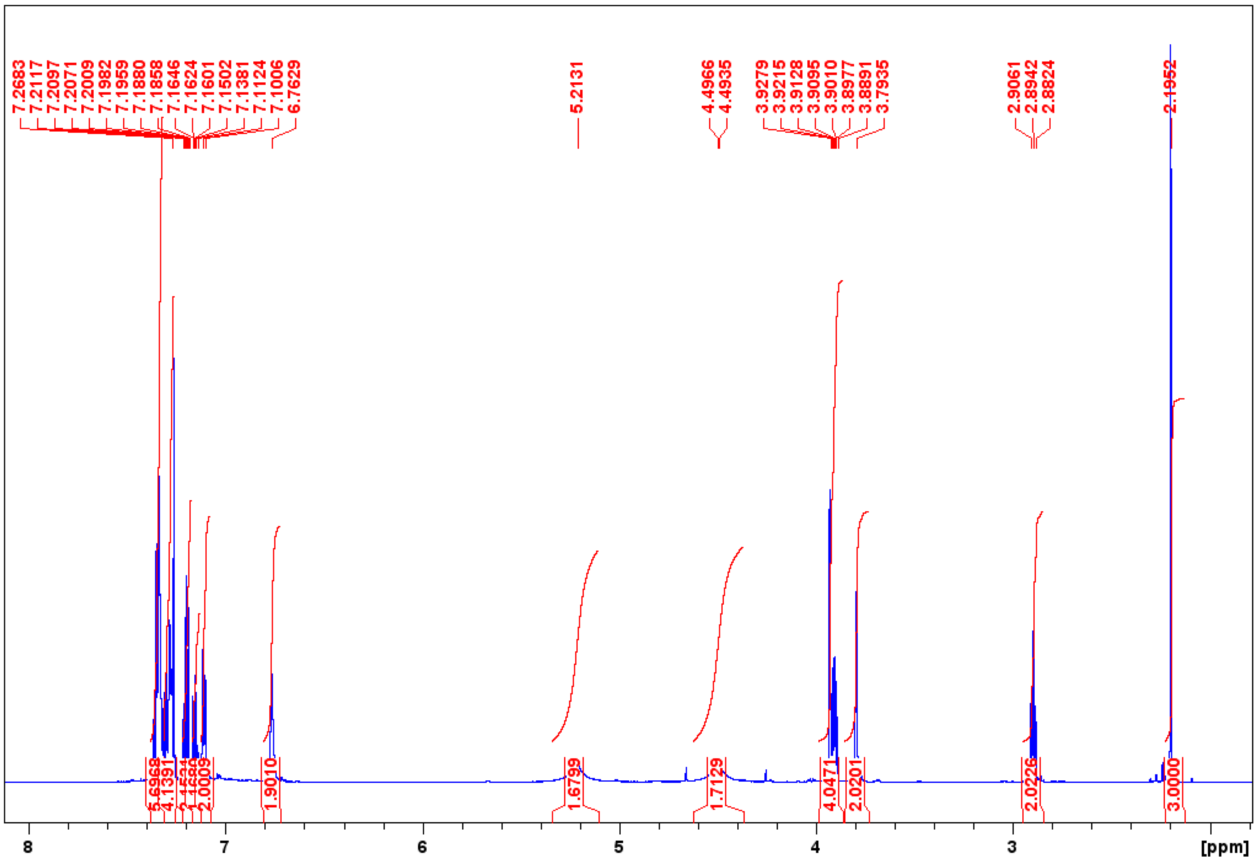

Figure S53. ^{1}H NMR spectrum of compound **3bc**.

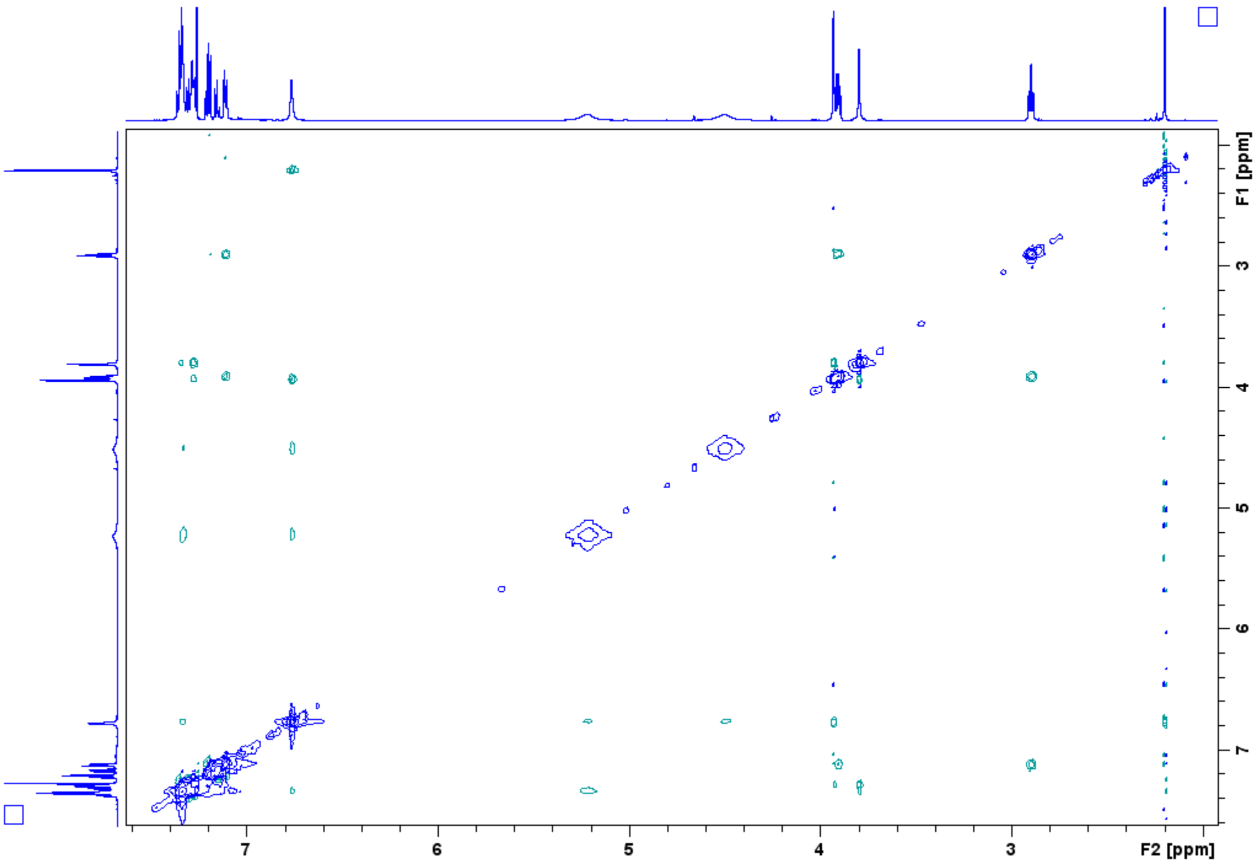

Figure S54. ^{1}H-^{1}H NOESY spectrum of compound **3bc**.

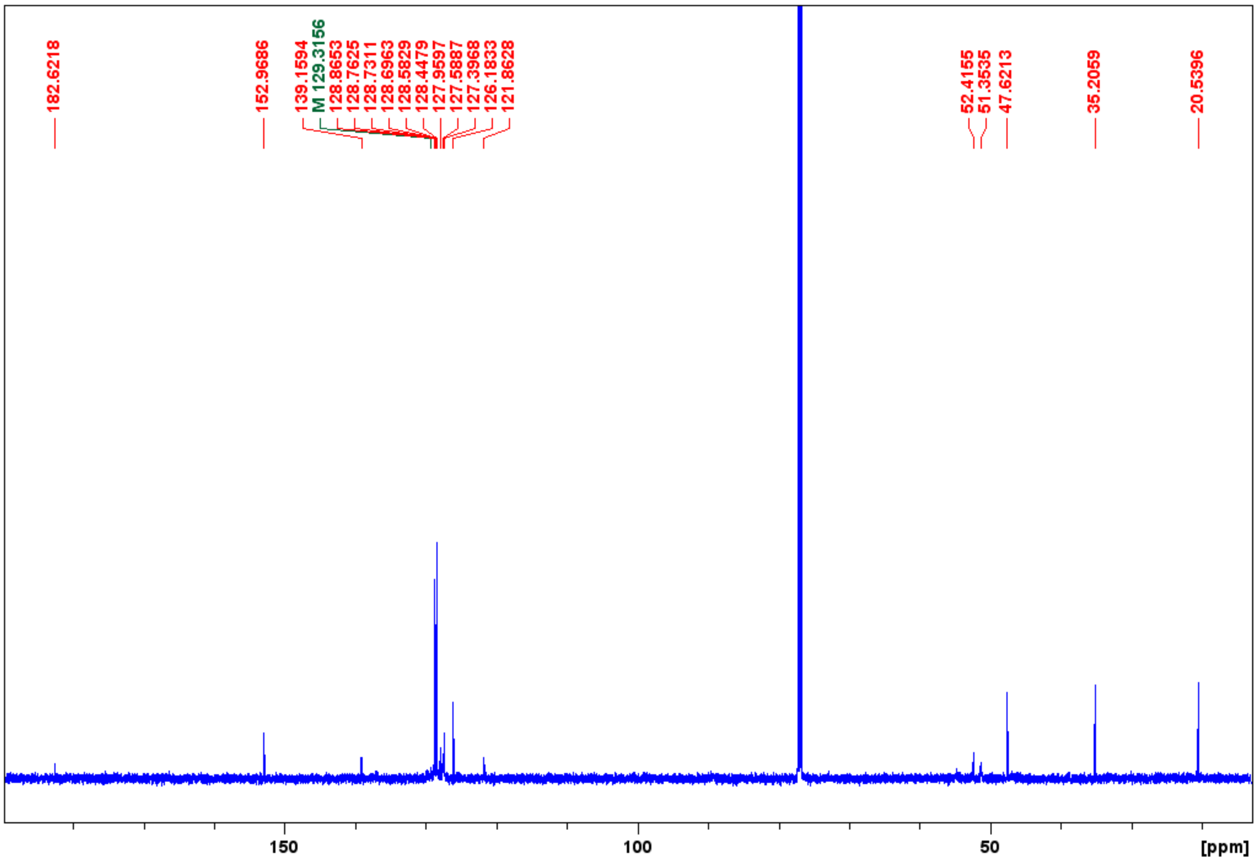

Figure S55. ^{13}C NMR spectrum of compound **3bc**.

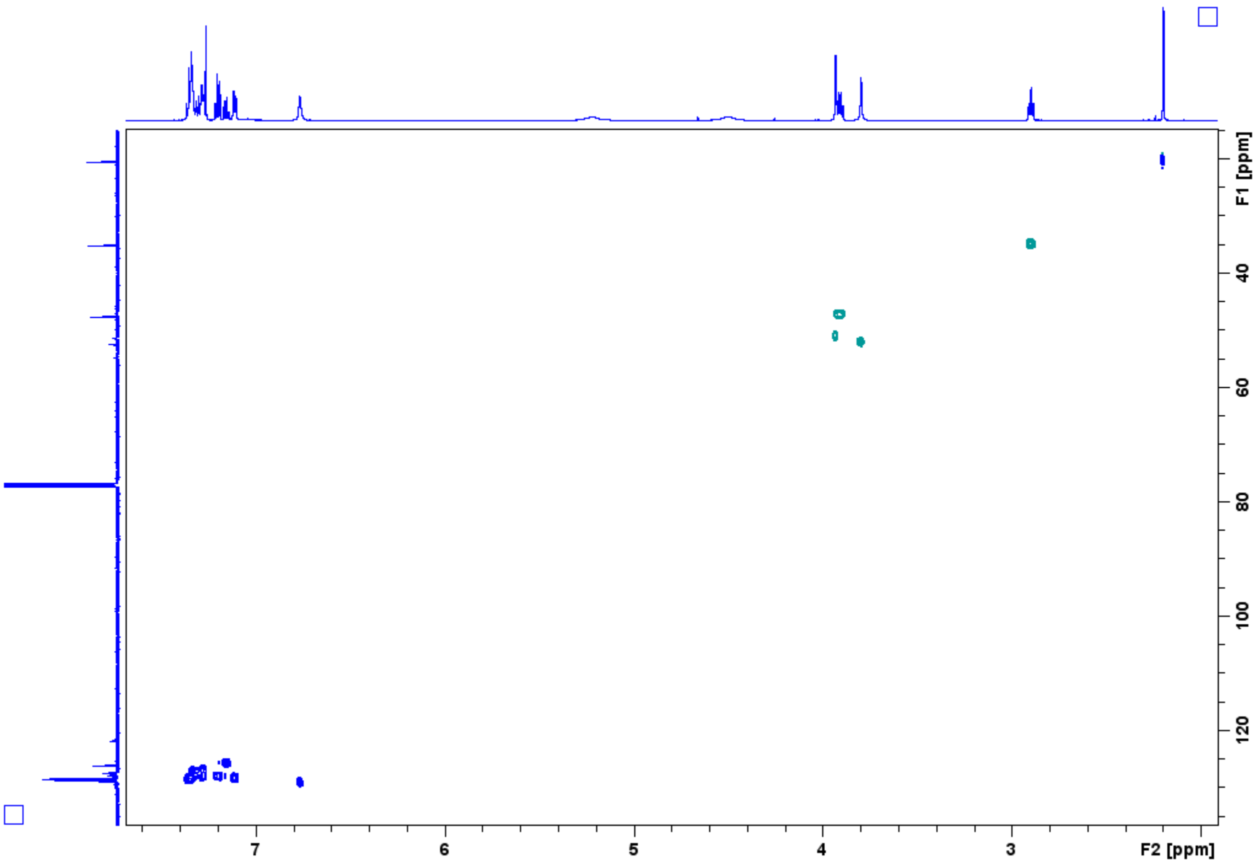

Figure S56. ^{1}H-^{13}C HSQC spectrum of compound **3bc**.

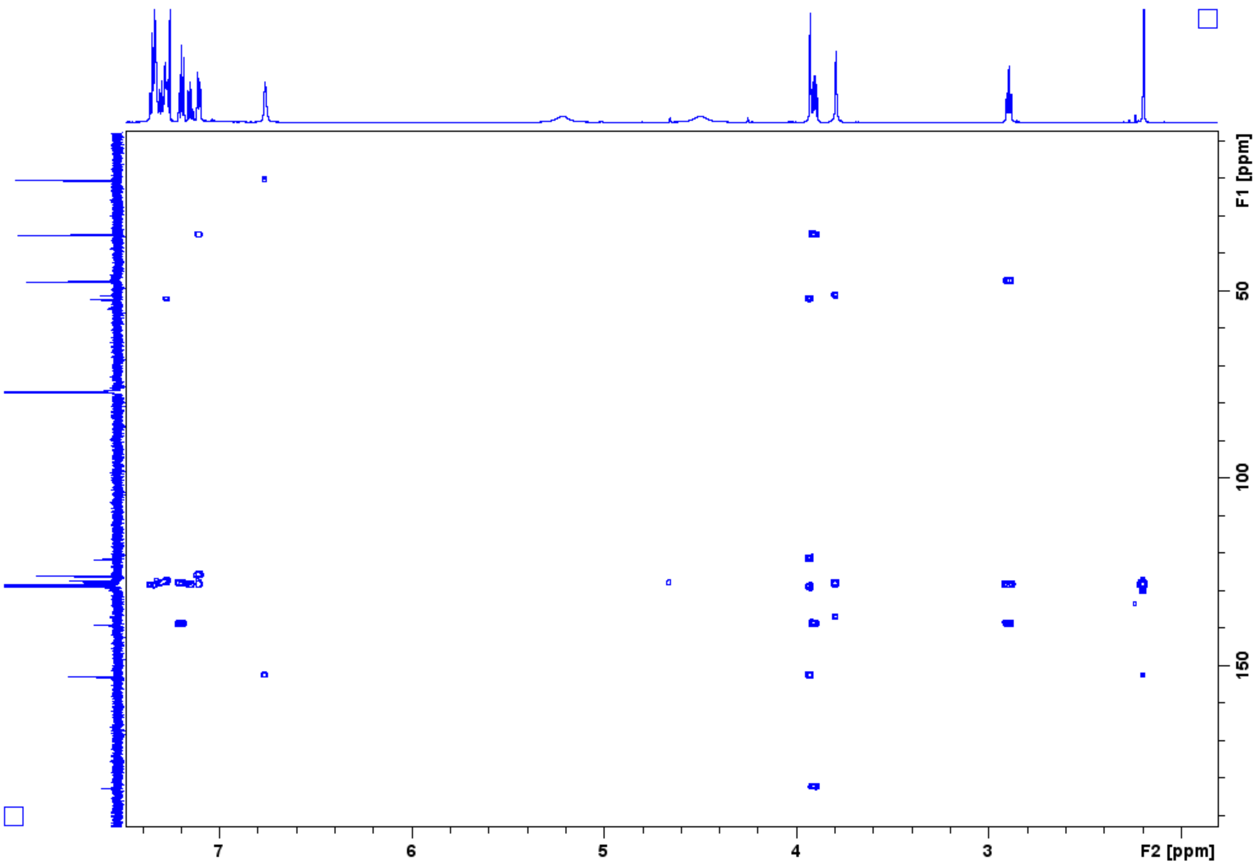

Figure S57. ^{1}H-^{13}C HMBC spectrum of compound **3bc**.

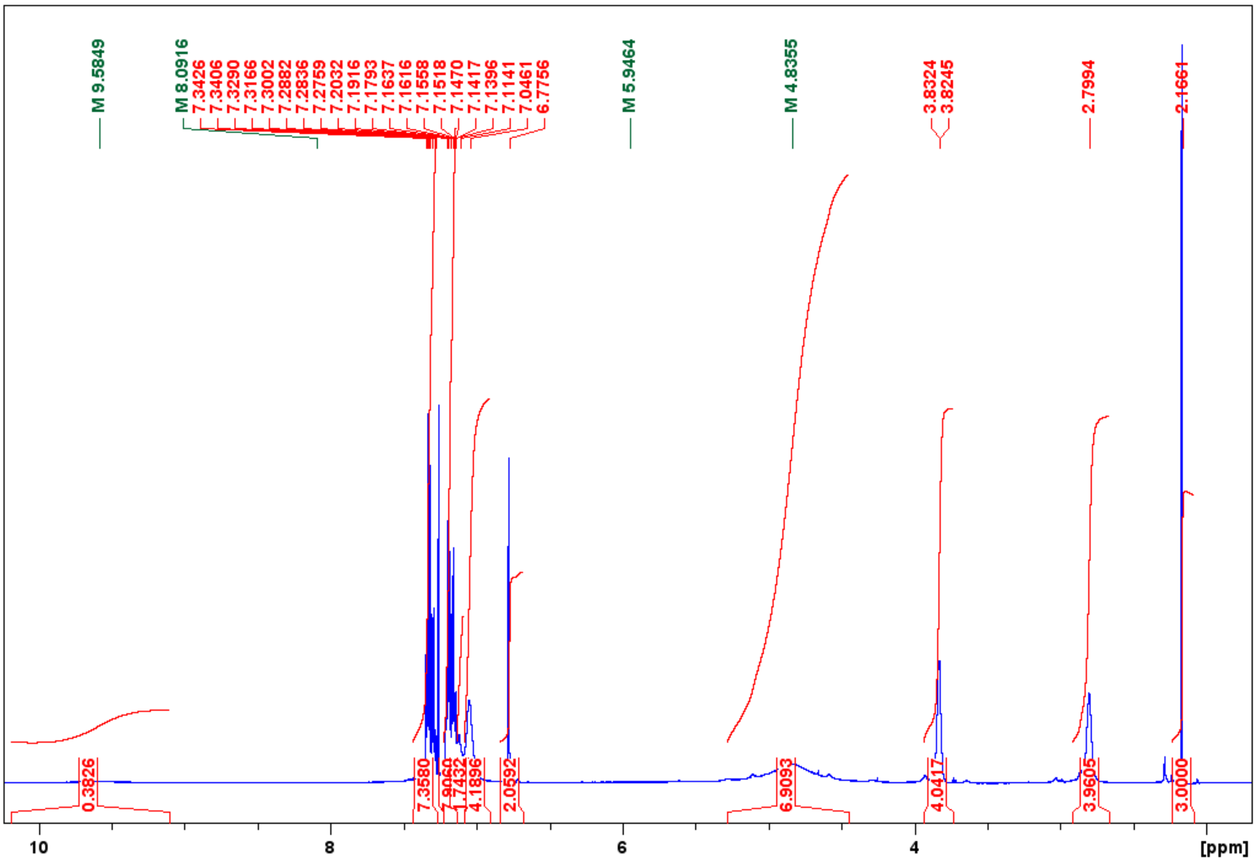

Figure S58. ^{1}H NMR spectrum of compound **4bc**.

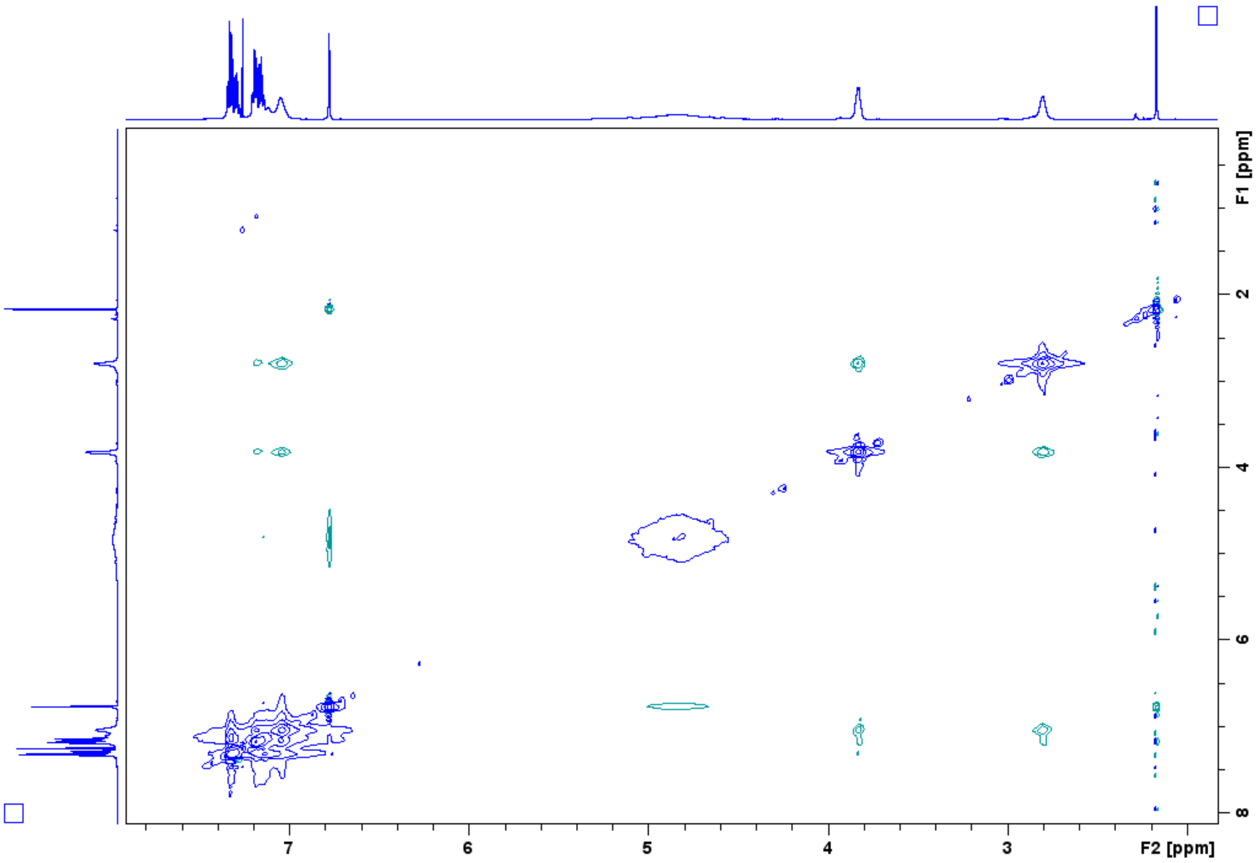

Figure S59. ^{1}H-^{1}H NOESY spectrum of compound **4bc**.

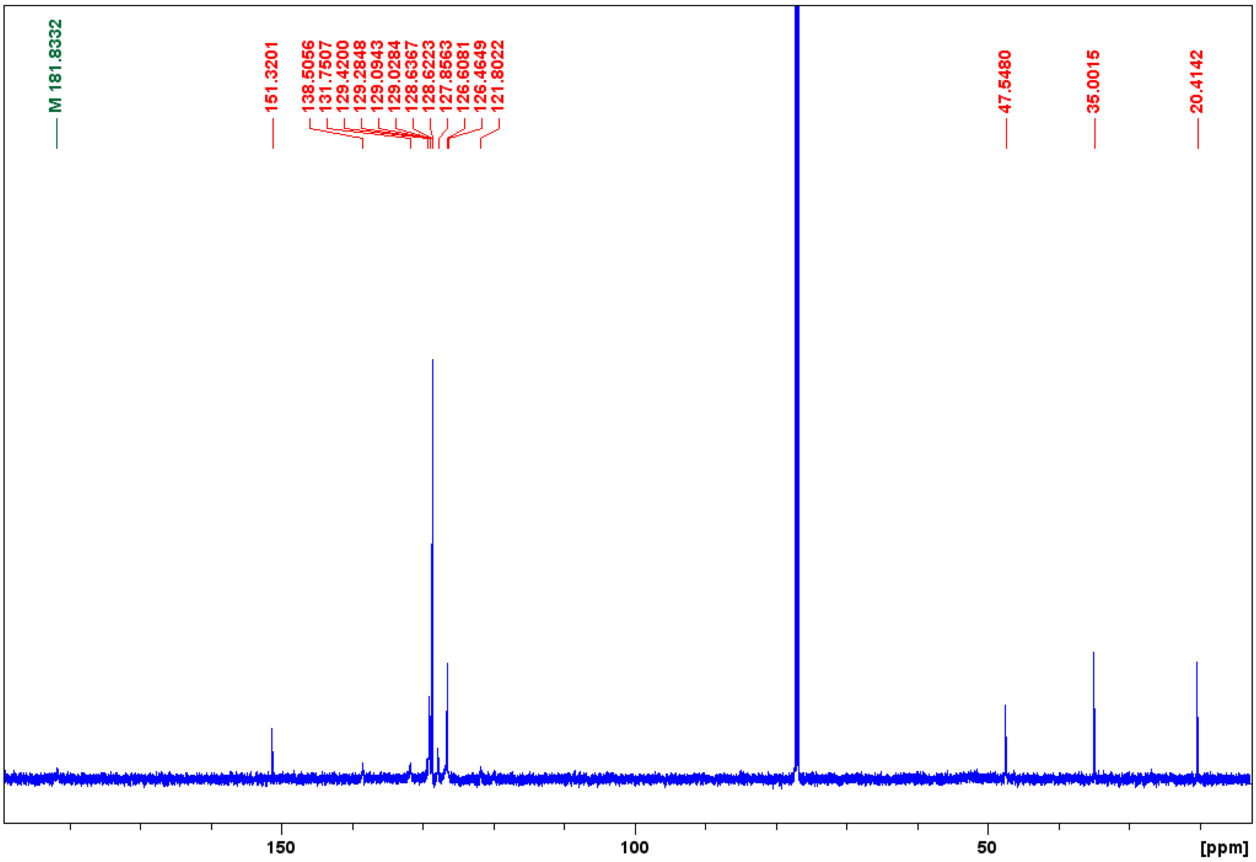

Figure S60. ^{13}C NMR spectrum of compound **4bc**.

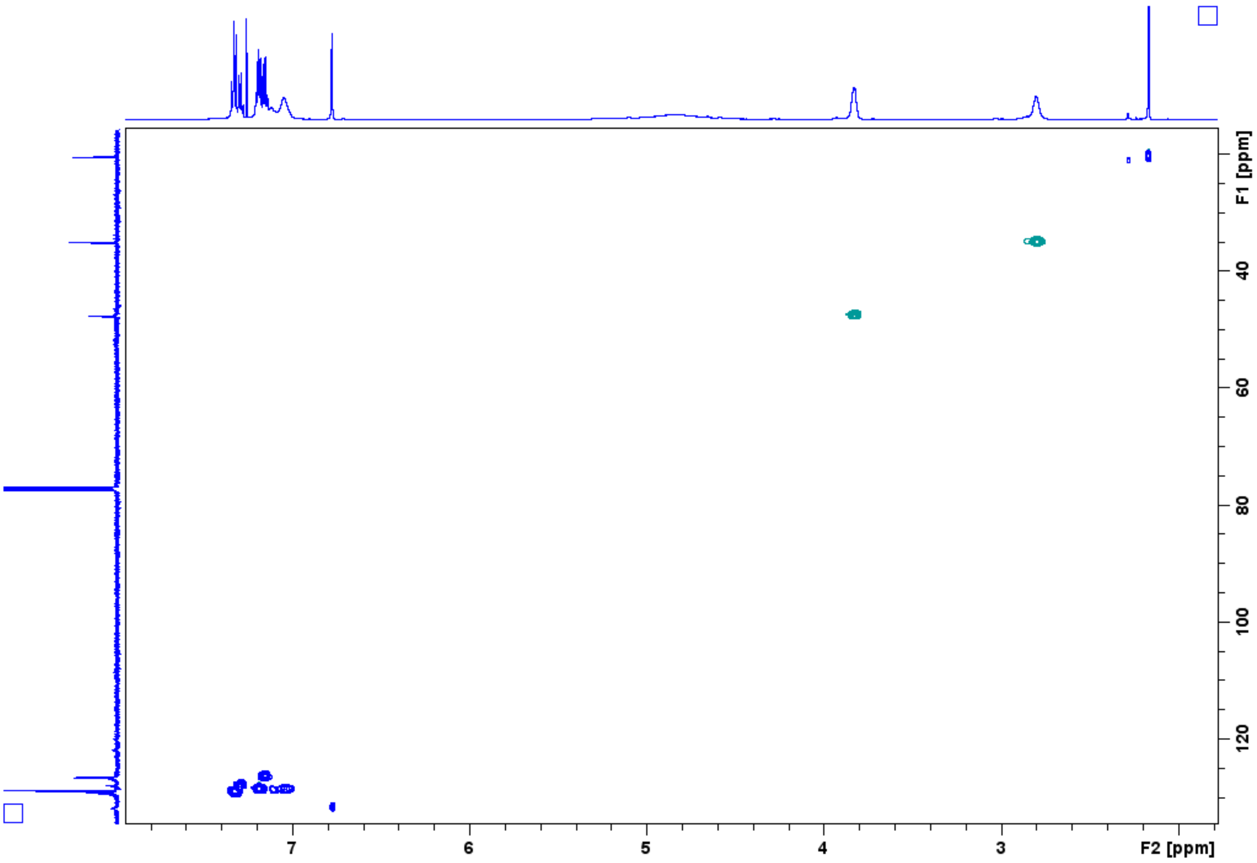

Figure S61. ^{1}H-^{13}C HSQC spectrum of compound **4bc**.

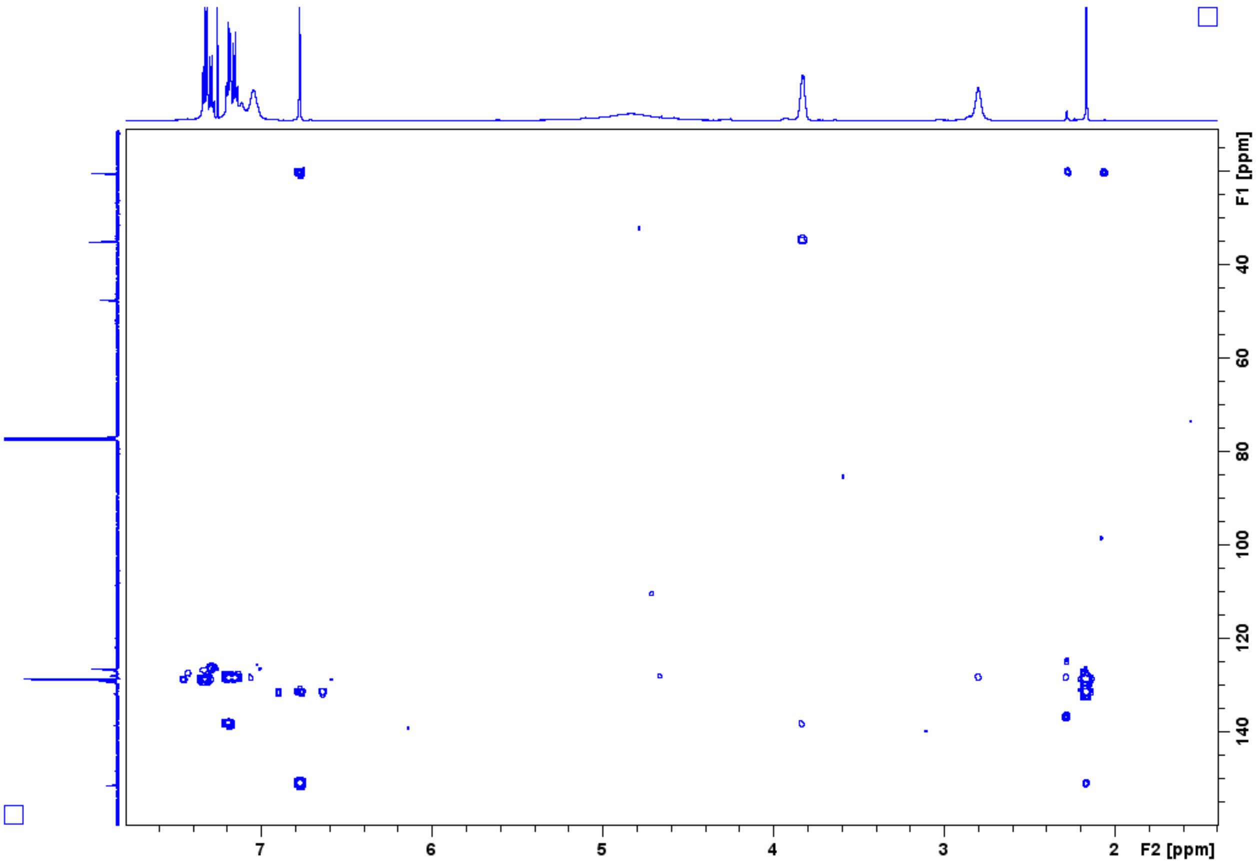

Figure S62. ^{1}H-^{13}C HMBC spectrum of compound **4bc**.

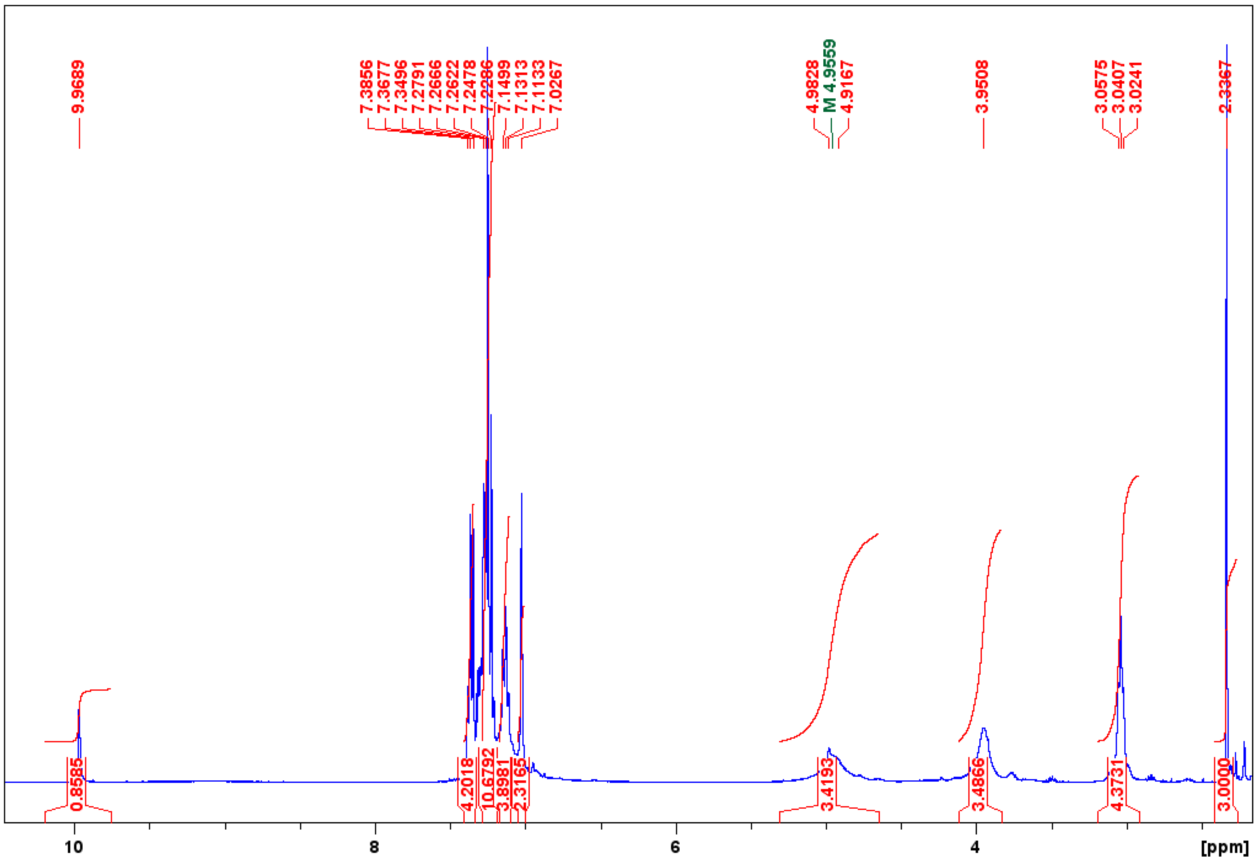

Figure S63. ^{1}H NMR spectrum of compound **4ca**.

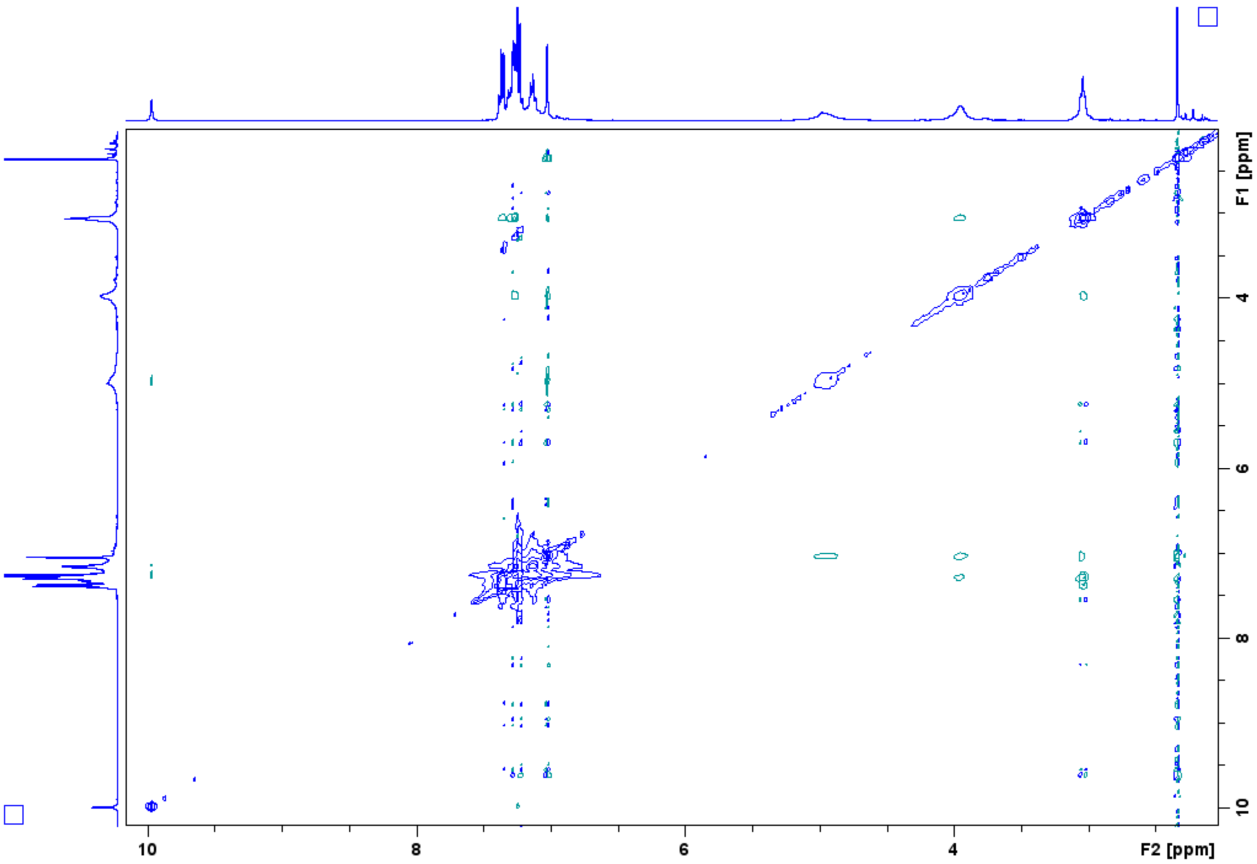

Figure S64. ^{1}H-^{1}H NOESY spectrum of compound **4ca**.

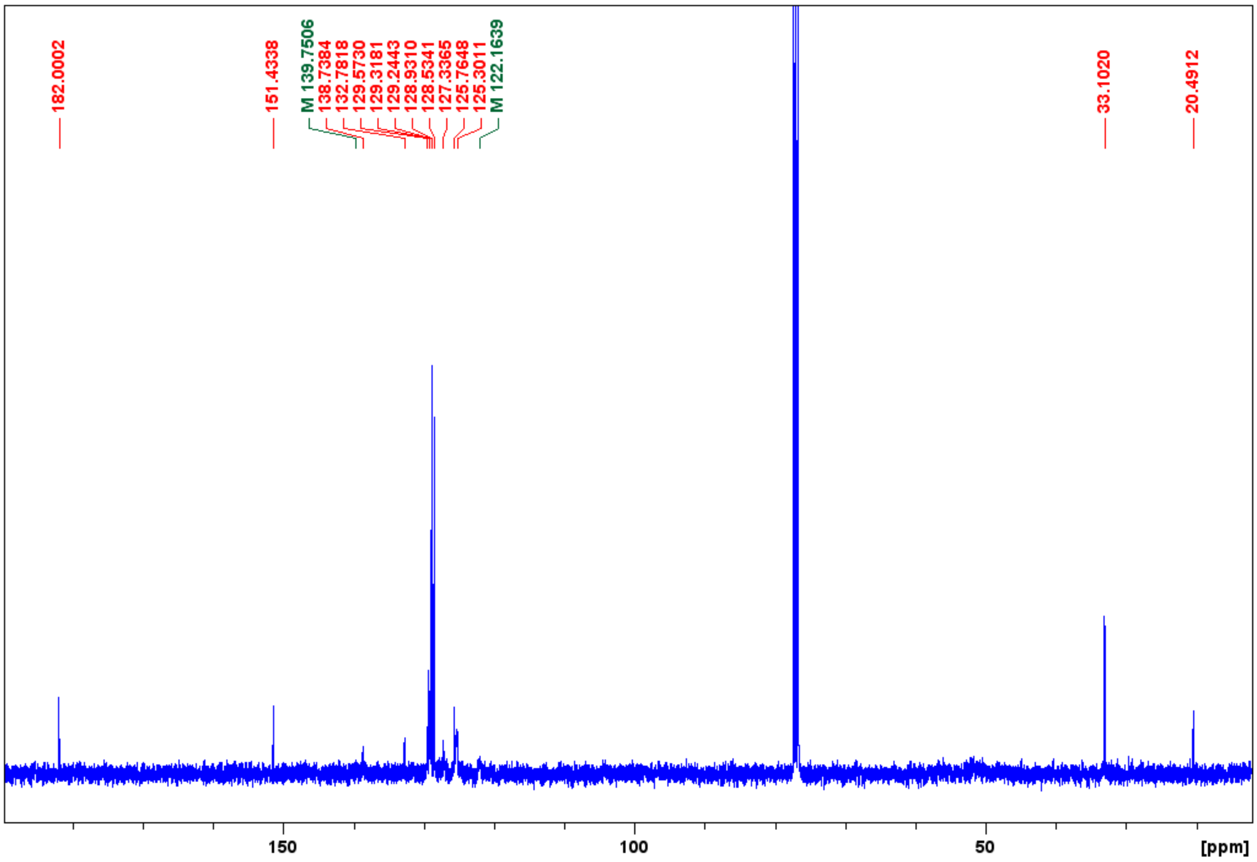

Figure S65. ^{13}C NMR spectrum of compound **4ca**.

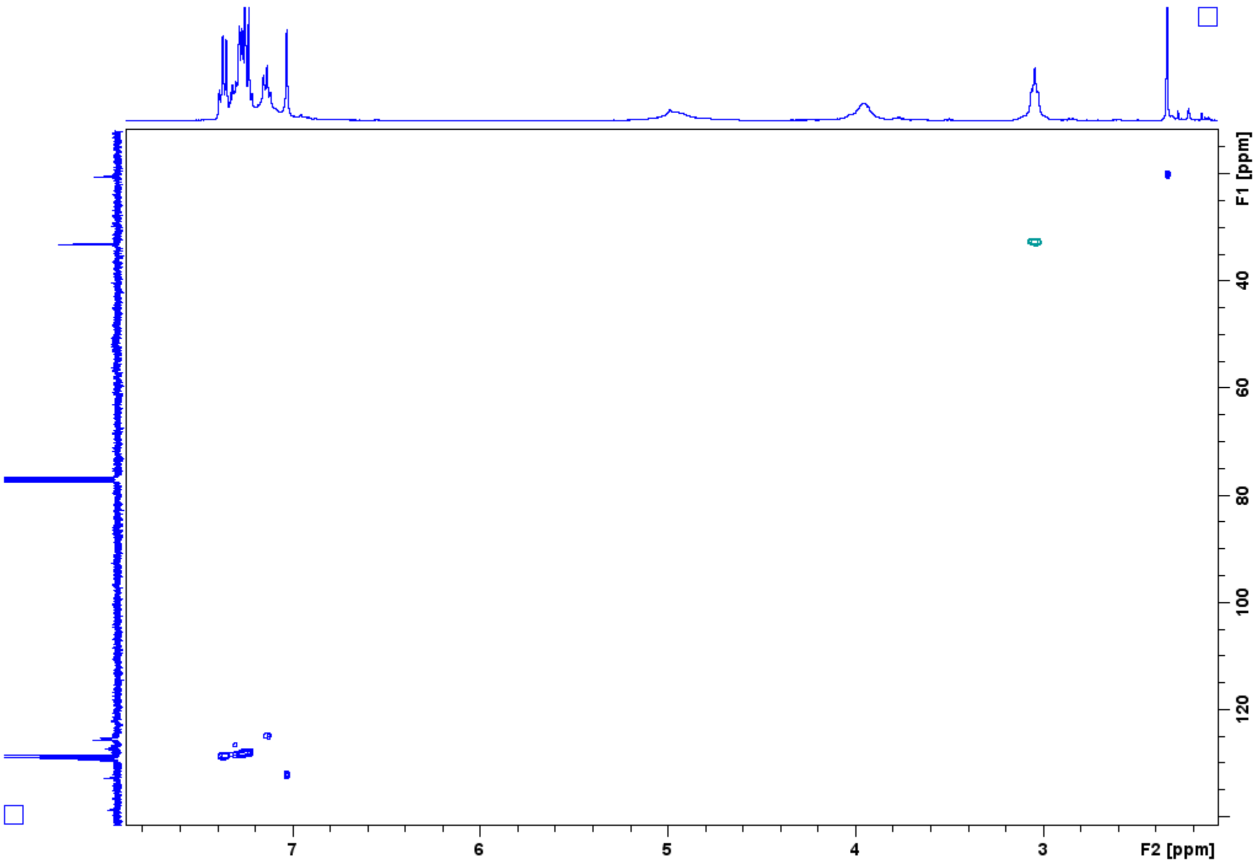

Figure S66. ^{1}H-^{13}C HSQC spectrum of compound **4ca**.

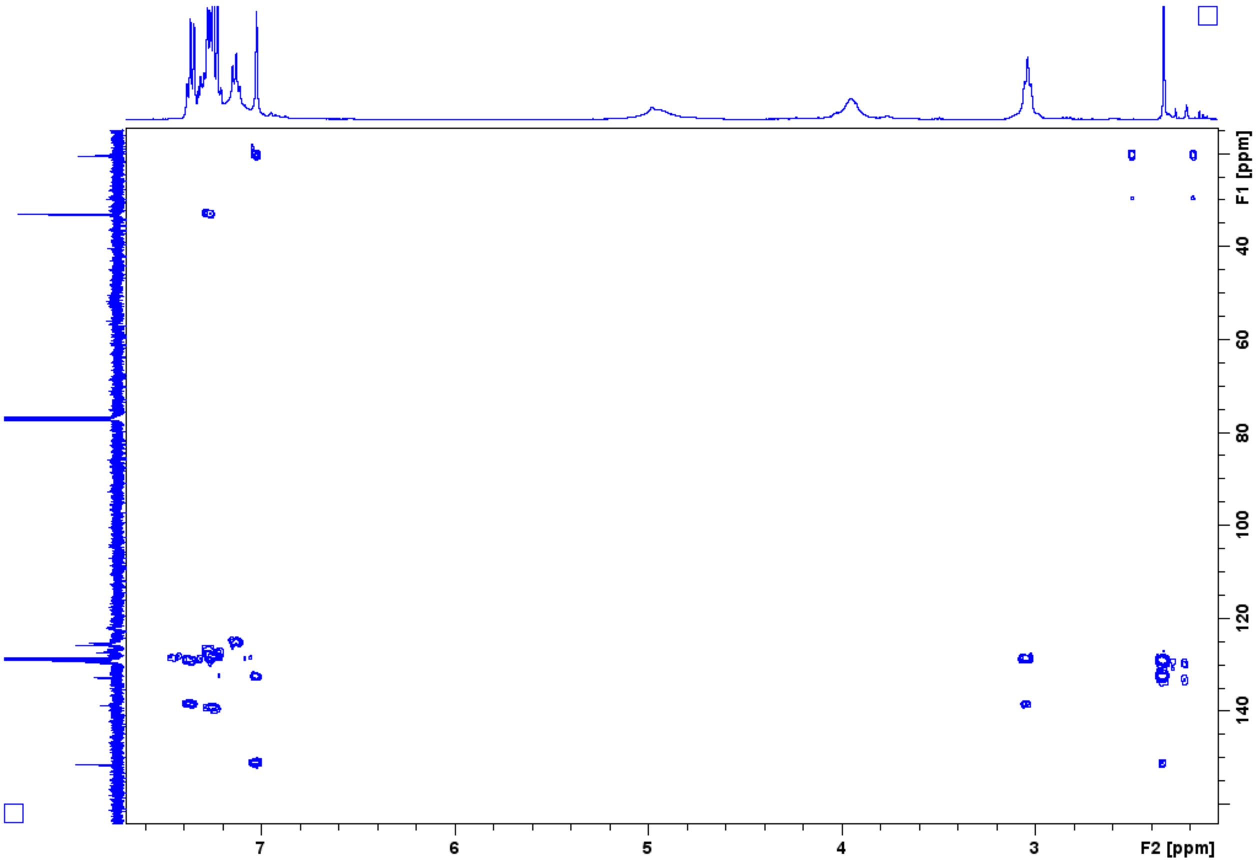

Figure S67. ^{1}H-^{13}C HMBC spectrum of compound **4ca**.

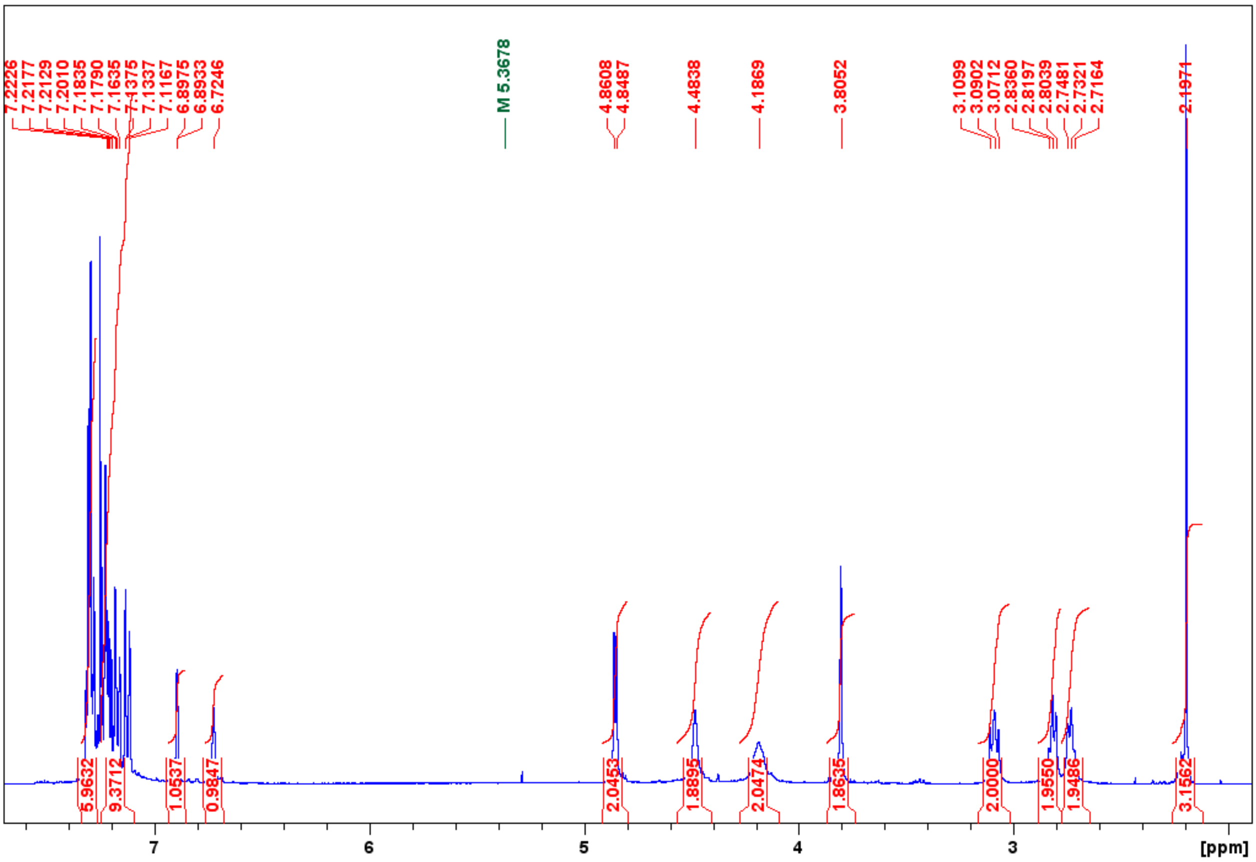

Figure S68. ^{1}H NMR spectrum of compound **3cb**.

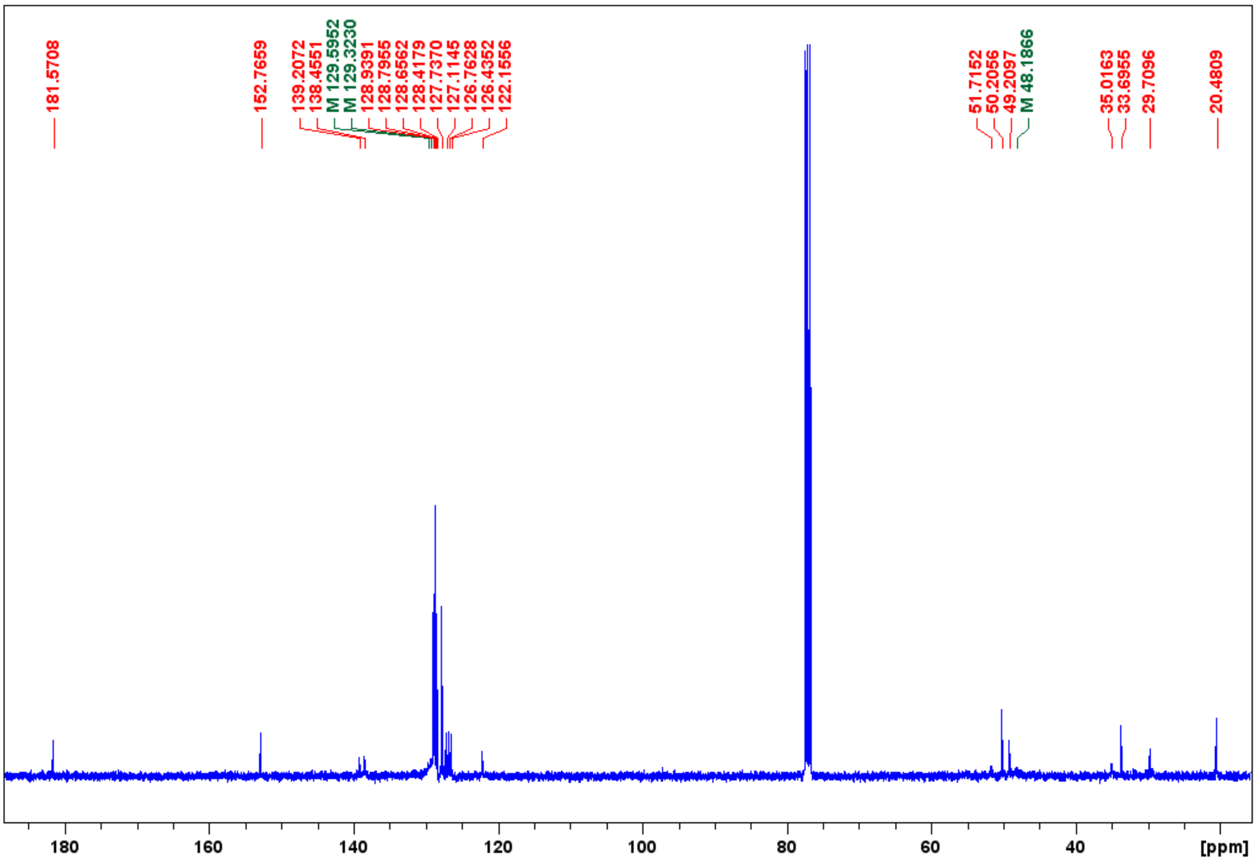

Figure S69. ^{13}C NMR spectrum of compound **3cb**.

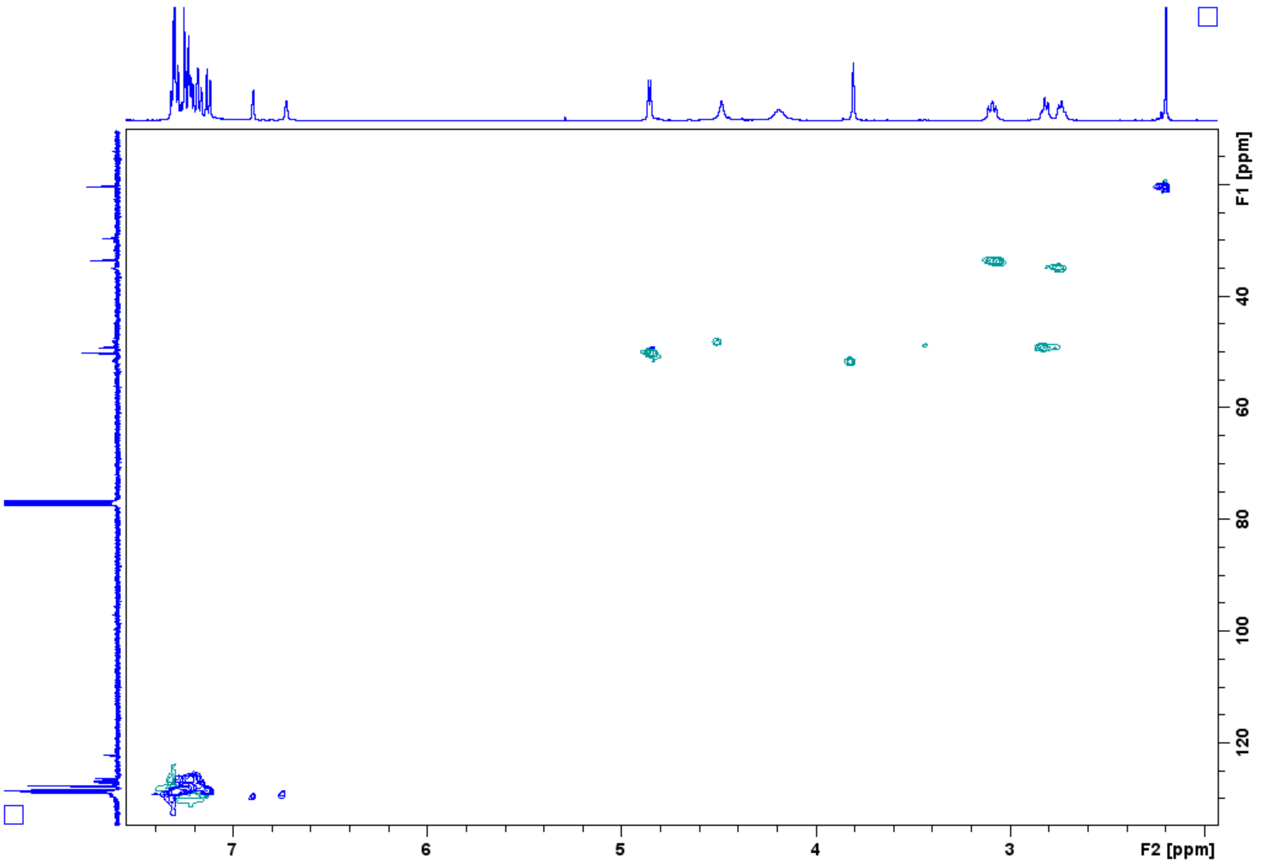

Figure S70. ^{1}H-^{13}C HSQC spectrum of compound **3cb**.

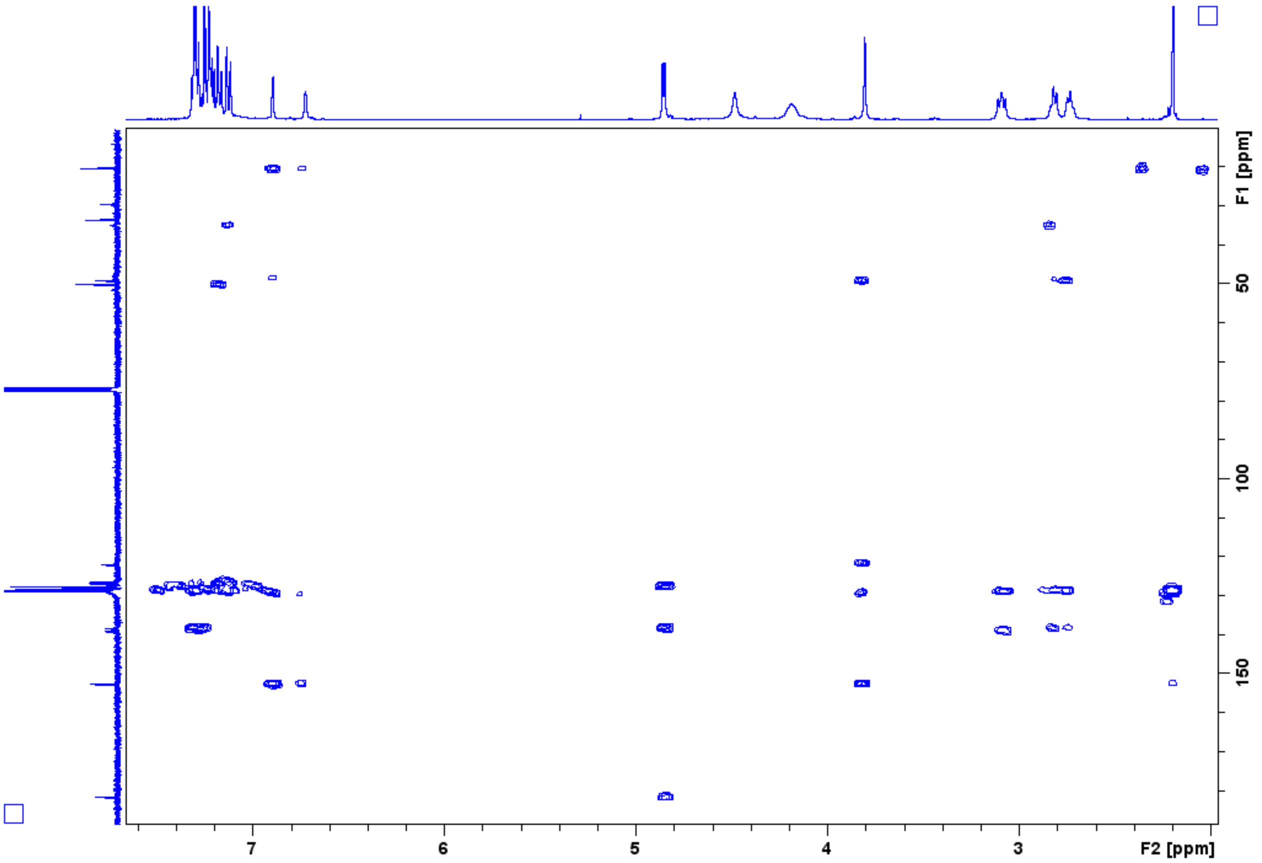

Figure S71. ^{1}H-^{13}C HMBC spectrum of compound **3cb**.

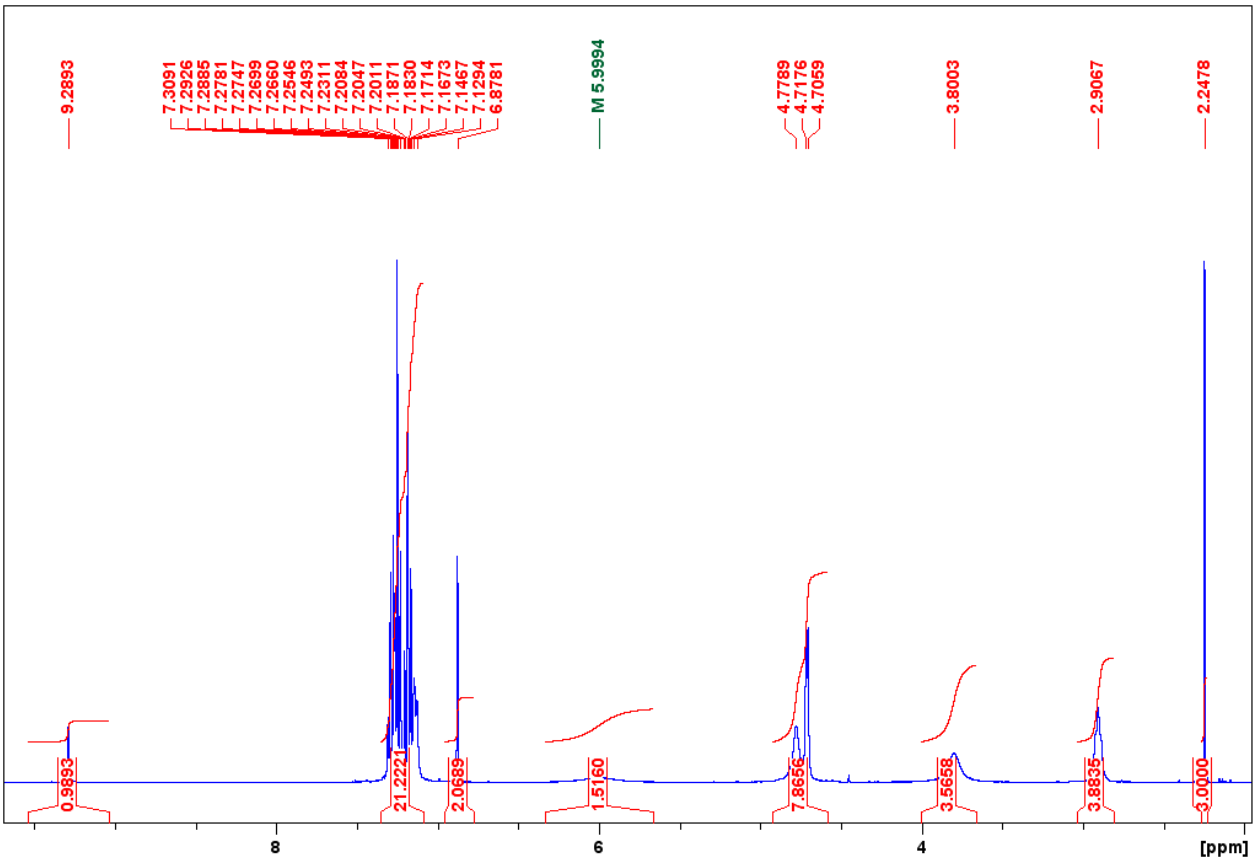

Figure S72. ^{1}H NMR spectrum of compound **4cb**.

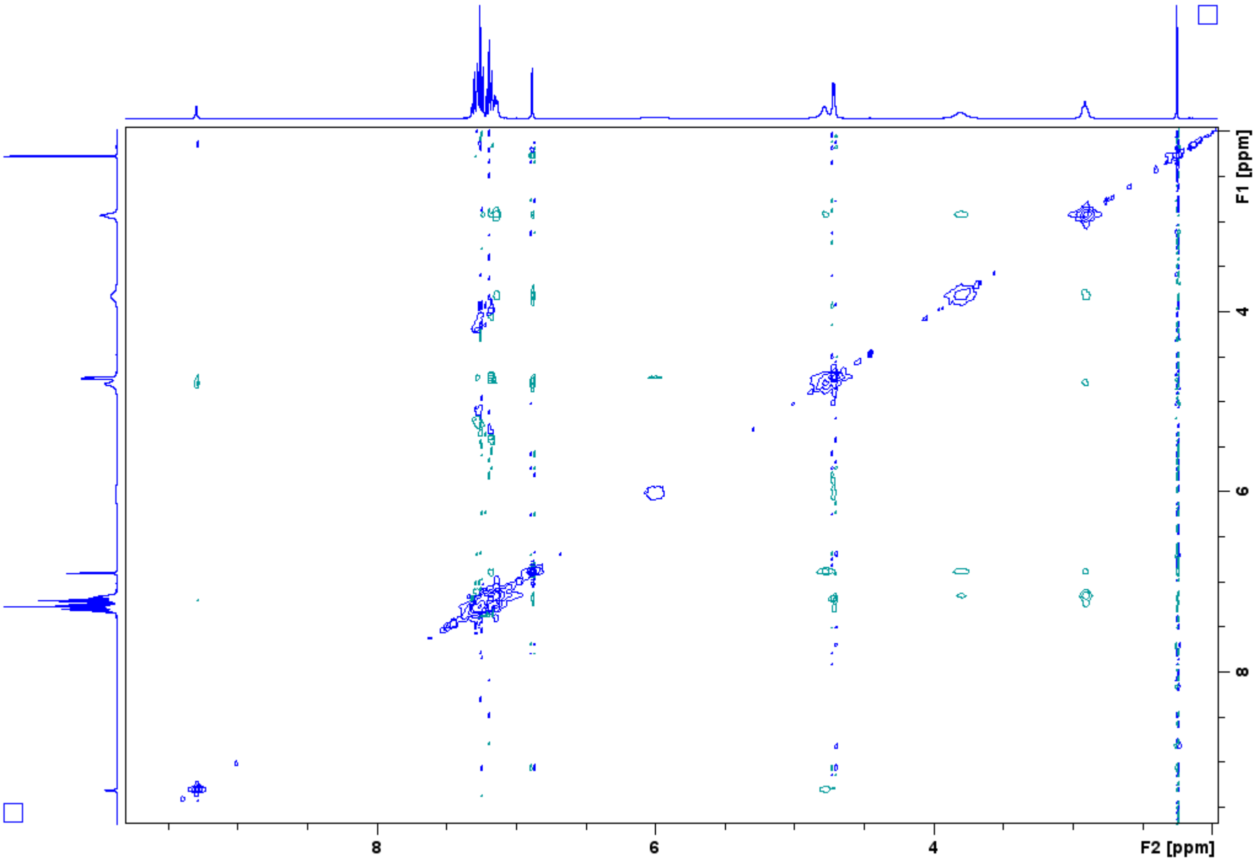

Figure S73. ^{1}H-^{1}H NOESY spectrum of compound **4cb**.

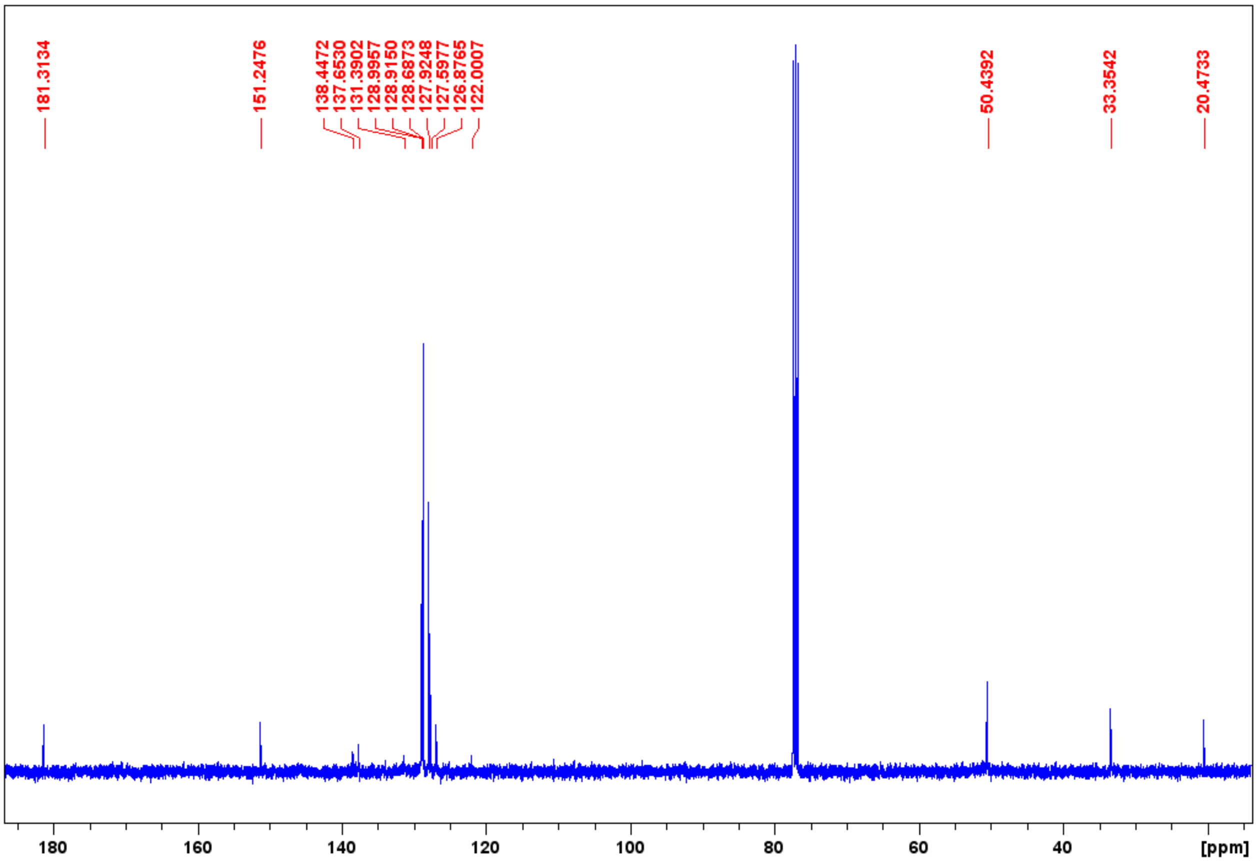

Figure S74. ^{13}C NMR spectrum of compound **4cb**.

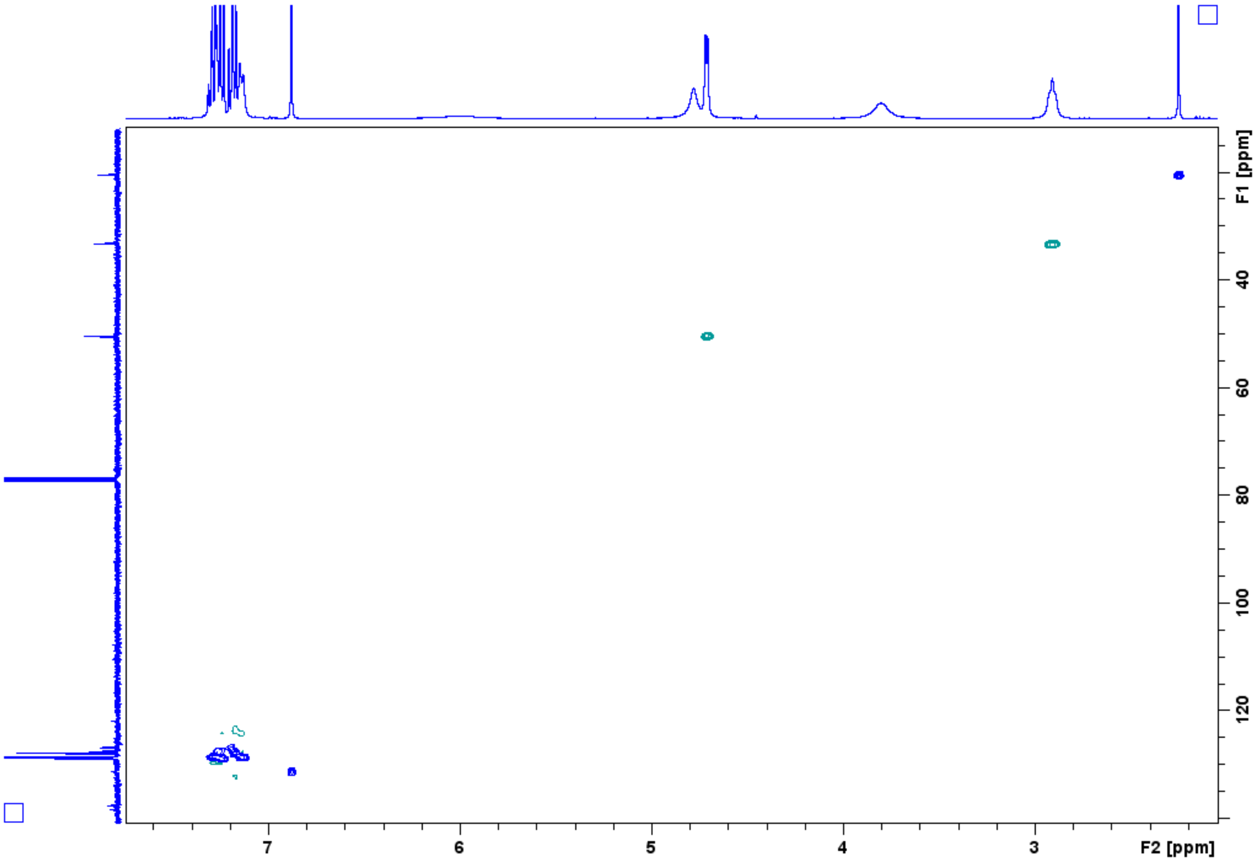

Figure S75. ^{1}H-^{13}C HSQC spectrum of compound **4cb**.

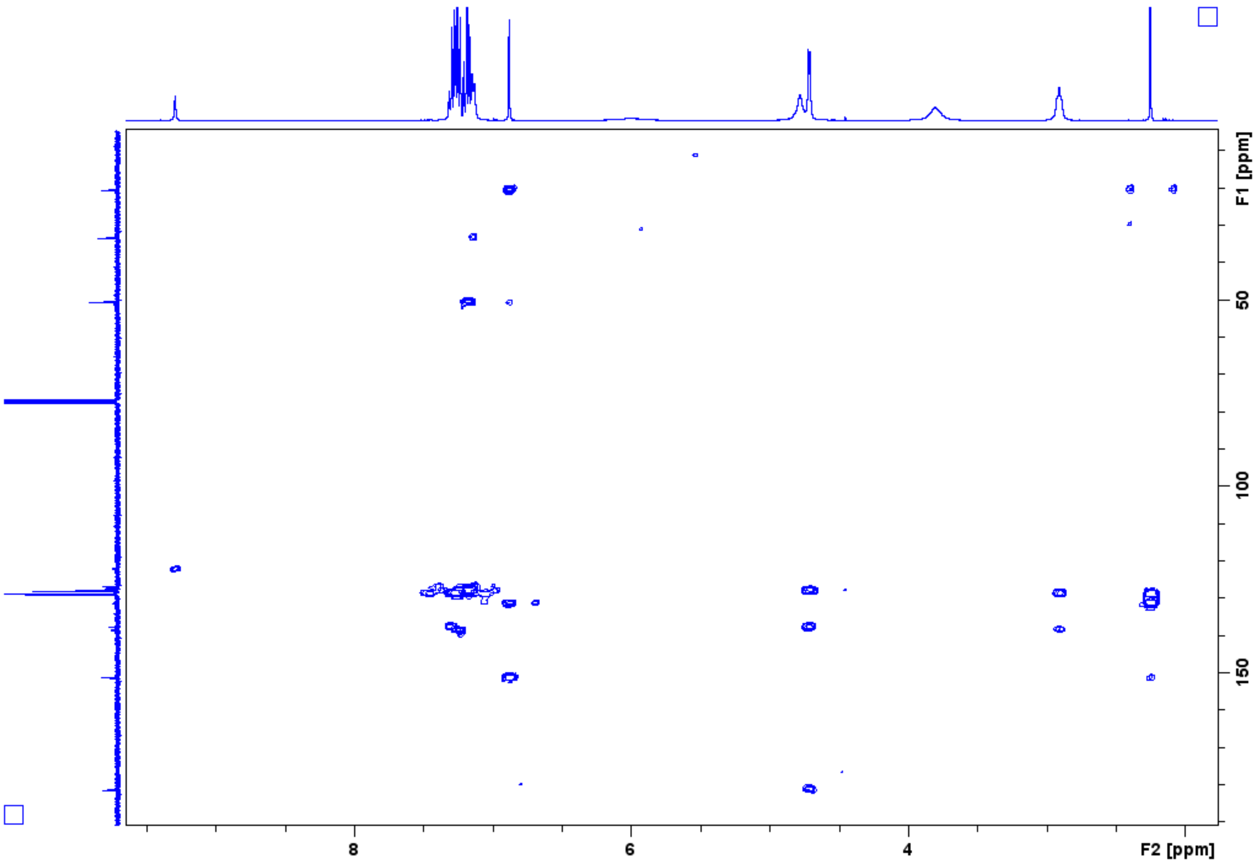

Figure S76. ^{1}H-^{13}C HMBC spectrum of compound **4cb**.

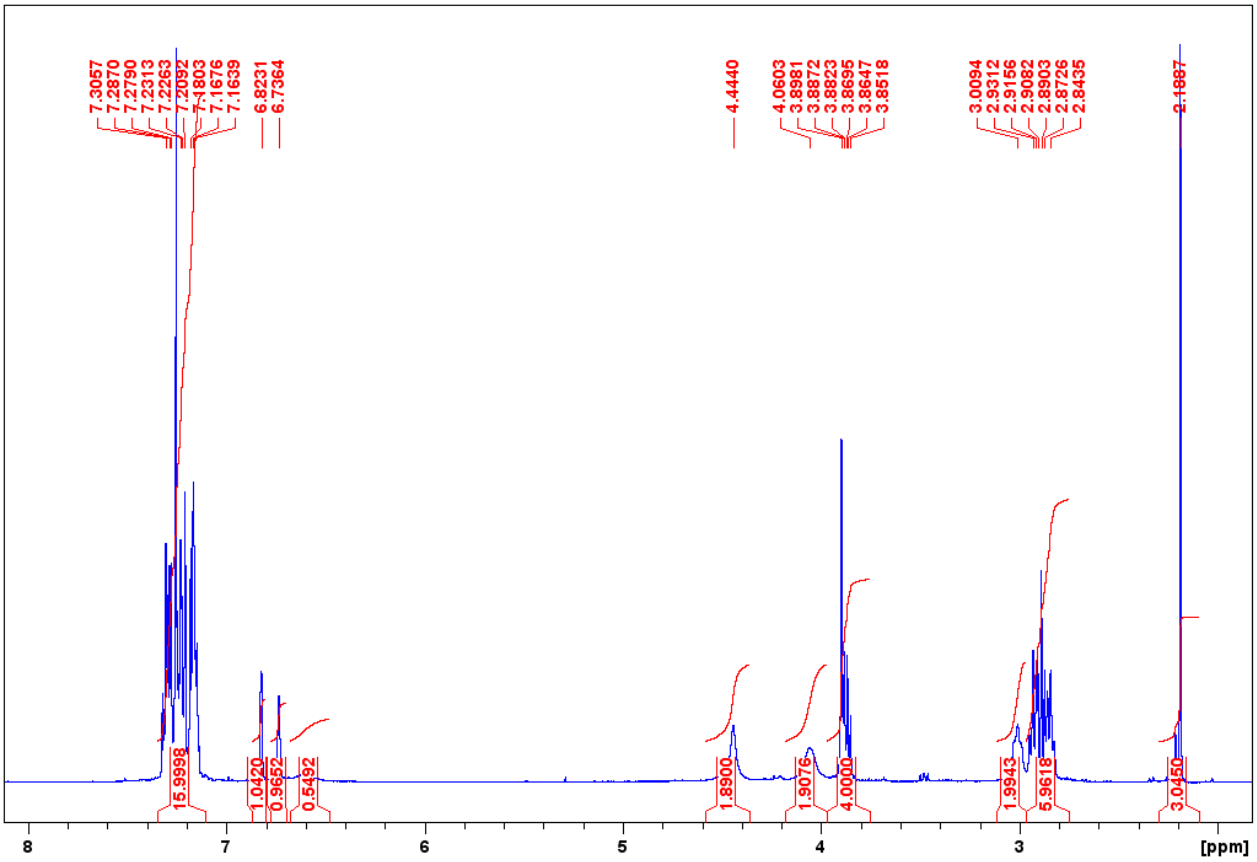

Figure S77. ^{1}H NMR spectrum of compound **3cc**.

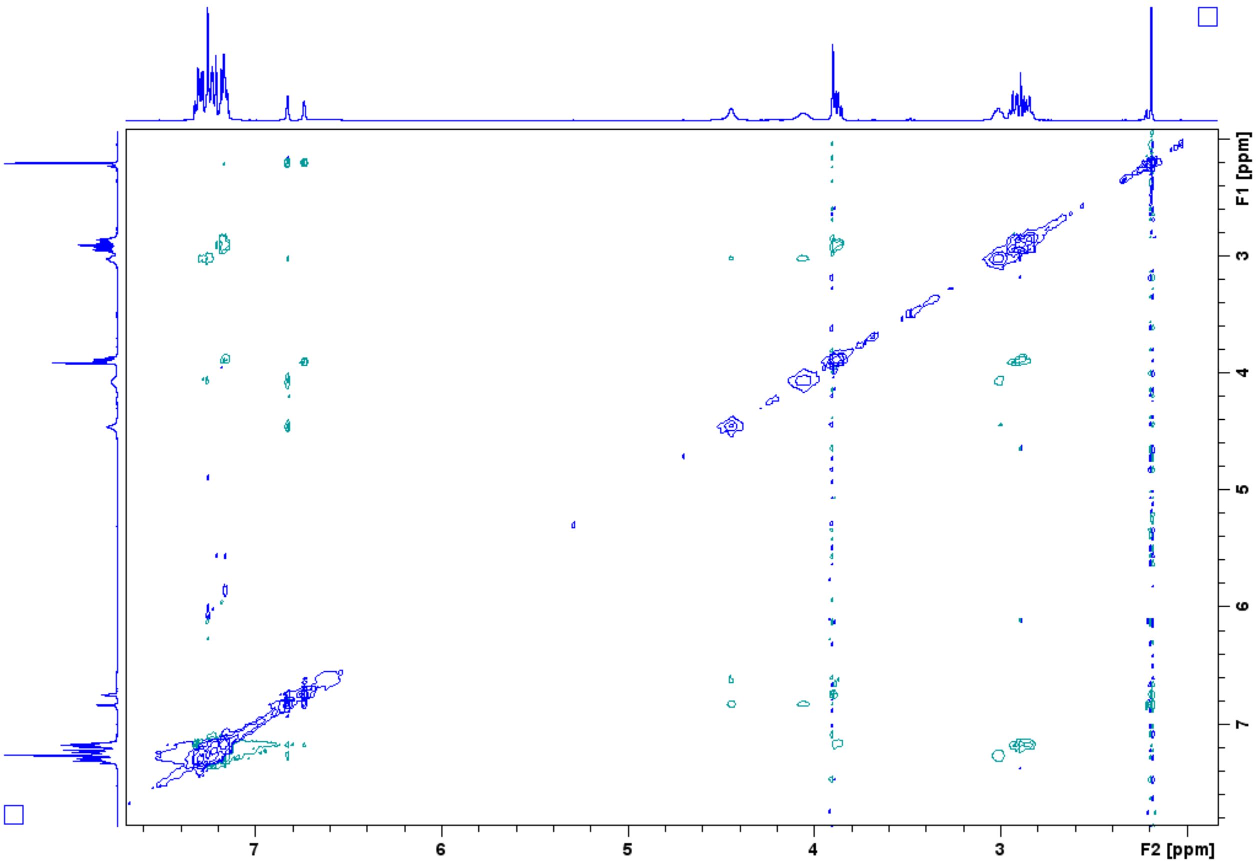

Figure S78. ^{1}H-^{1}H NOESY spectrum of compound **3cc**.

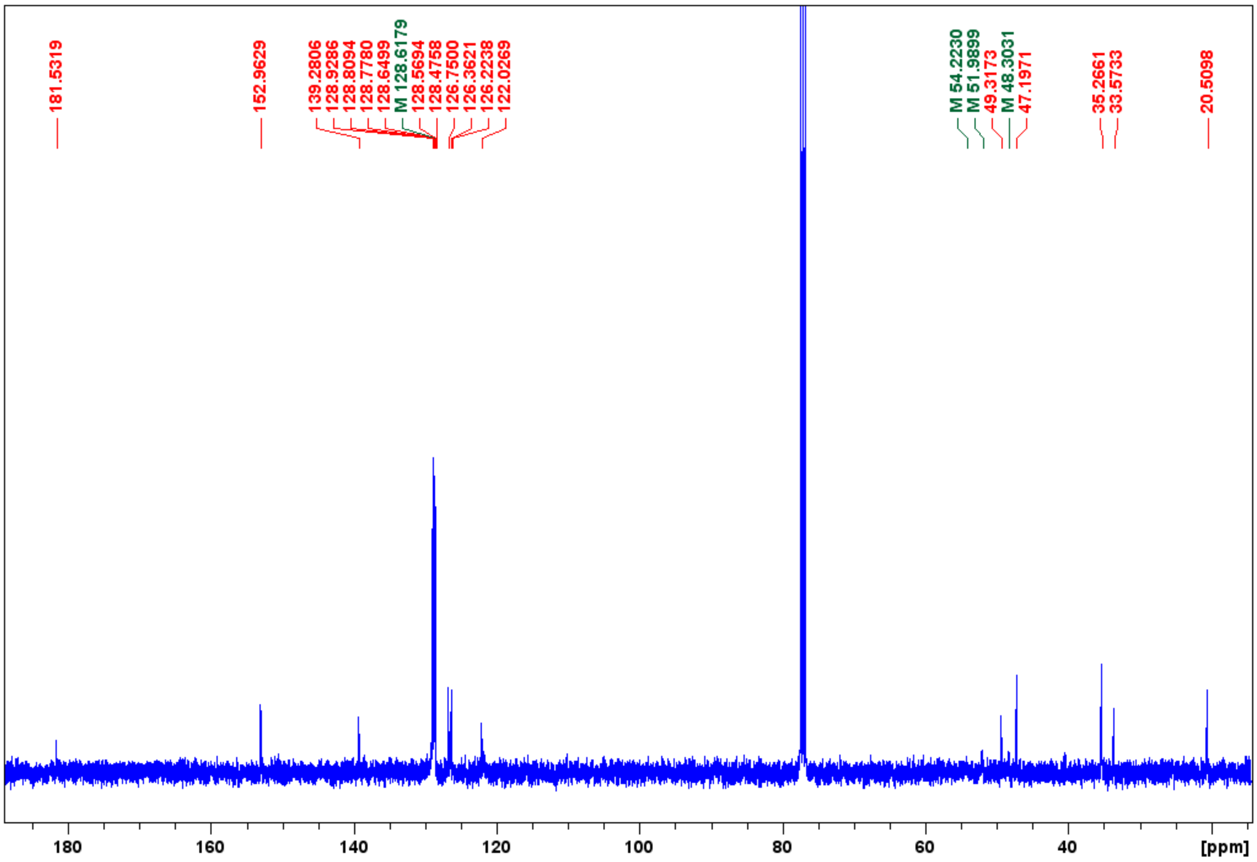

Figure S79. ^{13}C NMR spectrum of compound **3cc**.

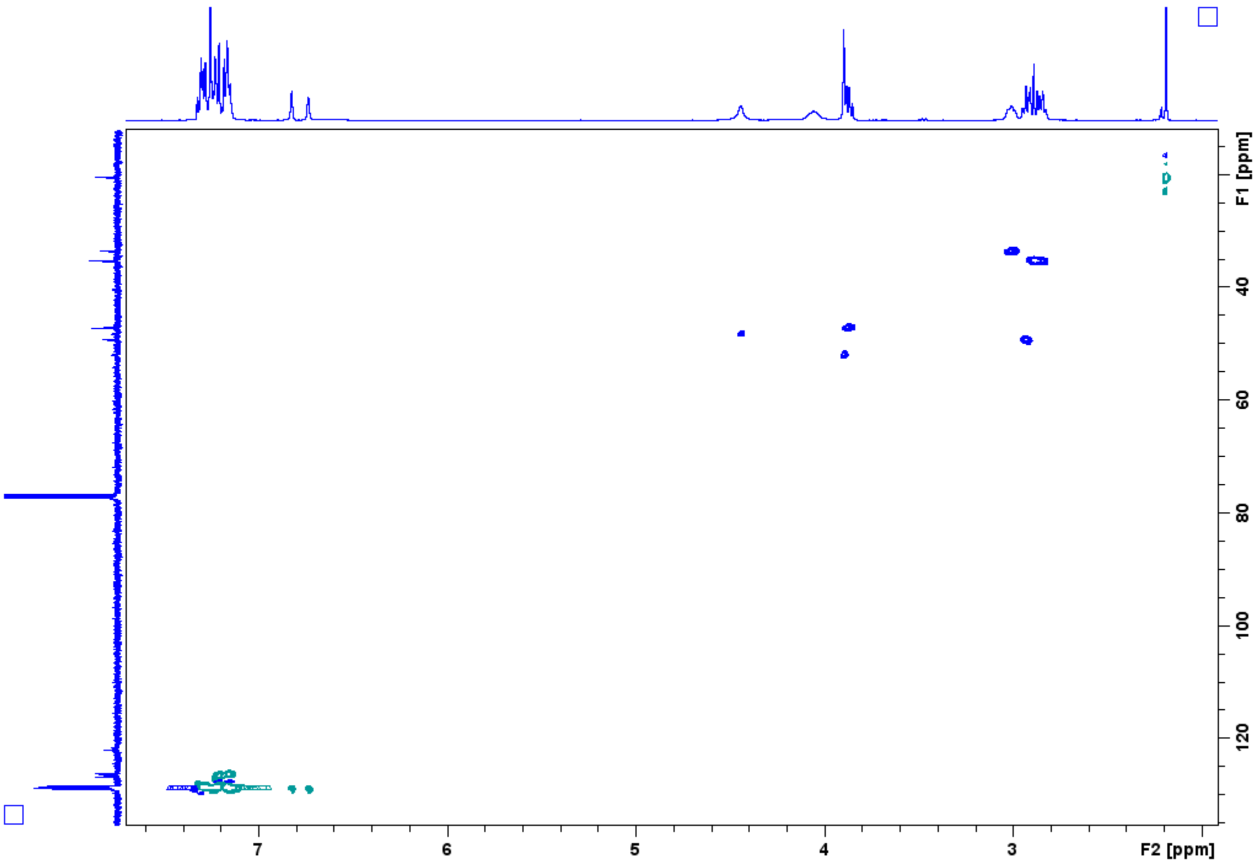

Figure S80. ^{1}H-^{13}C HSQC spectrum of compound **3cc**.

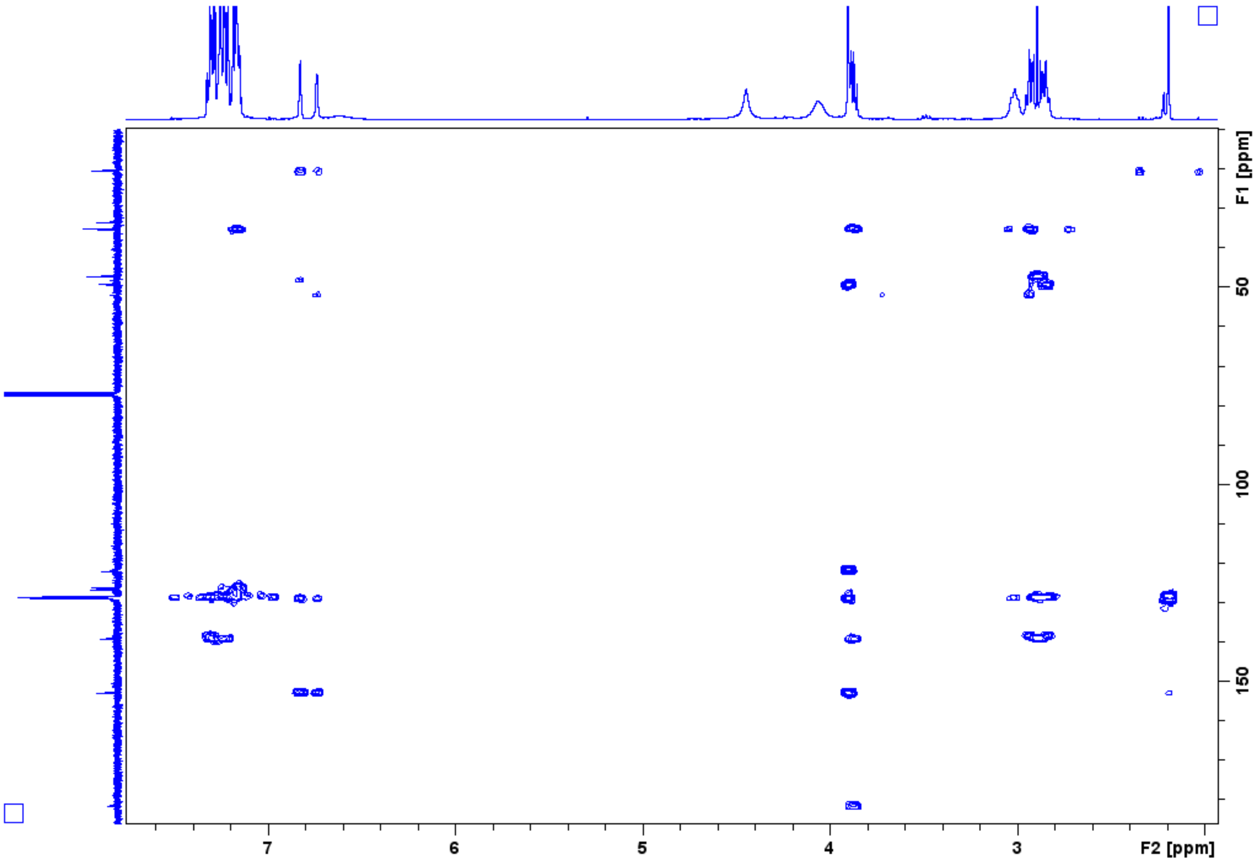

Figure S81. ^{1}H-^{13}C HMBC spectrum of compound **3cc**.

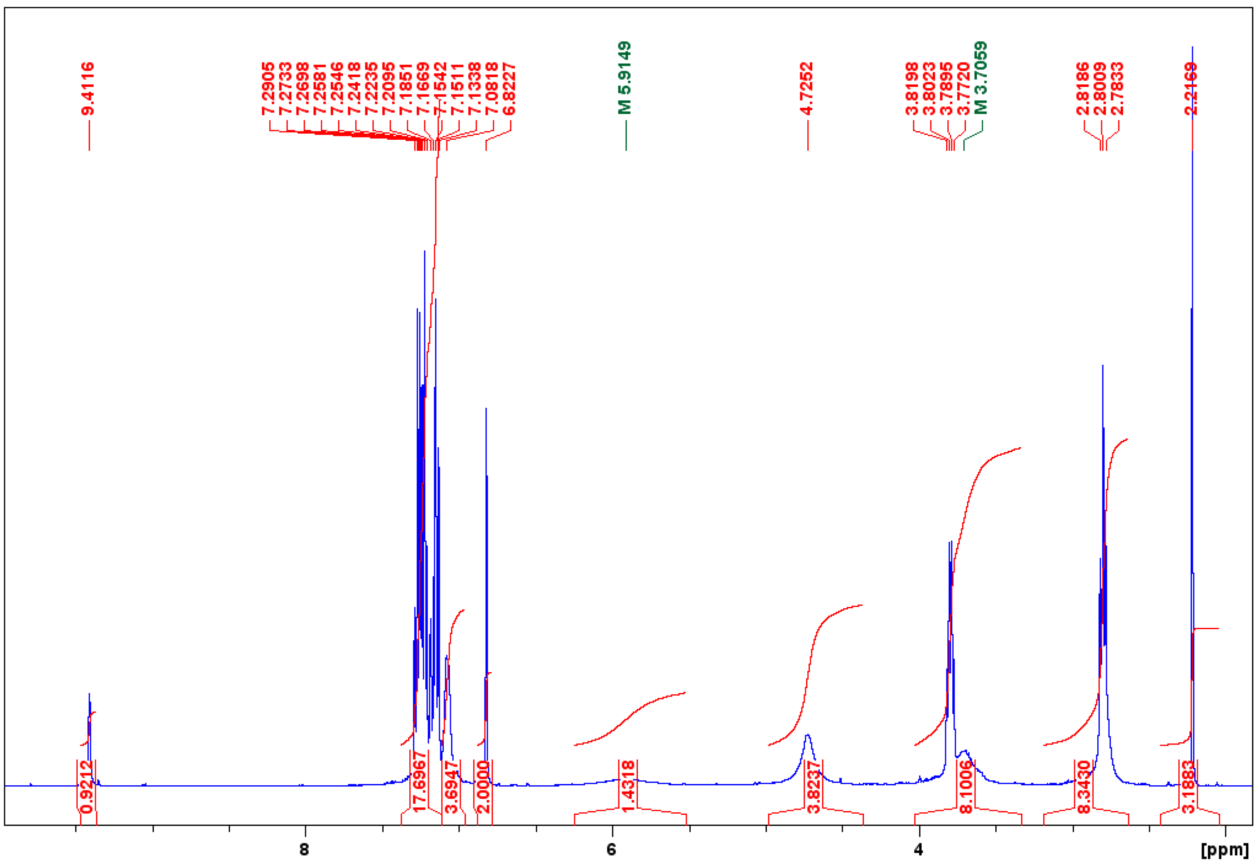

Figure S82. ^{1}H NMR spectrum of compound **4cc**.

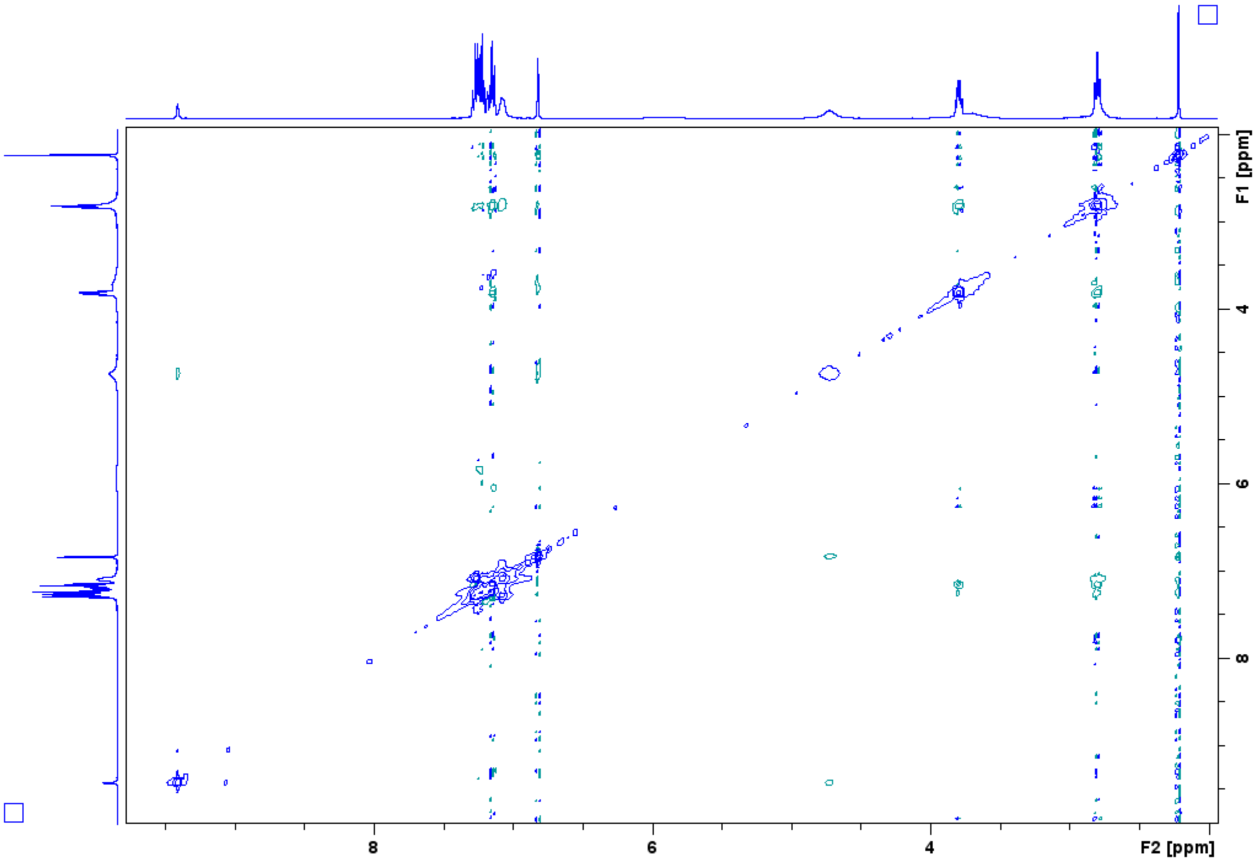

Figure S83. ^{1}H-^{1}H NOESY spectrum of compound **4cc**.

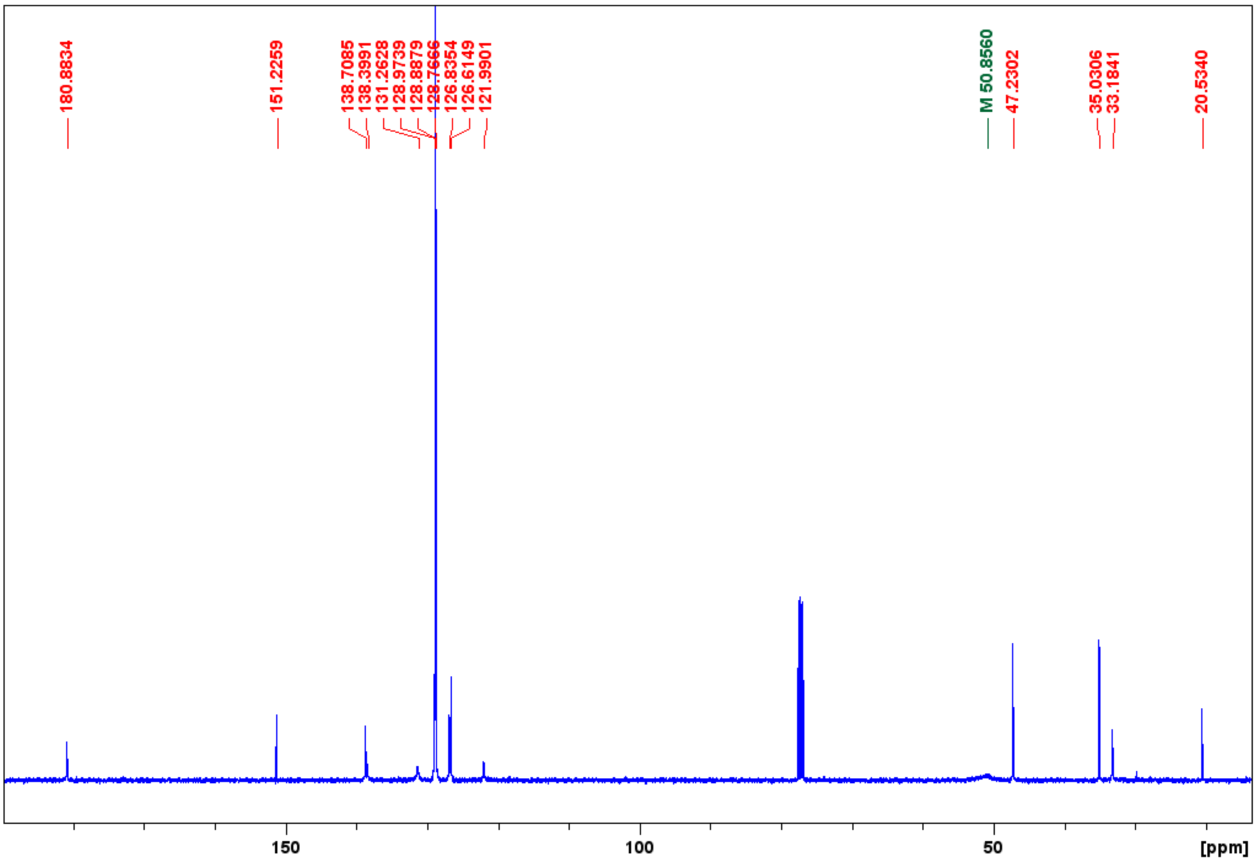

Figure S84. ^{13}C NMR spectrum of compound **4cc**.

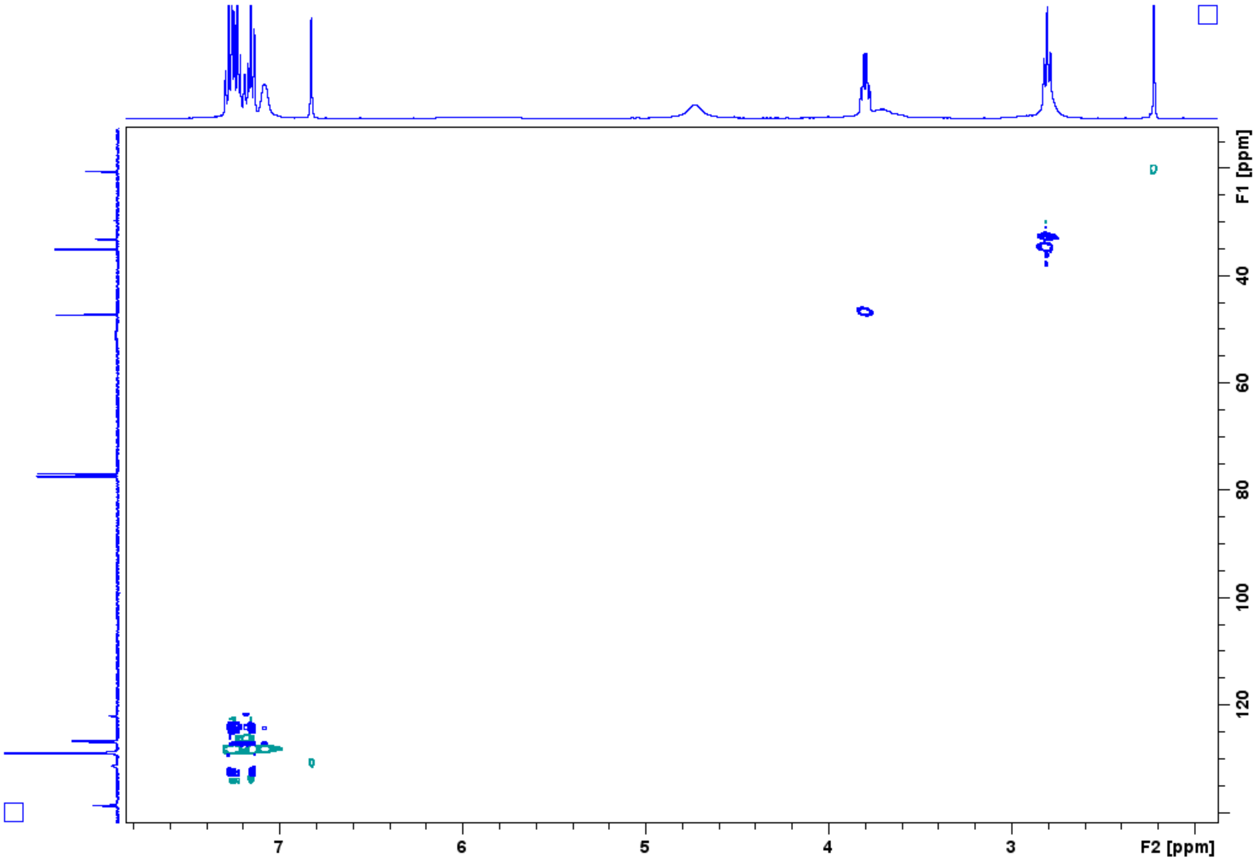

Figure S85. ^{1}H-^{13}C HSQC spectrum of compound **4cc**.

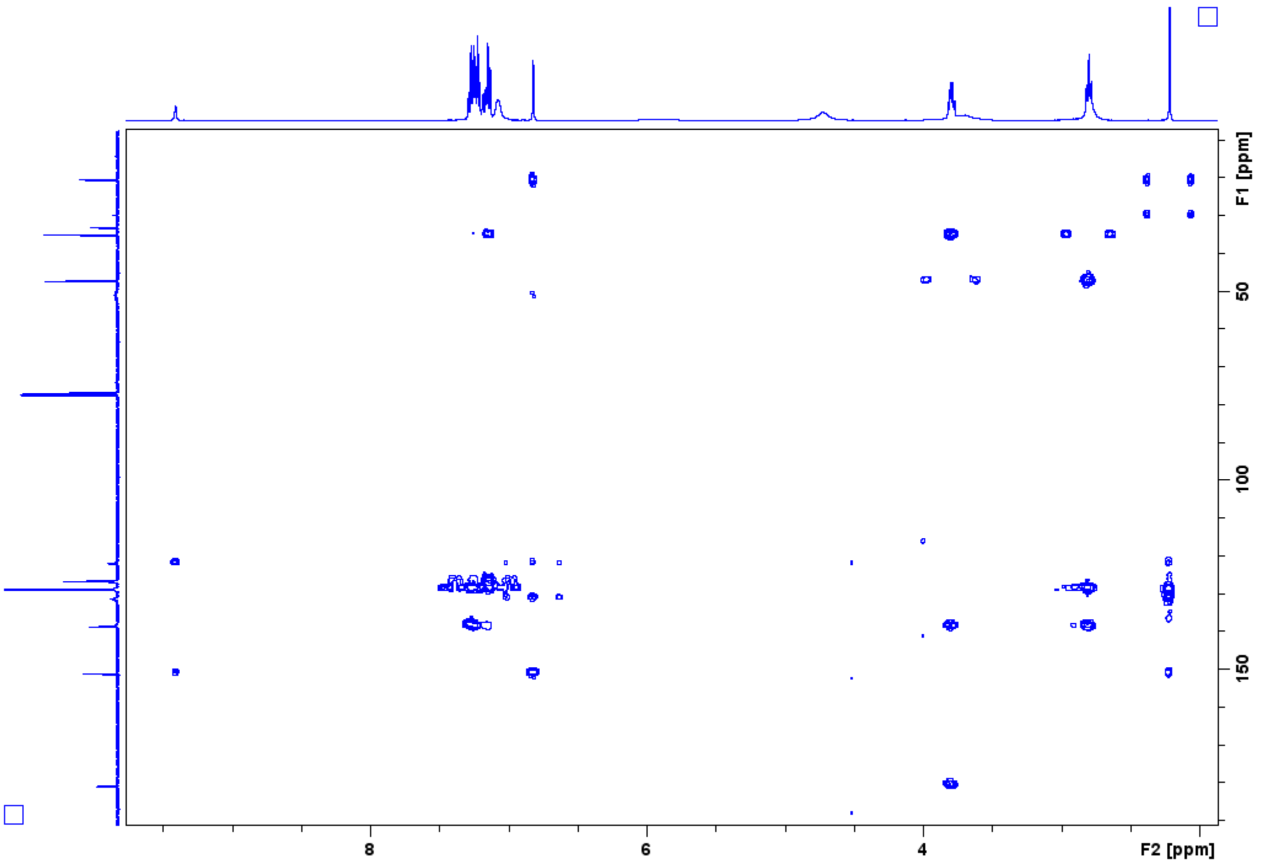

Figure S86. ^{1}H-^{13}C HMBC spectrum of compound **4cc**.

Original HRMS spectra

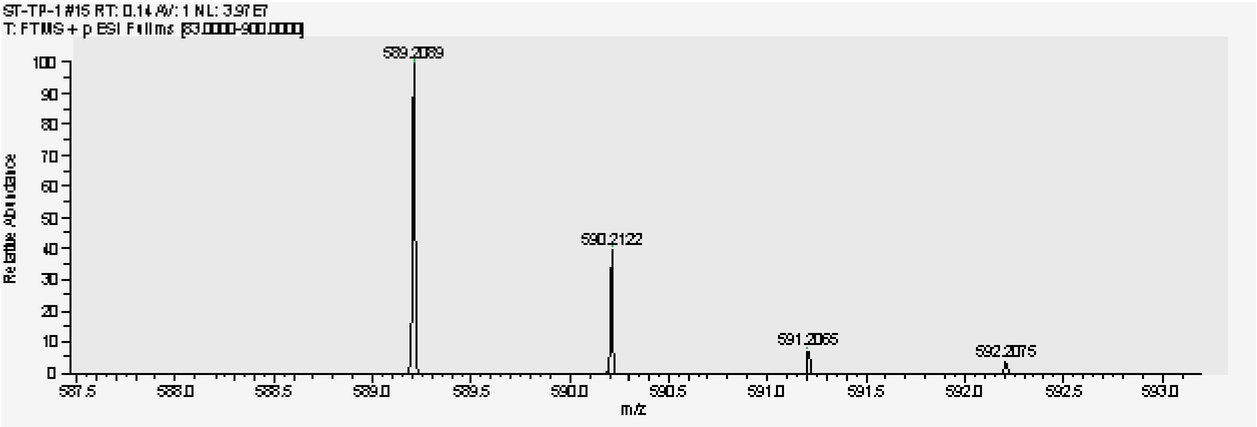

Figure S87. HRMS (HESI) spectrum of compound **4aa**.

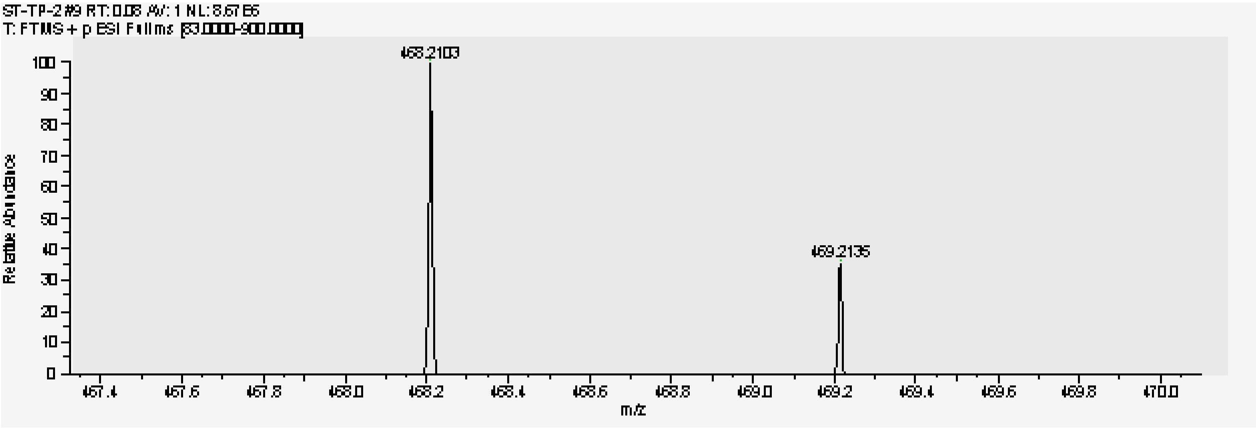

Figure S88. HRMS (HESI) spectrum of compound **3ab**.

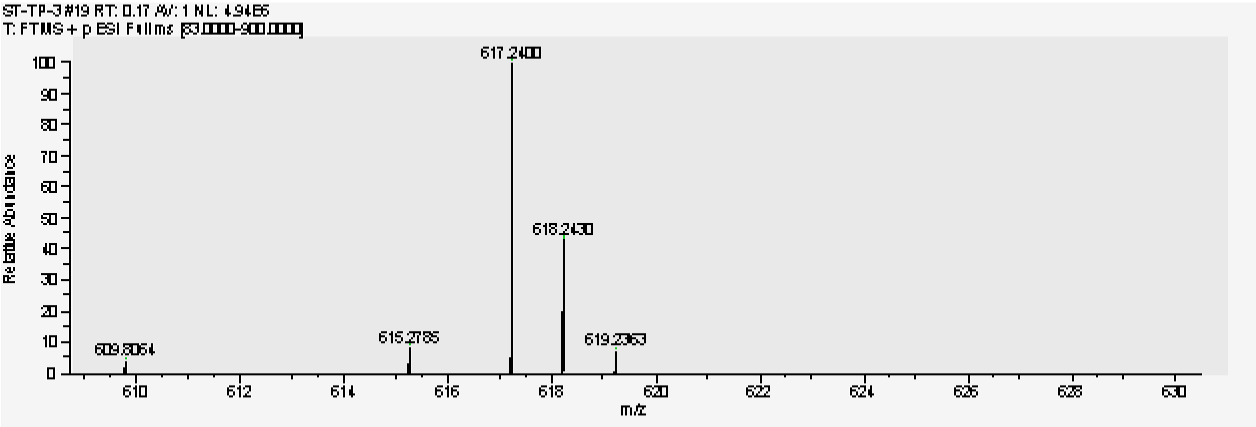

Figure S89. HRMS (HESI) spectrum of compound **4ab**.

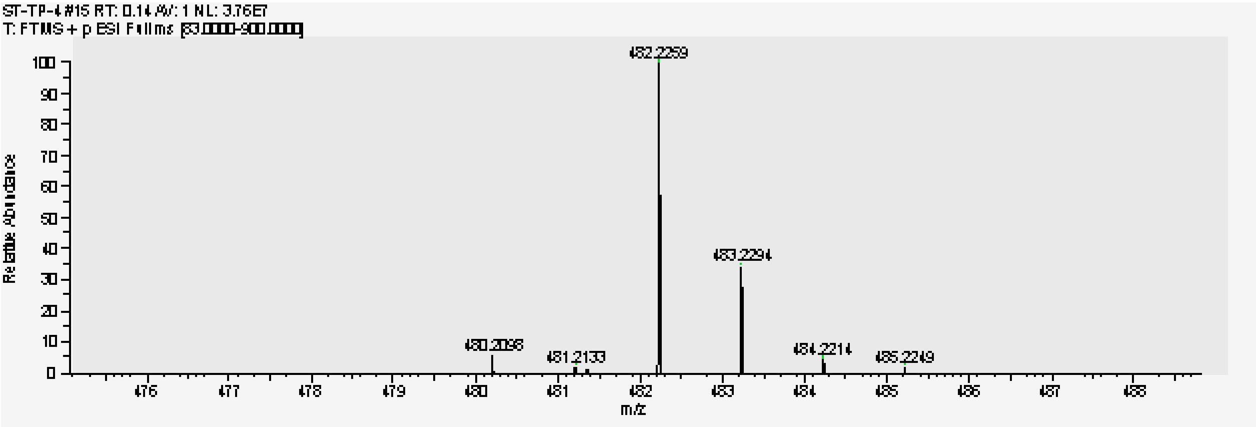

Figure S90. HRMS (HESI) spectrum of compound **3ac**.

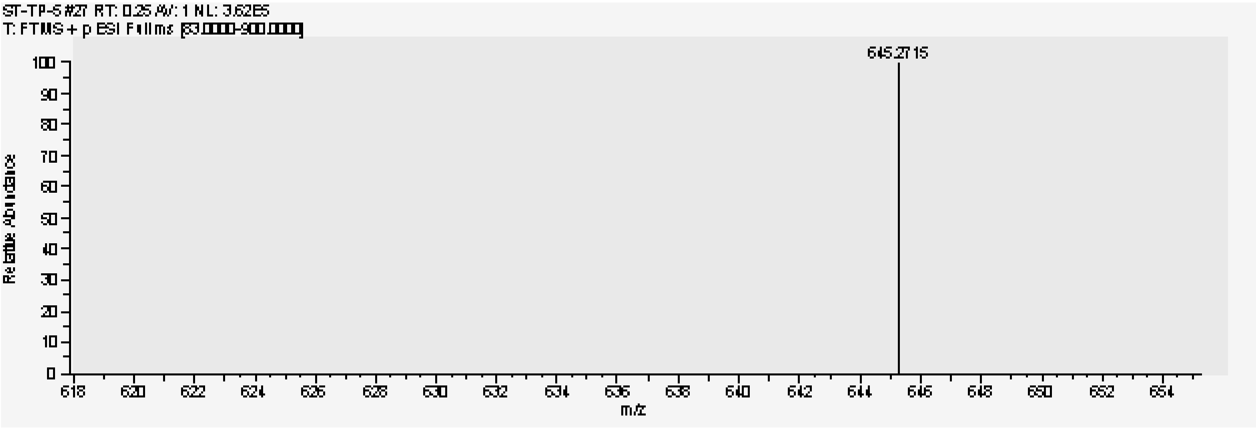

Figure S91. HRMS (HESI) spectrum of compound **4ac**.

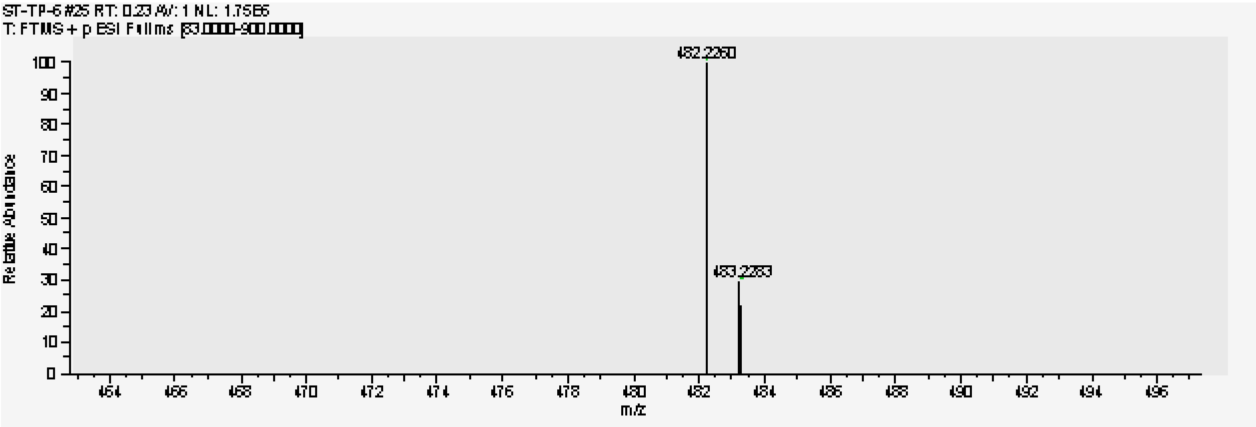

Figure S92. HRMS (HESI) spectrum of compound **3ba**.

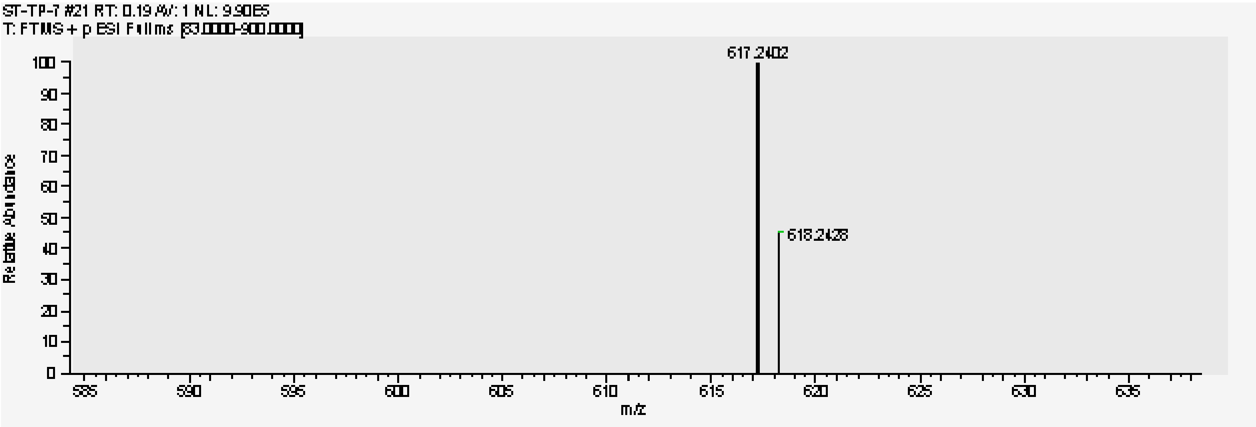

Figure S93. HRMS (HESI) spectrum of compound **4ba**.

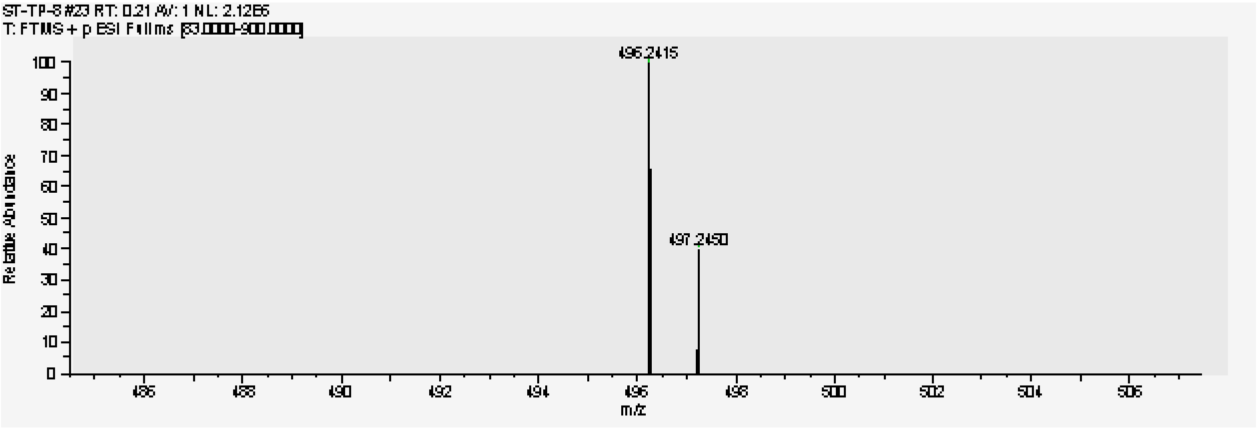

Figure S94. HRMS (HESI) spectrum of compound **3bb**.

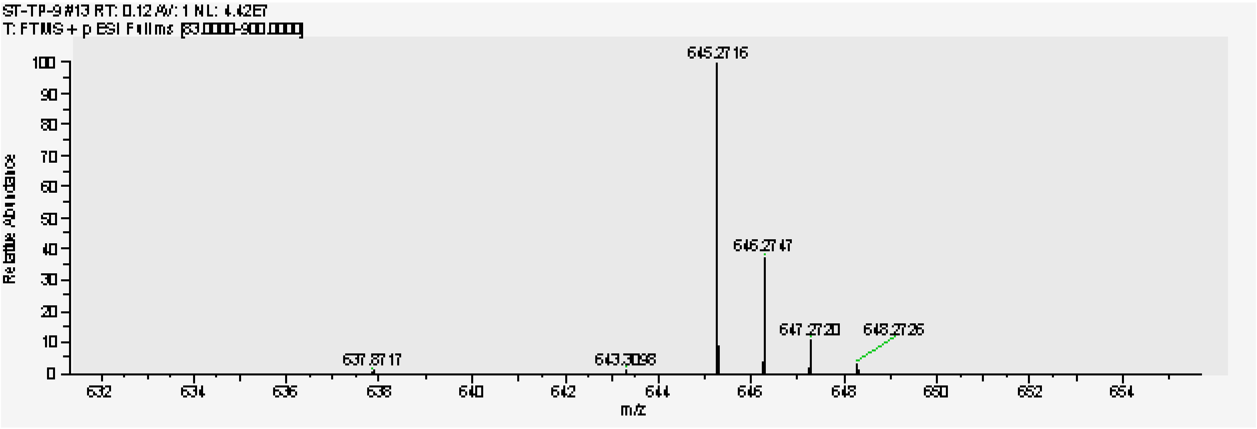

Figure S95. HRMS (HESI) spectrum of compound **4bb**.

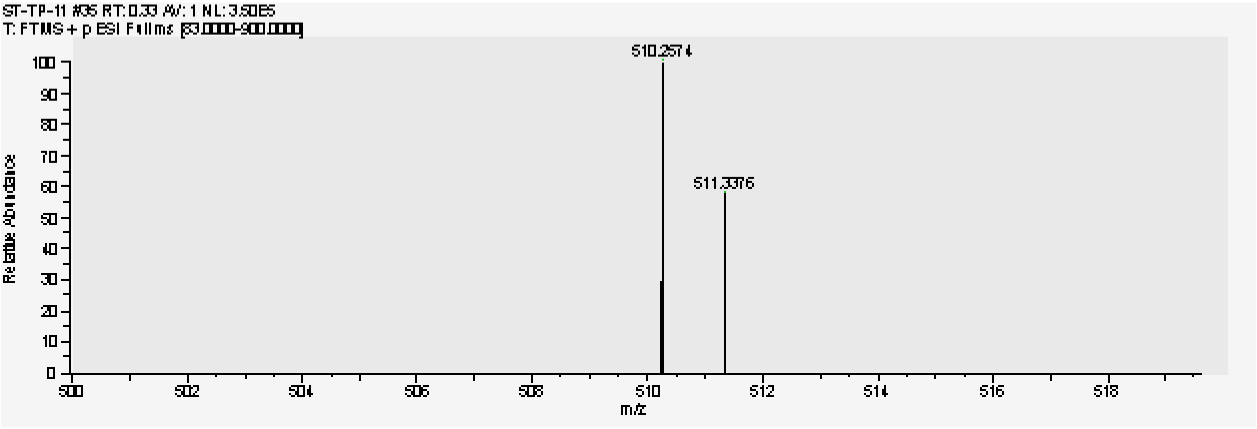

Figure S96. HRMS (HESI) spectrum of compound **3bc**.

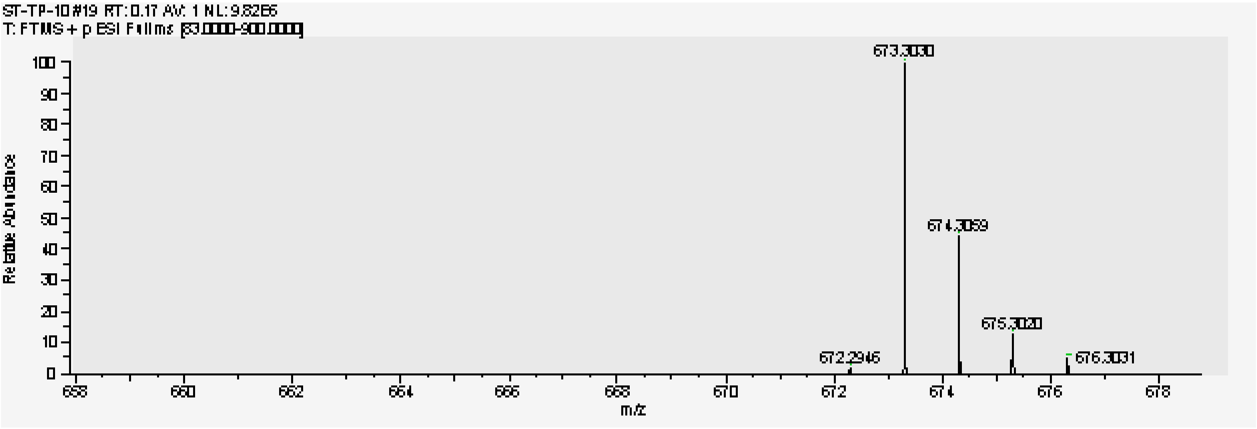

Figure S97. HRMS (HESI) spectrum of compound **4bc**.

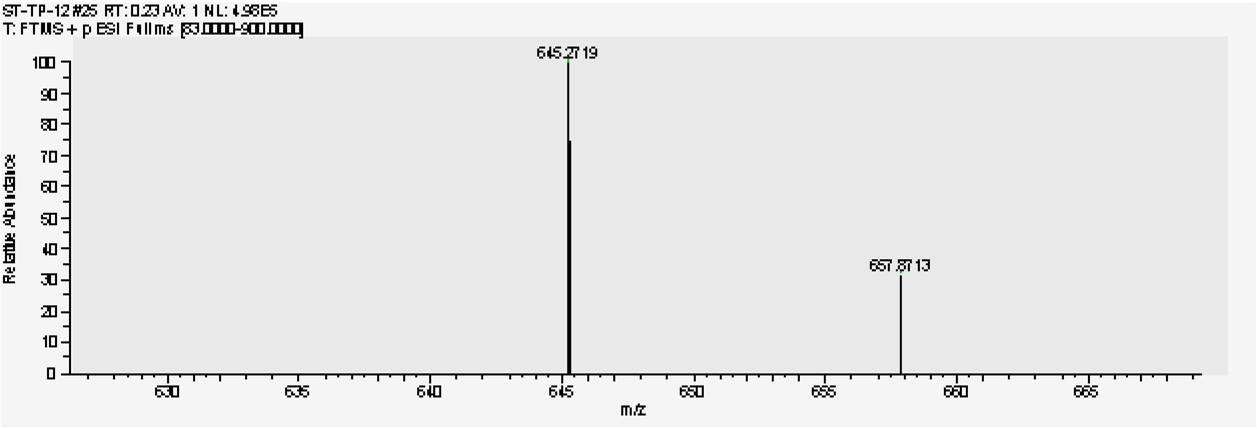

Figure S98. HRMS (HESI) spectrum of compound **4ca**.

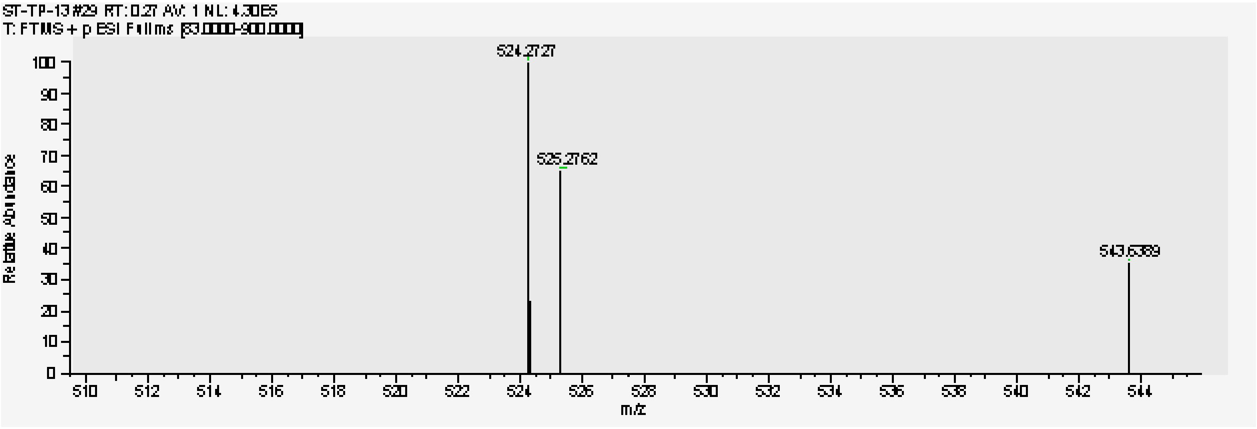

Figure S99. HRMS (HESI) spectrum of compound **3cb**.

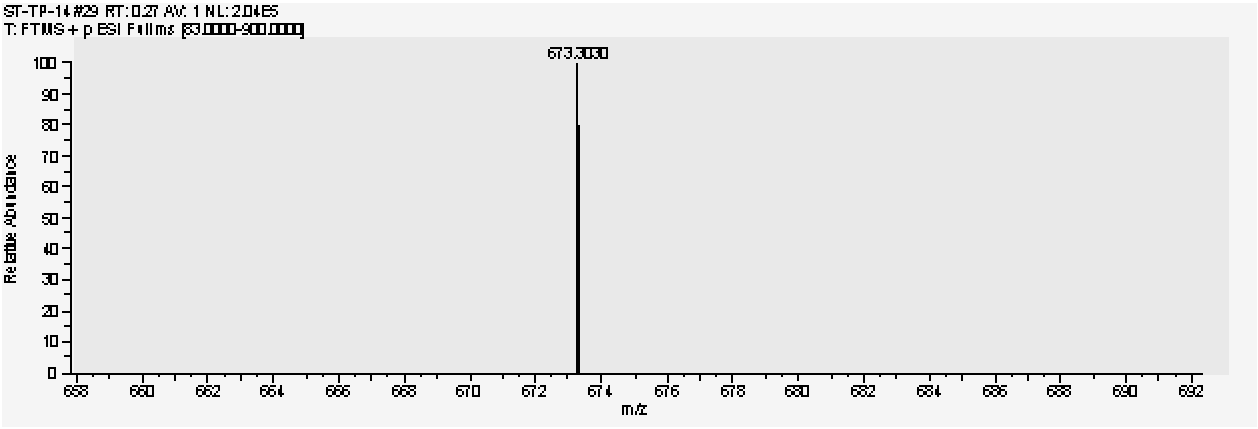

Figure S100. HRMS (HESI) spectrum of compound **4cb**.

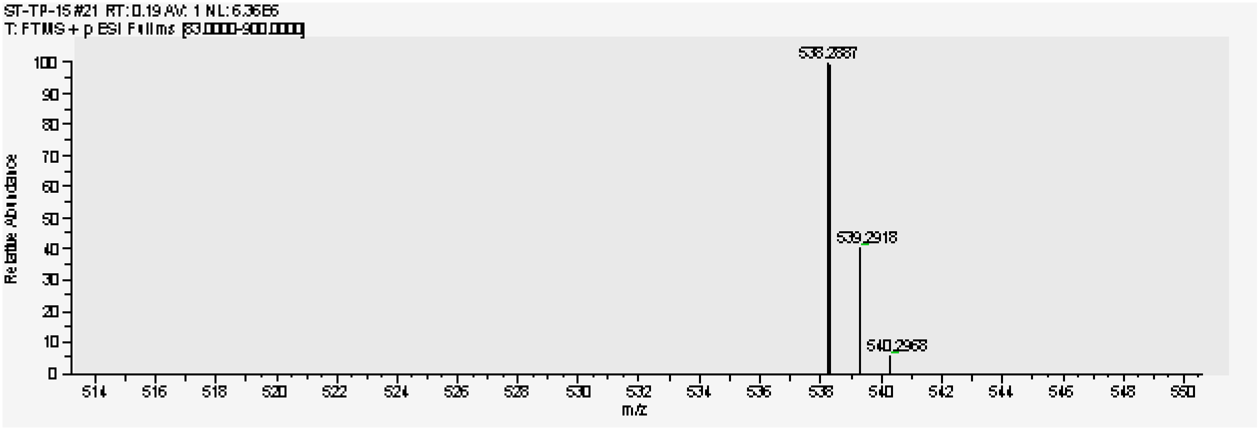

Figure S101. HRMS (HESI) spectrum of compound **3cc**.

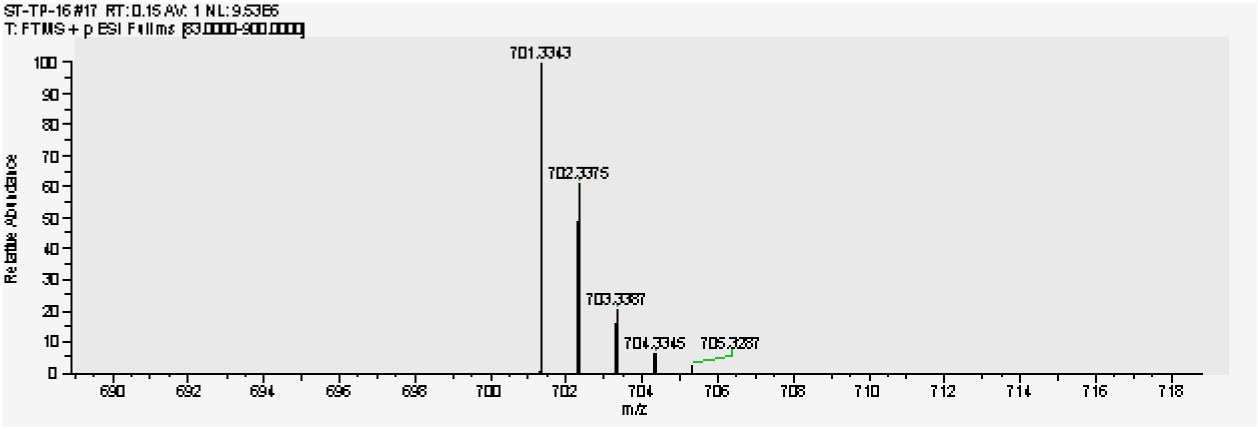

Figure S102. HRMS (HESI) spectrum of compound **4cc**.
